# Radiation‐Induced Tumor‐Intrinsic LTβR N‐Glycosylation Suppresses Pyroptosis Through TRIM28‐Mediated PCBP2 SUMOylation to Promote Gastric Cancer Radioresistance

**DOI:** 10.1002/advs.76157

**Published:** 2026-06-22

**Authors:** Weijie Zang, Yunlong Ji, Chunwang Zhou, Xiwen Li, Zhuang Lu, Guangze Zhang, Yu Chen, Wanjiang Xue, Yilin Hu

**Affiliations:** ^1^ Department of Gastrointestinal Surgery Affiliated Hospital of Nantong University Medical School of Nantong University Nantong Jiangsu China; ^2^ Research Center of Clinical Medicine Affiliated Hospital of Nantong University Nantong Jiangsu China; ^3^ Nantong Key Laboratory of Gastrointestinal Oncology Nantong Jiangsu China; ^4^ Department of Central Laboratory, Kunshan Hospital of Chinese Medicine Affiliated Hospital of Yangzhou University Kunshan Jiangsu China

**Keywords:** gastric cancer, LTβR, pyroptosis, radioresistance, translational reprogramming

## Abstract

Radiotherapy is important for advanced and metastatic gastric cancer (GC), but radioresistance limits its benefit. Pyroptosis has emerged as a potential strategy to overcome radioresistance, yet its regulatory mechanisms remain unclear. Using LC‐MS/MS‐based proteomic profiling of tumor tissues from patients with GC treated with neoadjuvant chemoradiotherapy, we identified tumor‐intrinsic lymphotoxin beta receptor (LTβR), previously considered mainly an immune cell membrane protein, as a candidate determinant of poor radiotherapy response. Functional studies in GC cell lines, xenografts, and patient‐derived organoids (PDOs) showed that *LTβR* depletion enhanced radiosensitivity, whereas *LTβR* overexpression promoted radioresistance. Integrated RNA‐seq, Ribo‐seq, and polysome profiling showed that *LTβR* loss reduced translational efficiency of *SARM1* under irradiation. Mechanistically, irradiation increased LTβR stability in a glycosylation‐dependent manner and promoted nuclear translocation. In the nucleus, LTβR enhanced TRIM28‐mediated SUMOylation of PCBP2, promoting cytoplasmic redistribution of PCBP2 and increased translational efficiency of *SARM1*. Consistently, *LTβR* suppressed irradiation‐induced pyroptosis through the NLRP3/caspase‐1/GSDMD pathway. We further identified EMD638683 as an LTβR‐binding compound through structure‐based virtual screening, and showed that cRGD‐modified liposomes improved its tumor‐targeted delivery and enhanced LTβR reduction, radiosensitization, and tumor suppression in PDO and xenograft models. Together, these findings highlight LTβR as a promising therapeutic target to improve radiotherapy efficacy in GC.

## Graphical abstract

Radiotherapy triggers LTβR N‐glycosylation, enhancing its overall protein stability and nuclear retention. This accumulation drives TRIM28‐mediated PCBP2 SUMOylation, suppressing pyroptosis and conferring gastric cancer radioresistance. Therapeutically, a targeted nanoplatform (cRGD‐Lipo@EMD) effectively disrupts this regulatory axis, offering a highly potent and clinically translatable strategy to overcome radiotherapy resistance in gastric cancer.

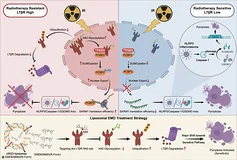

## 1 Introduction

Gastric cancer (GC) is the fifth most frequently diagnosed cancer worldwide and the fifth leading cause of cancer‐related mortality [1]. In China, about 70.8% of patients present with advanced disease, and the overall 5‐year survival rate remains under 30% [2]. Metastatic disease accounts for approximately 15% of cases [3]. Systemic treatment for advanced GC is based primarily on immunotherapy, targeted therapy, and chemotherapy. Radiotherapy also remains an important component of multidisciplinary management because it provides local control and symptom relief in patients with positive margins, incomplete resection, unresectable disease at presentation, local recurrence without distant metastasis, oligometastatic disease, or symptoms such as bleeding, pain, and obstruction [4, 5, 6, 7]. Its clinical benefit, however, is often constrained by radioresistance. Research on radioresistance in GC has focused largely on aberrant DNA damage repair, dysregulation of cell cycle checkpoints, evasion of apoptosis, tumor hypoxia, and epithelial to mesenchymal transition [8]. More recent studies identify pyroptosis as an important component of radiation‐induced cell injury and adaptive resistance, raising the possibility that restoration of pyroptosis may improve radiosensitivity [9, 10, 11, 12, 13, 14].

Pyroptosis is a lytic form of programmed cell death that is defined by membrane pore formation, cell swelling, and the release of intracellular mediators, thereby eliciting a pronounced inflammatory response. Gasdermins execute this process after proteolytic activation by forming membrane pores that permit the release of IL‐1β, IL‐18, and damage‐associated molecular patterns [15]. Radiotherapy can induce pyroptosis through GSDME‐ or GSDMD‐dependent programs and, in turn, enhance antitumor immunity and influence treatment response [12, 16]. Tumor cells can also suppress pyroptosis by modulating the STING‐NLRP3 axis, gasdermin stability, and upstream stress signaling, thereby promoting an immunosuppressive microenvironment and radioresistance [17, 18]. The extent and biological consequences of pyroptotic signaling are not determined solely by protein abundance or caspase activation. Post‐translational modification is now recognized as an important regulatory layer of this process. Multiple post‐translational modifications have been implicated in pyroptosis, including phosphorylation, acetylation, ubiquitylation, SUMOylation, palmitoylation, prenylation, succination, lactylation, and ADP‐ribosylation, which regulate pyroptosis through gasdermin‐dependent or gasdermin‐independent mechanisms [19]. Among these modifications, SUMOylation remains relatively understudied. SUMOylation is the reversible conjugation of SUMO1, SUMO2, or SUMO3 to lysine residues on substrate proteins through the coordinated action of SAE1/UBA2, UBC9, and E3 ligases [20]. This modification participates in DNA damage response, stress adaptation, chromatin homeostasis, and double‐strand break repair, all of which are closely linked to radiotherapy response [21, 22, 23]. Our group and others have also linked SUMOylation to chemotherapy resistance in GC [24, 25, 26]. Existing evidence further connects SUMOylation to pyroptosis in inflammatory and fibrotic diseases, with SUMO‐related regulation of NLRP3, GSDMD, and Gli1 influencing pyroptotic signaling and disease progression [27, 28, 29, 30]. Whether SUMOylation also directs pathway choice during radiotherapy‐induced pyroptosis and contributes to radiosensitivity in tumors remains unclear.

Growing evidence indicates that molecules once thought to function mainly in immune cells can also be aberrantly upregulated in tumor cells and acquire cell‐intrinsic functions. This has been reported for PD‐1, FOXP3, and complement‐related molecules [31, 32, 33, 34, 35, 36]. For example, tumor‐intrinsic complement signaling, defined here as signaling cascades operating strictly within the tumor cell compartment rather than the surrounding microenvironment, can drive malignant progression, therapy resistance, and remodeling of the tumor microenvironment [31, 36, 37]. This indicates that aberrant overexpression of immune‐related molecules in tumor cells is functionally relevant rather than incidental. Lymphotoxin beta receptor (LTβR; encoded by the *LTBR* gene) is one such molecule. It is a context‐dependent member of the TNF receptor superfamily with established roles in immune organization and homeostasis [38]. Preclinical studies suggest that activation of LTβR can enhance antitumor immunity [39, 40, 41, 42, 43, 44]. Clinical studies of LTβR agonists, however, have not shown the expected efficacy, underscoring the context dependence of its function. Beyond its roles in the microenvironment, expression of LTβR in tumor cells is increasingly linked to tumor progression, including hepatocarcinogenesis [45, 46, 47, 48, 49]. Our previous work in GC also suggests that LTβR contributes to tumor progression and metastasis [50, 51]. In addition, expression of LTβR in radioresistant resident cells has been linked to formation of local inflammatory niches, supporting a possible connection between LTβR and radiotherapy‐related responses [52]. Because radiotherapy not only directly damages tumor cells but also elicits substantial inflammatory stress and microenvironmental remodeling, it remains important to determine whether LTβR acts as a stress‐responsive factor in adaptive reprogramming after radiotherapy in GC and whether it influences pyroptosis and radiosensitivity.

Here, we identify LTβR as a candidate determinant of radioresistance in GC. We show that LTβR is elevated in radioresistant tumors and functionally contributes to reduced irradiation response in GC cells, xenografts, and patient‐derived organoids. Mechanistically, our data support a model in which LTβR limits IR‐induced pyroptotic signaling associated with the NLRP3/caspase‐1/GSDMD axis by sustaining SARM1 translation through TRIM28‐dependent SUMOylation and cytoplasmic redistribution of PCBP2. We further identify EMD638683 (EMD) as a compound that interferes with LTβR glycosylation‐associated stability and show that cRGD‐Lipo@EMD enhances radiosensitivity in vivo. Collectively, these findings reveal a noncanonical LTβR‐associated signaling program linked to adaptive radioresistance and provide a preclinical basis for exploring LTβR‐targeted radiosensitization in GC.

## 2 Results

### 2.1 LTβR Is Associated With Radioresistance in GC and Promotes Radioresistance In Vitro

**FIGURE 1:**
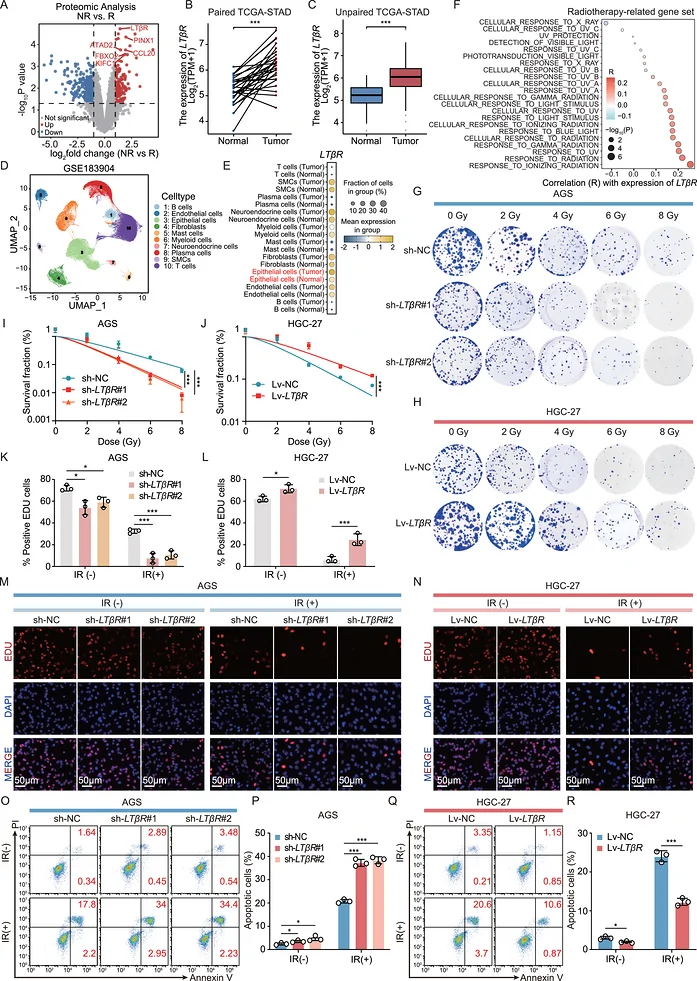
LTβR is associated with radioresistance in GC and promotes radioresistance in vitro. (A) Volcano plot of proteomic profiling comparing tumors from non‐responders and responders after neoadjuvant chemoradiotherapy. (B,C) *LTβR* expression in paired and unpaired TCGA‐STAD tumor and normal samples. (D,E) Single‐cell RNA‐seq analysis showing *LTβR* expression across GC and normal gastric mucosal cells. (F) Correlation of *LTβR* expression with radiotherapy‐related gene sets ssGSEA score. (G,H) Representative clonogenic assays in AGS cells with *LTβR* knockdown and HGC‐27 cells with *LTβR* overexpression after the indicated doses of ionizing radiation (IR). (I,J) Survival fraction curves derived from clonogenic assays. (K–N) EdU staining and quantification of proliferative activity after IR. Scale bar, 50 µm. (O–R) Annexin V/PI flow cytometry and quantification of apoptosis in AGS cells (O, P) with *LTβR* knockdown and HGC‐27 cells (Q,R) with *LTβR* overexpression after the indicated doses of IR. Data are presented as mean ± SD. Statistical significance was determined by limma analysis (A), or paired Wilcoxon signed‐rank test (B), or unpaired Wilcoxon rank‐sum test (C), or two‐way ANOVA followed by Bonferroni's multiple comparisons test (I,J,K,L,P,R). Correlation analysis in (F) was performed using Pearson correlation analysis, *p < 0.05, **p < 0.01, ***p < 0.001.

To identify candidate determinants of radioresistance, we performed proteomic profiling of tumor tissues from 12 patients with GC who underwent surgery after neoadjuvant chemoradiotherapy. Based on TRG, patients were classified as non‐responders (n = 5) or responders (n = 7) (Table S1). Comparative analysis revealed a distinct set of differentially expressed proteins, including both upregulated and downregulated targets, in non‐responders relative to responders (Figure 1A). Among the proteins enriched in non‐responders were several factors previously linked to tumor radioresistance, including PINX1, CCL20, ATAD2, FBXO2, and KIFC1 [53, 54, 55, 56, 57], together with LTβR. Given our previous findings that LTβR contributes to GC progression and metastasis, whereas its role in radioresistance remains unknown, we selected LTβR for further investigation [50, 51].

Immunohistochemistry (IHC) analysis further confirmed that LTβR expression was higher in the non‐responder group than in the responder group (Figure S1A–C). Data from TCGA‐STAD showed that *LTβR* expression is higher in GC tissues than in normal gastric mucosa (Figure 1B,C). Consistently, analysis of public scRNA‐seq datasets showed that *LTβR* is expressed in GC epithelial cells and is elevated relative to normal gastric mucosal epithelial cells (Figure 1D,E). Pan‐cancer single‐cell datasets further indicated that *LTβR* expression is higher in tumor cells than in other cell types within the tumor microenvironment (Figure S1D). In the TCGA‐STAD cohort, patients were further stratified into *LTβR*‐high and *LTβR*‐low groups. GSEA based on Hallmark gene sets showed that high *LTβR* expression was significantly enriched in pathways associated with radioresistance, including DNA damage response, UV response, and reactive oxygen species metabolism (Figure S1E). To further examine the relationship between *LTβR* and radiotherapy response, we collected all radiotherapy‐related pathways annotated in Gene Ontology (Figure 1F). This analysis showed that high *LTβR* expression was associated with most of these pathways, further supporting a link between *LTβR* expression and radioresistance. Moreover, analysis of lung cancer cohorts from the Kaplan‐Meier Plotter database revealed that high LTβR expression predicted poor survival exclusively in patients receiving radiotherapy (Figure S1F), suggesting a conserved role in radioresistance across solid tumors.

For in vitro functional studies, AGS cells with relatively high *LTβR* expression and HGC‐27 cells with relatively low *LTβR* expression were selected for *LTβR* knockdown and overexpression experiments, respectively (Figure S1G–J). Colony formation assays showed that, under ionizing radiation (IR), *LTβR* knockdown markedly reduced the colony‐forming ability of AGS cells (Figure 1G). Survival fraction analysis further confirmed that *LTβR* knockdown significantly increased radiosensitivity compared with the negative control group (sh‐NC) (Figure 1I). In contrast, *LTβR* overexpression significantly enhanced the radioresistance of HGC‐27 cells (Figure 1H,J). EdU assays showed that *LTβR* knockdown significantly reduced the proportion of EdU‐positive cells after IR, indicating greater proliferative arrest (Figure 1K,M), whereas *LTβR* overexpression partially preserved proliferative activity under the same conditions (Figure 1L,N). Flow cytometry further showed that *LTβR* knockdown markedly increased IR‐induced apoptosis, whereas *LTβR* overexpression produced the opposite effect (Figure 1O–R). Together, these results demonstrate that *LTβR* promotes radioresistance in GC cells in vitro.

### 2.2 LTβR Promotes Radioresistance in GC Xenografts and Patient‐Derived Organoids

**FIGURE 2:**
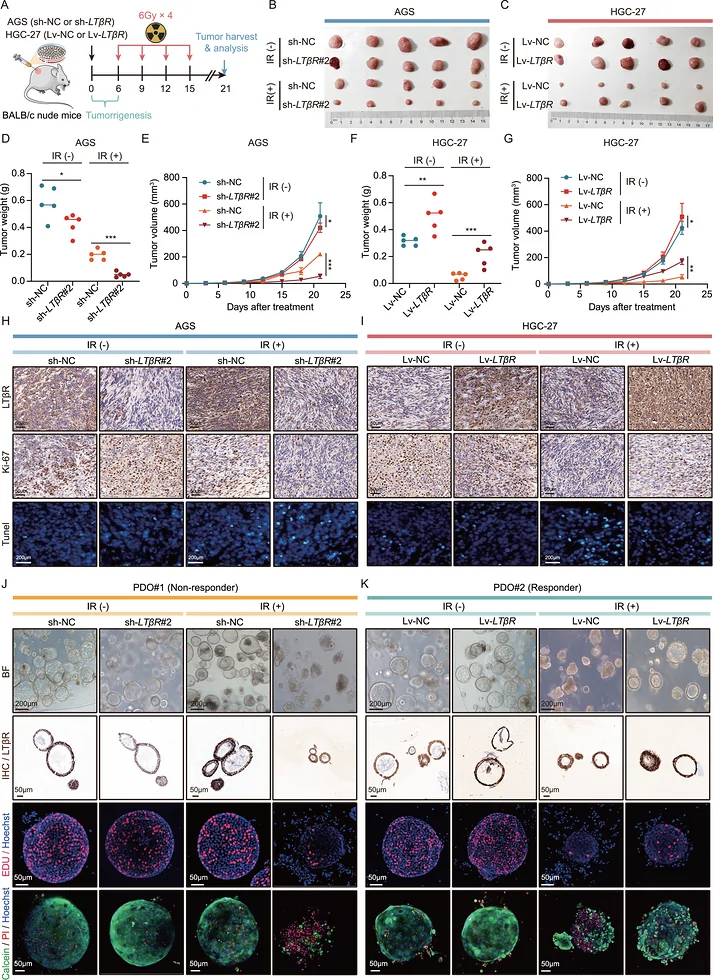
LTβR promotes radioresistance in GC xenografts and patient‐derived organoids. (A) Schematic of the subcutaneous xenograft experimental design. (B,C) Representative images of AGS (B) and HGC‐27 (C) xenograft tumors at the end of the experiment. (D–G) Tumor weight (D, F) and tumor growth curves (E,G) of AGS xenografts with *LTβR* knockdown (D, E) and HGC‐27 xenografts with *LTβR* overexpression (F,G), with or without ionizing radiation (IR, 6 Gy × 4). (H,I) Representative IHC and TUNEL staining in AGS and HGC‐27 xenografts. Scale bars: 50 µm (IHC), 200 µm (TUNEL). (J,K) Representative bright‐field, EdU/Hoechst, and Calcein‐AM/PI/Hoechst images of PDO#1 organoids with *LTβR* knockdown and PDO#2 organoids with *LTβR* overexpression, with or without 6 Gy IR in PDO#1 and PDO#2. Scale bars: 200 µm (bright‐field), 50 µm (IHC and fluorescence staining). Data are presented as mean ± SD. Statistical significance was determined by two‐way ANOVA followed by Bonferroni's multiple comparisons test (D–G), *p < 0.05, **p < 0.01, ***p < 0.001. integrated optical density: IOD, Immunohistochemical: IHC.

To further assess the role of LTβR in GC radioresistance, we generated subcutaneous xenograft models (Figure 2A). *LTβR* depletion inhibited tumor growth under both basal and irradiated conditions, and this effect was more pronounced after IR, indicating increased radiosensitivity (Figure 2B,D,E). Conversely, *LTβR* overexpression promoted tumor growth and diminished the response of GC xenografts to IR (Figure 2C,F,G). IHC analysis showed that *LTβR* depletion reduced Ki‐67 positivity after IR, whereas *LTβR* overexpression increased Ki‐67‐positive cells (Figure 2H; Figure S2A,B,D,E). Consistently, TUNEL staining showed that *LTβR* depletion enhanced IR‐induced apoptosis, while *LTβR* overexpression produced the opposite effect (Figure 2I; Figure S2C,F).

To further validate the role of LTβR in GC radioresistance, we established patient‐derived organoid models from five patients with GC. Among them, PDO#1 expressed *LTβR* at the highest level, whereas PDO#2 showed low *LTβR* expression (Figure S2G). Consistent with this pattern, PDO#1 was more resistant to irradiation than PDO#2 in cell viability assays (Figure S2H,I). We therefore silenced *LTβR* in PDO#1 and overexpressed *LTβR* in PDO#2. After IR, *LTβR*‐depleted PDO#1 organoids exhibited marked structural disruption, reduced proliferation, and increased cell death compared with control organoids (Figure 2J, Figure S2J–M). In contrast, *LTβR* overexpression in PDO#2 organoids preserved structural integrity, maintained proliferation, and reduced cell death after IR (Figure 2K, Figure S2N–Q). Together, these findings further support a role for LTβR in promoting radioresistance in GC and identify LTβR as a potential therapeutic target for overcoming radioresistance.

### 2.3 LTβR Suppresses IR‐Induced Pyroptosis Through the NLRP3/Caspase‐1/GSDMD Pathway

**FIGURE 3:**
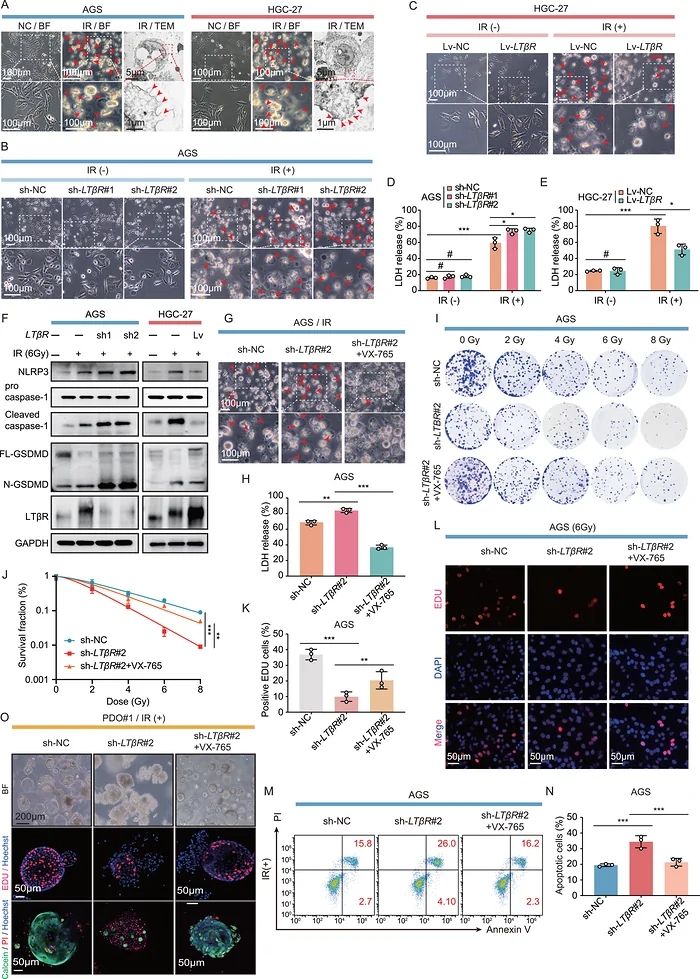
LTβR suppresses IR‐induced pyroptosis through the NLRP3/caspase‐1/GSDMD pathway. (A) Representative bright‐field and TEM images showing pyroptotic morphology and membrane pore formation after IR. Scale bars: 100 µm (bright‐field), 5 µm (TEM), 1 µm (magnified TEM). (B,C) Representative images for morphological assessment of pyroptosis in AGS cells with *LTβR* knockdown (B) and HGC‐27 cells with *LTβR* overexpression (C), with or without 6 Gy IR. Scale bar, 100 µm. (D,E) Quantification of LDH release to measure pyroptotic cell death in the above groups. (F) Western blot analysis of NLRP3, pro‐caspase‐1 and cleaved caspase‐1, FL‐GSDMD, and N‐GSDMD in AGS cells with *LTβR* knockdown and HGC‐27 cells with *LTβR* overexpression, with or without 6 Gy IR. (G–N) Rescue experiments in AGS cells with *LTβR* knockdown treated with or without the Caspase‐1‐specific inhibitor VX‐765 (500 nM, 2 h) after 6 Gy IR, including bright‐field imaging, LDH release, clonogenic survival, EdU staining, and Annexin V/PI apoptosis analysis. Scale bars: 100 µm (bright‐field), 50 µm (EdU fluorescence staining). (O) Representative bright‐field, EdU/Hoechst, and Calcein‐AM/PI/Hoechst images of PDO#1 organoids with *LTβR* knockdown treated with or without VX‐765 (500 nM, 2 h) after 6 Gy IR. Scale bars: 200 µm (bright‐field), 50 µm (fluorescence staining). Data are presented as mean ± SD. Statistical significance was determined by two‐way ANOVA followed by Bonferroni's multiple comparisons test (D,E,J), or one‐way ANOVA with Tukey post‐test (H,K,N), *p < 0.05, **p < 0.01, ***p < 0.001, #*p* > 0.05. Transmission electron microscopy: TEM.

During irradiation of GC cells, we observed prominent cell swelling and extensive membrane blebbing. Transmission electron microscopy further showed pore formation in the plasma membrane, consistent with pyroptotic cell death (Figure 3A) [58]. *LTβR* depletion enhanced IR‐induced pyroptosis, whereas *LTβR* overexpression attenuated this response (Figure 3B,C). In line with these findings, *LTβR* depletion increased IR‐induced lactate dehydrogenase (LDH), IL‐1β and IL‐18 release, while *LTβR* overexpression reduced it (Figure 3D,E; Figure S3B–E).

Previous studies have shown that IR can induce pyroptosis through either the caspase‐3/GSDME pathway or the NLRP3/caspase‐1/GSDMD pathway [12, 14]. To determine which pathway is regulated by LTβR, we examined the expression of key proteins involved in pyroptosis. LTβR expression had no detectable effect on caspase‐3 cleavage or on levels of N‐GSDME (Figure S3A). In contrast, *LTβR* knockdown markedly enhanced IR‐induced NLRP3 expression, caspase‐1 cleavage, and the abundance of the GSDMD N‐terminal fragment, whereas *LTβR* overexpression inhibited this pathway (Figure 3F).

To determine whether LTβR suppresses pyroptosis through the NLRP3/caspase‐1/GSDMD pathway, we treated cells with the caspase‐1 inhibitor VX‐765 [59]. In AGS cells, VX‐765 markedly attenuated the increase in LDH, IL‐1β and IL‐18 release induced by *LTβR* knockdown under IR (Figure 3G,H; Figure S3F,G). Clonogenic and EdU assays showed that VX‐765 partially rescued the impaired colony formation and proliferation of sh‐*LTβR* cells after irradiation (Figure 3I–L). VX‐765 also reduced IR‐induced apoptosis in sh‐*LTβR* cells (Figure 3M,N). Consistent results were obtained in organoids derived from a non‐responder with high LTβR expression, where VX‐765 partially reversed the structural disruption, reduced proliferation, and increased cell death caused by *LTβR* knockdown under IR (Figure 3O; Figure S3H–J). These data indicate that suppression of NLRP3/caspase‐1/GSDMD‐mediated pyroptosis contributes to LTβR‐driven radioresistance in GC.

### 2.4 LTβR Suppresses Pyroptosis by Enhancing the Translational Efficiency of SARM1

**FIGURE 4:**
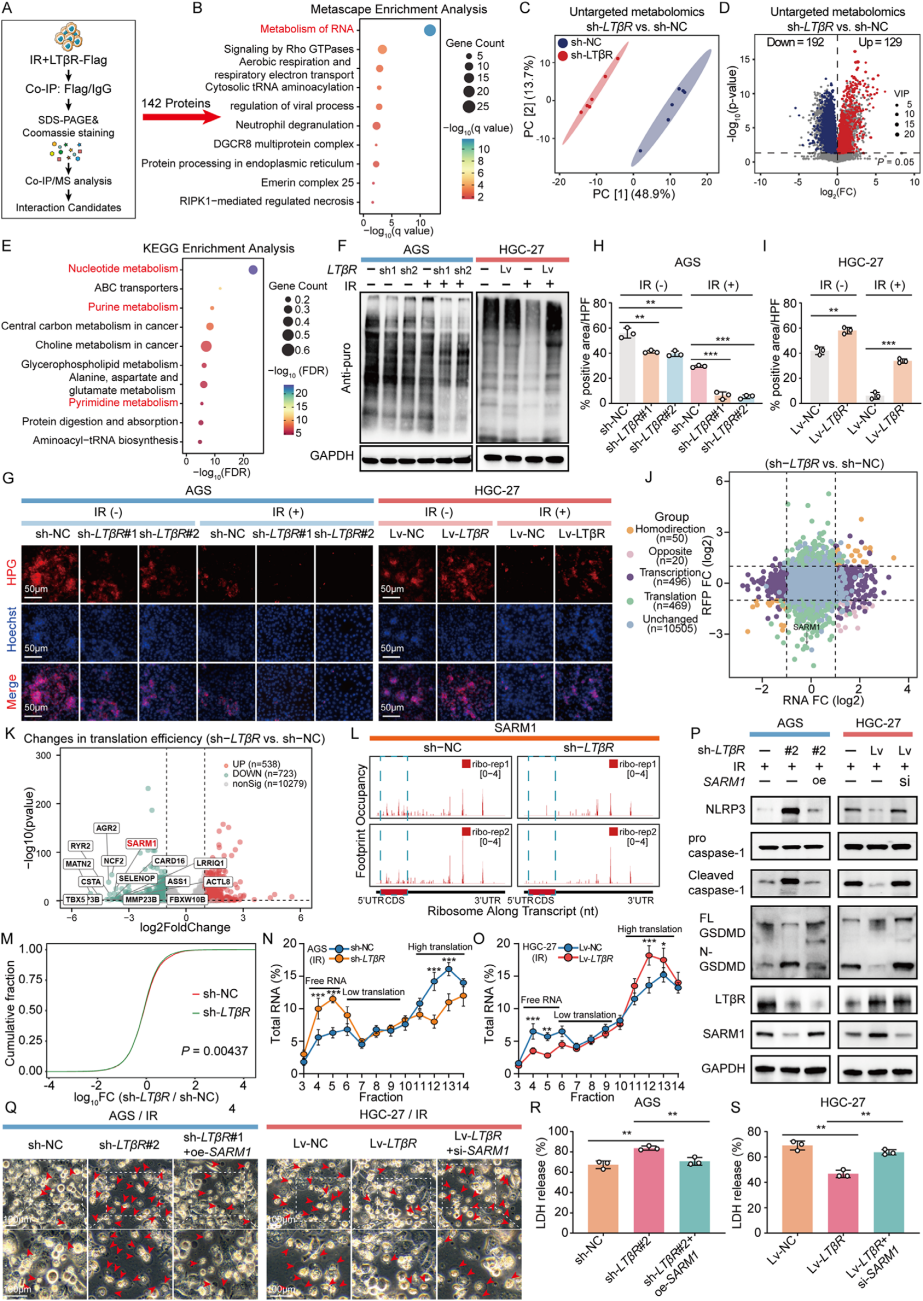
LTβR suppresses pyroptosis by enhancing the translational efficiency of SARM1. (A) Workflow of immunoprecipitation‐mass spectrometry analysis used to identify LTβR‐interacting proteins after IR. (B) Metascape enrichment analysis of LTβR‐associated proteins. (C–E) Untargeted metabolomics analyses, including PCA, differential metabolite profiling, and KEGG enrichment. (F–I) SUnSET and HPG assays showing global protein synthesis after *LTβR* knockdown or overexpression under IR conditions. Scale bar, 50 µm. (J,K) Integrated RNA‐seq and Ribo‐seq analyses identifying genes with altered translational efficiency, with *SARM1* highlighted as a candidate downstream effector. (L) Ribosome footprint occupancy across the *SARM1* transcript. (M) Cumulative distribution analysis of translational efficiency changes. (N,O) Polysome profiling showing redistribution of *SARM1* mRNA across translational fractions after *LTβR* modulation. (P–S) Functional rescue experiments with *SARM1* overexpression or silencing, including morphology, LDH release, and Western blot analysis of pyroptosis‐related proteins. Scale bar, 100 µm. Data are presented as mean ± SD. Statistical significance was determined by Student's t‐test (M), or two‐way ANOVA followed by Bonferroni's multiple comparisons test (H,I,N,O), or one‐way ANOVA with Tukey post‐test (R,S), *p < 0.05, **p < 0.01, ***p < 0.001.

To explore the mechanism by which LTβR regulates pyroptosis via NLRP3/caspase‐1/GSDMD, we performed immunoprecipitation‐mass spectrometry (IP‐MS) in Flag‐*LTβR*‐transfected HGC‐27 cells after irradiation (Figure 4A). This analysis identified 142 candidate LTβR‐interacting proteins, with significant enrichment in RNA metabolism‐related pathways (Figure 4B; Table S2). Untargeted metabolomics analysis revealed concordant changes, further supporting a role for RNA metabolic regulation in LTβR‐mediated pyroptosis (Figure 4C–E; Table S3).

Because RNA metabolism is closely linked to protein translation, we next examined global translational activity [60]. SUnSET and HPG assays showed that *LTβR* knockdown markedly reduced protein synthesis under IR, whereas *LTβR* overexpression produced the opposite effect (Figure 4F–I). To identify translational targets downstream of LTβR, we performed RNA‐seq and Ribo‐seq analyses. Ribo‐seq quality metrics were consistent across samples and supported the reliability of the datasets, with the expected RPF size distribution, predominant mapping to coding sequences, and clear 3‐nt periodicity (Figure S4A–C). *LTβR* knockdown did not substantially alter the distribution of RPF reads across transcript regions (Figure S4D). Principal component analysis (PCA) showed separation between groups in both datasets (Figure S4E,F). In sh‐*LTβR* cells, Ribo‐seq identified 299 upregulated and 324 downregulated genes, whereas RNA‐seq identified 472 upregulated and 298 downregulated genes, relative to sh‐NC cells (Figure S4G,H).

Functional enrichment analyses consistently showed suppression of translation‐related programs in sh‐*LTβR* cells. RNA‐seq‐based GO and GSEA revealed downregulation of ribosome biogenesis, rRNA metabolism, rRNA processing, and ribonucleoprotein complex biogenesis, along with inhibition of irradiation‐related programs including DNA replication and DNA‐templated DNA replication (Figure S5A). KEGG and Hallmark analyses further showed reduced enrichment of ribosome biogenesis in eukaryotes, GC, microRNAs in cancer, MYC TARGETS V1, MYC TARGETS V2, MTORC1 SIGNALING, and UV RESPONSE DN (Figure S5B,C). Ribo‐seq analyses showed a similar pattern, with KEGG and Hallmark GSEA confirming suppression of multiple translation‐associated pathways, including MYC TARGETS V1 and MTORC1 SIGNALING, in sh‐*LTβR* cells (Figure S5D,E). These findings support a role for LTβR in maintaining translation‐related programs.

To identify translational events involved in LTβR‐dependent suppression of pyroptosis under irradiation, we integrated the RNA‐seq and Ribo‐seq datasets and examined genes with altered translational efficiency. Among the top five genes with reduced translational efficiency after *LTβR* knockdown, *SARM1* was of particular interest because it is the only candidate reported to negatively regulate the NLRP3/caspase‐1 pathway, in line with the mechanism by which LTβR suppresses pyroptosis (Figure 4J,K). Ribo‐seq analysis showed that *LTβR* depletion markedly reduced ribosome occupancy across the coding sequence of *SARM1* (Figure 4L). Consistent with this finding, translational efficiency was decreased in sh‐*LTβR* cells (Figure 4M). *LTβR* overexpression increased SARM1 protein expression without affecting *SARM1* mRNA abundance, whereas LTβR knockdown reduced SARM1 protein levels with no significant change in mRNA expression (Figure S5F–H). Polysome profiling further showed that *LTβR* knockdown shifted *SARM1* mRNA from heavy polysome fractions to lighter fractions, whereas *LTβR* overexpression promoted its association with actively translating polysomes (Figure 4N,O). These data indicate that LTβR regulates *SARM1* primarily at the translational level.

Functional rescue experiments further supported this model. Under irradiation, overexpression of *SARM1* in *LTβR*‐depleted cells partially reversed the pyroptotic phenotype, as shown by reduced activation of the NLRP3/caspase‐1/GSDMD pathway and attenuated GSDMD cleavage, along with decreased LDH, IL‐1β and IL‐18 release (Figure 4P–R; Figures S5I and S6A,B). In contrast, *SARM1* knockdown enhanced pyroptosis in *LTβR*‐overexpressing cells (Figure 4P–S; Figures S5I,J and S6A–D). These results indicate that LTβR suppresses IR‐induced pyroptosis, at least in part, by maintaining *SARM1* translation.

### 2.5 LTβR Enhances SARM1 Translation by Recruiting the RNA‐Binding Protein PCBP2

**FIGURE 5:**
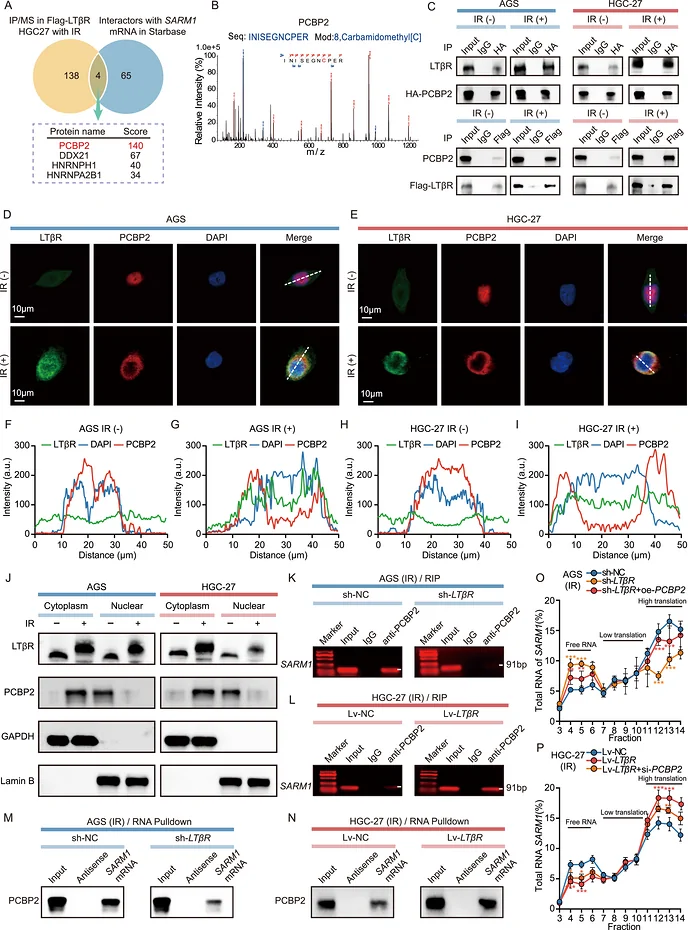
LTβR enhances *SARM1* translation by recruiting the RNA‐binding protein PCBP2. (A) Overlap analysis between LTβR‐interacting proteins identified by IP‐MS and predicted *SARM1*‐binding RNA‐binding proteins. (B) Representative mass spectrum identifying PCBP2 in the LTβR interactome. (C) Co‐immunoprecipitation assays validating the interaction between LTβR and PCBP2 in AGS and HGC‐27 cells with or without IR. (D–I) Immunofluorescence imaging and line‐scan intensity analyses showing LTβR accumulation and PCBP2 redistribution after IR. Scale bar, 10 µm. (J) Cytoplasmic and nuclear fractionation assays for LTβR and PCBP2. (K,L) RIP assays showing LTβR‐dependent binding of PCBP2 to *SARM1* mRNA. (M,N) RNA pulldown assays confirming the interaction between PCBP2 and *SARM1* mRNA. (O,P) Polysome profiling showing that PCBP2 is required for LTβR‐mediated enhancement of *SARM1* translation. Data are presented as mean ± SD. Statistical significance was determined by two‐way ANOVA followed by Bonferroni's multiple comparisons test (O,P), *p < 0.05, **p < 0.01, ***p < 0.001.

To investigate the mechanism by which LTβR regulates *SARM1* translation, we first examined whether LTβR directly binds *SARM1* mRNA. RIP assays showed that LTβR does not directly associate with *SARM1* mRNA (Figure S7A). Given that LTβR is not an RNA‐binding protein, these findings suggest that the regulation of *SARM1* translation by LTβR requires intermediary factors. We therefore next sought to identify the molecules that connect LTβR to *SARM1* translation. To this end, LTβR‐interacting proteins identified by IP‐MS were intersected with predicted *SARM1*‐binding RBPs from the StarBase database. This analysis identified four overlapping candidates, among which PCBP2 was prioritized because it had the highest confidence score and is known to regulate translation (Figure 5A,B) [61].

Co‐IP assays showed that PCBP2 was enriched in LTβR immunocomplexes, and that this interaction was further enhanced after IR exposure (Figure 5C). Subcellular fractionation and immunofluorescence assays showed that IR increased the levels of LTβR in both the nucleus and the cytoplasm, whereas PCBP2 underwent marked translocation from the nucleus to the cytoplasm (Figure 5D–J). RIP and RNA pulldown assays further confirmed that PCBP2 binds SARM1 mRNA in an LTβR‐dependent manner (Figure 5K–N). To determine the functional significance of this interaction, we found that overexpression of *PCBP2* restored the reduced association of SARM1 mRNA with heavy polysome fractions in *LTβR*‐depleted cells (Figure 5O; Figure S7B). In contrast, silencing of *PCBP2* abolished the increase in *SARM1* polysome occupancy induced by *LTβR* overexpression (Figure 5P; Figure S7C). Together, these data indicate that PCBP2 is required for the LTβR‐dependent enhancement of *SARM1* translation.

### 2.6 LTβR Facilitates TRIM28‐mediated SUMOylation of PCBP2 and Its Cytoplasmic Translocation to Enhance SARM1 Translation

**FIGURE 6:**
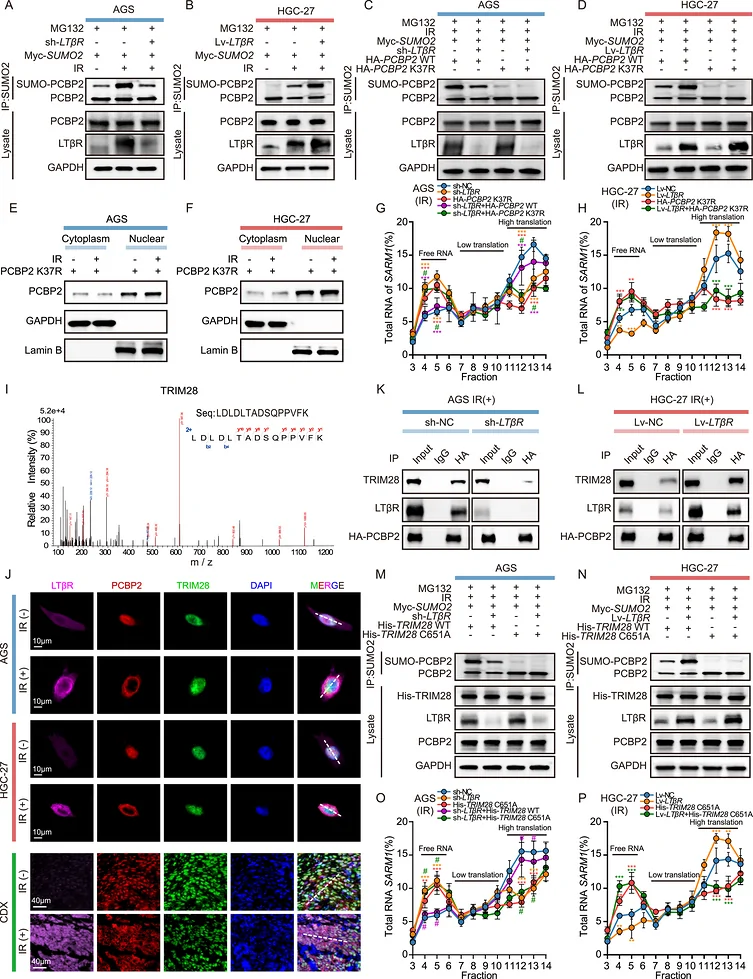
LTβR facilitates TRIM28‐mediated SUMOylation of PCBP2 and its cytoplasmic translocation to enhance SARM1 translation. (A,B) SUMOylation assays showing regulation of PCBP2 SUMOylation by LTβR under IR conditions. (C,D) Analysis of wild‐type PCBP2 and the K37R mutant demonstrating that K37 is required for LTβR‐dependent regulation of PCBP2 SUMOylation. (E,F) Cytoplasmic and nuclear fractionation showing that PCBP2 K37R fails to undergo IR‐induced cytoplasmic redistribution. (G,H) Polysome profiling showing that the K37R mutant abolishes LTβR‐mediated enhancement of *SARM1* translation. (I) Representative mass spectrum identifying TRIM28 in the LTβR interactome. (J) Immunofluorescence analysis showing nuclear colocalization of LTβR and TRIM28 after IR in AGS cells, HGC‐27 cells, and HGC‐27 subcutaneous CDX. Scale bars: 10 µm (cells), 40 µm (CDX). (K,L) Co‐immunoprecipitation assays showing that LTβR promotes the interaction between PCBP2 and TRIM28. (M,N) SUMOylation assays using wild‐type TRIM28 and the SUMO E3 ligase‐deficient mutant C651A. (O,P) Polysome profiling showing that TRIM28 enzymatic activity is required for LTβR‐dependent translation of *SARM1*. Data are presented as mean ± SD. Statistical significance was determined by two‐way ANOVA followed by Bonferroni's multiple comparisons test (G,H,O,P), *p < 0.05, **p < 0.01, ***p < 0.001, #*p* > 0.05. Cell‐derived xenograft: CDX.

Previous studies have shown that the nucleocytoplasmic shuttling of PCBP2 is regulated by SUMOylation at lysine 37 [62, 63]. SUMOylation assays showed that IR treatment increased the SUMOylation of PCBP2. This effect was significantly reduced by knockdown of *LTβR*, whereas overexpression of *LTβR* further enhanced the SUMOylation of PCBP2 (Figure 6A,B). We then generated a loss‐of‐function mutant at K37. Compared with wild‐type PCBP2, the K37R mutant abolished the regulatory effect of LTβR on the SUMOylation of PCBP2 under IR conditions (Figure 6C,D). Subcellular fractionation further showed that wild‐type PCBP2 underwent redistribution to the cytoplasm after IR, whereas the K37R mutant remained predominantly nuclear regardless of IR exposure (Figure 6E,F). Polysome profiling showed that wild‐type PCBP2, but not the K37R mutant, restored the association of *SARM1* mRNA with heavy polysome fractions in *LTβR*‐deficient cells (Figure 6G). Likewise, expression of the K37R mutant abolished the increase in *SARM1* translation induced by overexpression of *LTβR* (Figure 6H).

Because LTβR lacks intrinsic SUMO E3 ligase activity, we next asked whether it associates with a SUMO E3 ligase capable of modifying PCBP2. Reanalysis of the IP‐MS dataset of LTβR identified TRIM28, a canonical SUMO E3 ligase, as one of the core candidate proteins (Figure 6I) [64]. Immunofluorescence showed that TRIM28 was mainly localized in the nucleus, and subcellular fractionation confirmed that IR treatment markedly increased the abundance of LTβR in the nucleus (Figure 6J). Analysis of the Human Protein Atlas (HPA) subcellular localization data showed that LTβR was also present in the nucleus (Figure S8A). Under IR conditions, LTβR and TRIM28 clearly colocalized in the nucleus (Figure 6J; Figure S8B–G). However, LTβR does not contain a nuclear localization sequence, which raises the question of how it enters the nucleus. We therefore tested the possibility that nuclear import of LTβR depends on TRIM28. Consistent with this model, *TRIM28* knockdown markedly reduces the nuclear localization of LTβR (Figure S8H–O). Furthermore, treatment with the nuclear export inhibitor leptomycin B (LMB) increased the nuclear accumulation of LTβR, indicating regulated nucleocytoplasmic trafficking of LTβR. (Figure S9A–F). Nuclear Co‐IP and protein–protein docking analyses supported the formation of an LTβR–TRIM28–PCBP2 nuclear complex, and this association was enhanced after irradiation (Figure S9G,H). Co‐IP assays further showed that *LTβR* depletion weakened the interaction between PCBP2 and TRIM28, whereas *LTβR* overexpression strengthened it (Figure 6K,L). To determine whether TRIM28 is required for the SUMOylation of PCBP2, we generated a TRIM28 mutant, C651A, that lacks SUMO E3 ligase activity [65]. Unlike wild‐type TRIM28, the C651A mutant abolished the LTβR‐dependent regulation of the SUMOylation of PCBP2, and the SUMOylation of PCBP2 no longer responded to knockdown or overexpression of LTβR (Figure 6M,N).

Functional analyses showed that wild‐type TRIM28 restored the translational efficiency of SARM1 in *LTβR*‐deficient cells, whereas the C651A mutant failed to do so and also abolished the translational enhancement induced by overexpression of LTβR (Figure 6O,P). Analysis of the TCGA‐STAD dataset further showed that the expression levels of *TRIM28*, *PCBP2*, and *SARM1*, similar to *LTβR*, were closely associated with radiation‐response signatures such as “Response to Ionizing Radiation” (Figure S9I–K). These findings suggest that hyperactivation of the entire signaling axis is a hallmark of radioresistant GC. Together, these results indicate that LTβR promotes the SUMOylation and cytoplasmic localization of PCBP2 through TRIM28, thereby enabling efficient translation of *SARM1*.

### 2.7 EMD Reduces IR‐Induced Glycosylated LTβR Accumulation and Enhances Radiosensitivity

**FIGURE 7:**
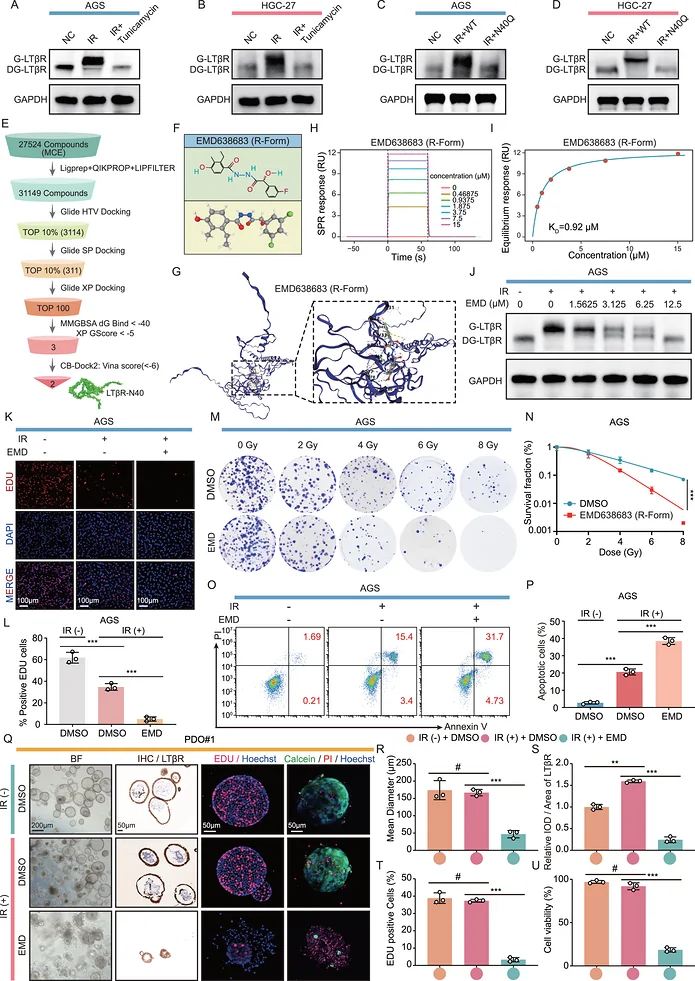
EMD reduces IR‐induced glycosylated LTβR accumulation and enhances radiosensitivity. (A,B) Western blot analysis of LTβR expression in AGS and HGC‐27 cells treated with or without tunicamycin (2 µg/mL, 24 h) and 6 Gy IR. (C,D) Western blot analysis of wild‐type LTβR and N40Q mutant LTβR expression in AGS and HGC‐27 cells with or without 6 Gy IR. (E) Workflow of structure‐based virtual screening for compounds targeting the N40 region of LTβR. (F) Chemical structure and 3D structure of EMD638683. (G) Docking model of EMD638683 binding to the N40 region of LTβR. (H, I) Surface plasmon resonance analysis of EMD binding to LTβR at indicated concentrations. (J) Western blot analysis of LTβR expression in AGS cells treated with 0–12.5 µM EMD after 6 Gy IR. (K–P) Functional assays in AGS cells treated with DMSO or 12.5 µM EMD with or without 6 Gy IR, including clonogenic survival, EdU staining, and Annexin V/PI apoptosis analysis. Scale bars: 100 µm. (Q–U) Representative images and quantitative analyses of PDO#1 organoids treated as indicated. Assays include bright‐field imaging, IHC staining for LTβR, EdU/Hoechst staining, and Calcein‐AM/PI/Hoechst staining. Scale bars: 200 µm (bright‐field), 50 µm (IHC and fluorescence staining). Data are presented as mean ± SD. Statistical significance was determined by two‐way ANOVA followed by Bonferroni's multiple comparisons test (N), or one‐way ANOVA with Tukey post‐test (L,P,R–U), *p < 0.05, **p < 0.01, ***p < 0.001, #*p* > 0.05.

Consistent with the previous western blot results, IR not only increased the protein expression of LTβR but also caused an upward shift in its apparent molecular weight. Because LTβR is a well‐characterized N‐glycosylated protein [66], we hypothesized that this mobility shift reflects enhanced glycosylation. Treatment with tunicamycin largely abolished the high‐molecular‐weight band of LTβR induced by IR, indicating that IR promotes the N‐glycosylation of LTβR (Figure 7A,B).

Previous studies have shown that N40 is a glycosylation site of LTβR and that this modification stabilizes LTβR by reducing its ubiquitination [66]. In contrast with wild‐type LTβR, the N40Q mutant failed to show the IR‐induced mobility shift and accumulated at much lower levels after IR treatment (Figure 7C,D). Ubiquitination assays further showed that this reduction was associated with increased ubiquitination of the N40Q mutant (Figure S10A,B).

Because glycosylation at N40 is required for the stability of LTβR, we next performed structure‐based virtual screening to identify small molecules targeting the N40 region. A total of 27 524 compounds from the MCE database were screened, which yielded two candidate molecules, EMD and UNBS5162 (Figure 7E–G; Figure S10C,D). Surface plasmon resonance (SPR) analysis showed that EMD bound LTβR with higher affinity than UNBS5162, with dose‐dependent binding kinetics and an equilibrium dissociation constant of 0.92 µM (Figure 7H,I; Figure S10E,F). Furthermore, CETSA assay confirmed the stable intracellular binding between EMD and LTβR (Figure S10G,H). GO analysis based on the CTD further showed that the targets of EMD were enriched in pathways related to radiation response and programmed cell death (Figure S10I). Consistent with these findings, treatment with EMD reduced the accumulation of glycosylated LTβR in a dose‐dependent manner after IR in GC cells (Figure 7J).

EdU and clonogenic survival assays showed that EMD markedly enhanced IR‐induced growth inhibition and radiosensitivity in AGS cells (Figure 7K–N). Annexin V/PI flow cytometry further confirmed that EMD increased IR‐induced apoptosis (Figure 7O,P). These findings were further validated in patient‐derived organoid models, in which EMD effectively reversed radioresistance in organoids derived from PDO#1, who was a non‐responder, as reflected by pronounced structural disruption, reduced proliferative activity, and increased cell death (Figure 7Q–U). To assess whether the radiosensitizing effect of EMD depends on LTβR, we evaluated its efficacy in *LTβR*‐depleted cells. While EMD markedly inhibited colony formation and promoted apoptosis in control cells under irradiation, it failed to exert additional effects in *LTβR*‐knockdown cells (Figure S10J–M). Additionally, while EMD is a known SGK1 inhibitor [67], it maintained its strong radiosensitizing effect even when *SGK1* was depleted (Figure S11A–F). These data support LTβR as a major functional target contributing to the radiosensitizing effect of EMD.

### 2.8 CRGD‐Lipo@EMD Enhances the Radiosensitivity of GC In Vivo

**FIGURE 8:**
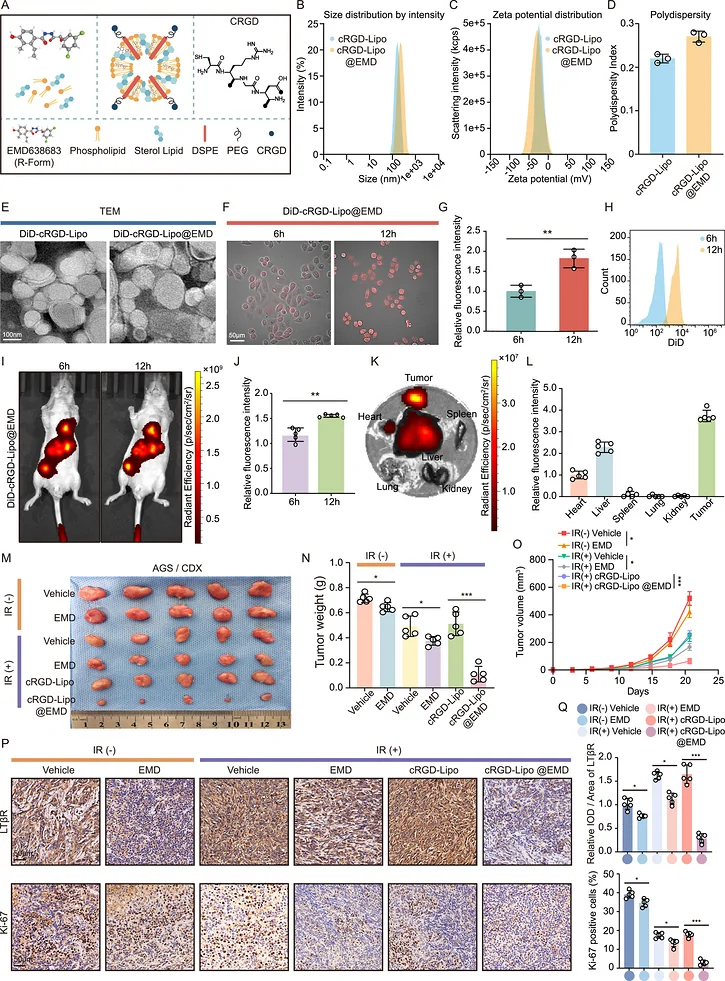
cRGD‐Lipo@EMD enhances the radiosensitivity of GC in vivo. (A) Schematic of cRGD‐conjugated EMD‐loaded liposomes. (B–D) Physicochemical characterization of cRGD‐Lipo and cRGD‐Lipo@EMD, including particle size distribution, polydispersity index, and zeta potential. (E) TEM images of DiD‐cRGD‐Lipo and DiD‐cRGD‐Lipo@EMD. Scale bar, 100 nm. (F–H) Cellular uptake of fluorescently labeled liposomes in AGS cells by fluorescence imaging and flow cytometry. Scale bar, 50 µm. (I,J) In vivo IVIS imaging and quantification of tumor fluorescence after intravenous injection of DiD‐cRGD‐Lipo@EMD. (K,L) Ex vivo fluorescence imaging and quantitative biodistribution analysis in tumors and major organs. (M–O) Therapeutic efficacy assays in AGS xenografts, including tumor growth curves, representative tumor images, and tumor weights. (P,Q) Immunohistochemical staining and quantification of LTβR and Ki‐67 in excised tumors. Scale bar, 50 µm. Data are presented as mean ± SD. Statistical significance was determined by Student's *t*‐test (G,J), or two‐way ANOVA followed by Bonferroni's multiple comparisons test (N,O,Q), *p < 0.05, **p < 0.01, ***p < 0.001.

To enhance tumor‐targeted delivery of EMD, PEGylated liposomes were modified with cyclic arginine‐glycine‐aspartic acid (cRGD) peptides. The structural design of cRGD‐conjugated, EMD‐encapsulated liposomes (designated cRGD‐Lipo@EMD) is presented (Figure 8A). These liposomes exhibited an encapsulation efficiency of 80.25% and a drug loading capacity of 7.56%. The resulting liposomes show a uniform size distribution and high encapsulation efficiency (Figure 8B–D). Transmission electron microscopy further shows a typical lipid droplet‐like morphology (Figure 8E). Compared with free EMD, cRGD‐Lipo@EMD showed a more sustained release profile over 48 h, with approximately 75% of the payload released at 12 h (Figure S12A). In addition, after incubation in PBS containing 10% FBS at 37°C for 48 h, cRGD‐Lipo@EMD displayed only slight changes in particle size and PDI (Figure S12B). Fluorescence imaging and flow cytometry confirm efficient uptake of cRGD‐Lipo@EMD by AGS cells (Figure 8F–H).

We next evaluate tumor‐targeting capacity in vivo by intravenous injection of DiD‐cRGD‐Lipo@EMD into AGS tumor‐bearing mice. Real‐time IVIS imaging shows gradual accumulation of fluorescence in the tumor region over 12 h (Figure 8I,J). Ex vivo imaging showed tumor accumulation of DiD‐cRGD‐Lipo@EMD (Figure 8K,L).

We next evaluated the in vivo biosafety of free EMD and cRGD‐Lipo@EMD. Neither treatment altered mouse body weight over the course of the experiment (Figure S12C). H&E staining of major organs showed no evident pathological abnormalities, suggesting that the treatment did not cause overt systemic toxicity (Figure S12D). However, serum biochemical analyses revealed mild elevations in alanine aminotransferase (ALT, a marker of liver function) and urea (a marker of kidney function) in the free EMD‐treated group, whereas these parameters remained within normal limits in the cRGD‐Lipo@EMD‐treated group (Figure S12E,F). These findings reveal that cRGD‐Lipo@EMD exhibits favorable systemic biosafety with no evident organ toxicity, while free EMD causes only mild biochemical changes without corresponding histopathological damage.

The combination of cRGD‐Lipo@EMD and IR showed the strongest synergistic effects, significantly reducing cell survival and increasing apoptosis in vitro (Figure S12G–J), while suppressing tumor volume and weight in vivo (Figure 8M–O and Figure S12K). Immunohistochemical analysis of excised tumors further shows that this combination treatment causes the most pronounced reduction in the expression of LTβR and Ki‐67 (Figure 8P,Q). Together, these results identify cRGD‐Lipo@EMD as an effective tumor‐targeted radiosensitizer for GC.

## 3 Discussion

Our study identifies LTβR as a clinically relevant determinant of radioresistance in GC and extends its role beyond immune organization to a tumor cell‐intrinsic stress adaptation program. Using patient specimens, xenografts, and patient‐derived organoids, we found that LTβR is preferentially associated with radioresistant disease and supports tumor cell survival after irradiation. These findings address an unresolved question in the field, namely whether aberrantly overexpressed immune‐related molecules can influence the response of tumor cells to radiotherapy rather than acting only through the microenvironment [68, 69, 70]. More broadly, our data support a view of radioresistance in GC that is not limited to DNA damage repair and evasion of apoptosis, but also includes adaptive suppression of pyroptosis as an important contributor to treatment failure [12, 71, 72].

One of the main findings of this study is that LTβR suppresses irradiation‐induced pyroptosis through the NLRP3/caspase‐1/GSDMD axis. Pyroptosis is increasingly recognized as an important component of radiation‐induced tumor cell injury, but the upstream mechanisms that determine whether this pathway is engaged in irradiated cancer cells are still not well defined [73, 74]. Our data place LTβR upstream of this process and suggest that radioresistant tumor cells do not simply endure radiation damage, but actively limit inflammatory lytic cell death. This is important both mechanistically and clinically, because pyroptosis is not only cytotoxic but may also be immunogenic and capable of reshaping the local inflammatory environment after treatment [75]. In this setting, LTβR appears to act as a restraint on a death program that would otherwise improve radiation response. These findings add to current models of resistance by indicating that avoidance of pyroptosis may matter as much as DNA repair or escape from apoptosis during radiotherapy‐induced stress [76, 77, 78].

Our results also define a noncanonical LTβR signaling program centered on translational control. We found that irradiation increases the N‐glycosylation of LTβR, which is accompanied by enhanced nuclear accumulation of the receptor. A similar phenomenon has been reported for PD‐L1, whose glycosylated forms are increased after irradiation and whose nuclear localization has been implicated in DNA damage repair, angiogenesis, and immune evasion [79, 80, 81, 82, 83]. In this context, our findings broaden the current understanding of immune‐related genes aberrantly expressed by tumor cells. Rather than acting primarily through classical membrane‐proximal inflammatory signaling cascades, LTβR sustains *SARM1* translation, thereby suppressing pyroptotic signaling after irradiation. This observation links cellular adaptation to radiotherapy with selective translational reprogramming, an understudied aspect of tumor stress biology that has gained increasing attention yet remains incompletely understood [60, 84]. The finding that LTβR regulates SARM1 at the translational level rather than through changes in transcript abundance indicates that therapy‐resistant cells can rapidly modulate their susceptibility to cell death without requiring extensive transcriptional rewiring. In contrast, a recent study by Wang et al. reported that SARM1 senses cytosolic double‐stranded DNA and triggers metabolic cell death through its NADase activity, suggesting that DNA damage would promote cytotoxicity [85]. However, in our GC models, the LTβR‐SARM1 axis markedly promoted cellular survival. During Klebsiella pneumoniae infection, SARM1 is strongly induced through the TLR4‐TRAM‐TRIF‐IRF3‐IFNAR1 pathway and functions as a broad negative regulator of host antibacterial immunity. Specifically, SARM1 suppresses MyD88‐ and TRIF‐dependent inflammatory signaling cascades, inhibits ERK and JNK MAPK activation, reduces the release of pro‐inflammatory cytokines, restricts AIM2 inflammasome activation and IL‐1β production, and fine‐tunes the p38‐type I interferon axis to promote IL‐10 expression, thereby further constraining host defense responses [86]. Consistent with this immunosuppressive function, a study by Carty et al. demonstrated that SARM1 directly inhibits the NLRP3 inflammasome, reduces IL‐1β release, and promotes pyroptosis in a mitochondrial depolarization‐dependent manner [87]. In the context of elevated LTβR expression, GC cells exhibit resistance to irradiation, and radioresistant cells display attenuated or even abrogated mitochondrial depolarization after irradiation [88]. These data suggest that, in this specific context, SARM1 primarily suppresses pyroptosis by inhibiting inflammasome activation, thereby contributing to cellular survival rather than cell death. Our findings thus identify translational efficiency, rather than mRNA abundance alone, as a critical determinant of pyroptotic competence in irradiated GC cells.

The LTβR‐TRIM28‐PCBP2‐SARM1 axis further links SUMOylation to pyroptosis. SUMOylation is already known to regulate DNA damage response, chromatin homeostasis, and stress signaling, but its role in the control of pyroptosis during radiotherapy has remained unclear [89]. Our data suggest that SUMOylation affects pyroptosis indirectly by controlling the subcellular trafficking and translational activity of PCBP2. In this model, irradiation promotes a noncanonical nuclear function of LTβR, allowing LTβR to facilitate TRIM28‐dependent SUMOylation of PCBP2, promote cytoplasmic redistribution of PCBP2, and increase loading of SARM1 mRNA onto actively translating polysomes. This mechanism broadens current understanding of how post‐translational modification shapes treatment response. SUMOylation may influence pyroptotic output not only through direct effects on inflammasome components or gasdermins, but also through regulation of RNA‐binding proteins and selective mRNA translation [63, 65, 90]. Taken together, these findings place receptor signaling, post‐translational modification, and translational regulation in a single adaptive axis that supports radioresistance.

These results also shed light on the context‐dependent role of LTβR in cancer. Previous preclinical work has shown that LTβR activation enhances antitumor immunity, yet LTβR agonists have yielded limited clinical activity (ClinicalTrials: NCT06448364). Our findings suggest that this disconnect between preclinical promise and clinical outcomes reflects compartment‐specific biology rather than an inherent contradiction. LTβR signaling in immune and stromal compartments drives immune activation, while tumor cell‐intrinsic LTβR instead promotes cell survival under radiotherapy‐induced stress. This distinction is particularly relevant in GC, where treatment response depends not only on tumor cell genotype, but also on dynamic crosstalk between inflammatory signaling, cell death programs, and tumor microenvironment remodeling [91, 92]. From a translational perspective, these findings position LTβR not only as an immunotherapeutic target, but also as a biomarker‐defined, tumor‐intrinsic vulnerability in the setting of radiotherapy.

The therapeutic significance of our findings is underscored by the identification of EMD as a compound that reduces glycosylated LTβR accumulation and targets LTβR‐associated radiosensitization. These results demonstrate that LTβR‐driven radioresistance depends not only on downstream pathway activation but also on glycosylation‐mediated receptor stability, revealing a previously unrecognized point of intervention for disrupting therapy‐resistant phenotypes. Rather than escalating radiation doses empirically to overcome resistance, targeting LTβR offers a mechanistically grounded strategy that selectively dampens adaptive survival signaling in resistant tumors. Nanotechnology enables precise targeting of malignant cells with minimal collateral damage to normal tissues [93], and nanoparticle‐based delivery systems have proven transformative for cancer treatment [94]. The liposomal formulation employed here enhances tumor‐specific drug delivery and achieves effective radiosensitization in vivo, strengthening the translational outlook of this approach. Clinically, these findings support stratifying GC patients with elevated LTβR expression for radiosensitizing interventions aimed at disrupting LTβR stability, accumulation, or downstream signaling function.

Our study has several important limitations. First, the clinical correlative analyses were conducted in a single‐center cohort with a limited sample size and included only patients with adenocarcinoma of the gastroesophageal junction. This limitation should be addressed in larger, multicenter cohorts with comprehensive clinical annotation, particularly to determine whether specific genomic or inflammatory contexts confer greater dependence on LTβR signaling. Second, although we found that LTβR can accumulate in the nucleus, whether nuclear LTβR has direct chromatin‐associated or transcriptional regulatory activity remains unknown and warrants further investigation. Third, although nuclear Co‐IP and docking analyses support the formation of an LTβR–TRIM28–PCBP2 nuclear complex, the precise interaction domains between LTβR and TRIM28 remain unmapped. Fourth, whether the anti‐pyroptotic function of SARM1 in irradiated GC cells depends on its canonical NADase activity or on non‐enzymatic scaffolding functions remains unresolved. Fifth, our findings provide only preclinical proof‐of‐concept evidence for the feasibility of EMD delivery and its ability to enhance radiotherapy response in GC models. We do not yet have sufficiently comprehensive safety data to support formal clinical translation of this strategy.

**FIGURE 9:**
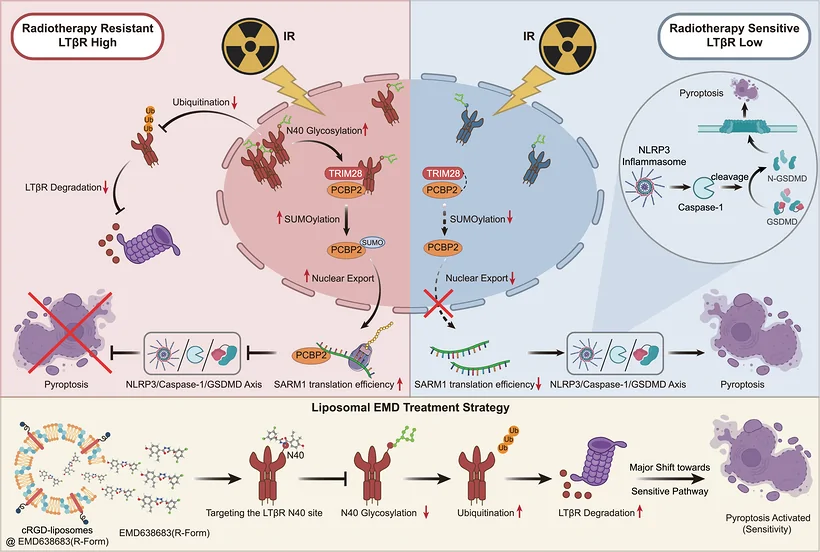
Proposed model of LTβR‐mediated radioresistance in GC. Irradiation stabilizes LTβR in a glycosylation‐dependent manner in GC cells, enabling its accumulation in the nucleus. Nuclear LTβR enhances TRIM28‐mediated SUMOylation of PCBP2, facilitating PCBP2 export to the cytoplasm and increasing the translational efficiency of SARM1 mRNA. Upregulated SARM1 restrains irradiation‐induced pyroptosis through suppression of the NLRP3/caspase‐1/GSDMD axis, thereby promoting adaptive radioresistance.

Taken together, our study identifies a previously unrecognized non‐canonical LTβR signaling axis that drives adaptive radioresistance in GC by suppressing IR‐induced pyroptosis through coordinated translational control and SUMOylation‐dependent regulation (Figure 9). These findings deepen our understanding of the molecular mechanisms underlying GC radioresistance and the regulation of pyroptosis, and establish LTβR as a promising tumor‐targeted therapeutic strategy to improve radiotherapy outcomes for patients with GC.

## 4 Materials and Methods

### 4.1 Clinical Specimens

Between January 2023 and January 2024, tumor specimens were retrospectively collected from 12 patients with adenocarcinoma of the gastroesophageal junction who underwent surgical resection after preoperative neoadjuvant chemoradiotherapy. Resected tumor tissues were used for LC‐MS/MS‐based proteomic profiling and IHC analysis. Radiotherapy was delivered in strict adherence to ASTRO guidelines [5]. Treatment response was assessed using the Mandard tumor regression grade (TRG) system. Patients with TRG 1–2 were classified as responders, whereas patients with TRG 3–5 were classified as non‐responders.

### 4.2 Cell Culture, Transfection and Treatment

Human GC cell lines HGC‐27 (TCHu 22) and AGS (TCHu232) were purchased from the National Collection of Authenticated Cell Cultures (Shanghai, China). MKN‐28 (BNCC360329), Hs746.T (BNCC100957), MKN‐45 (BNCC337682), and GES‐1 (BNCC337969) were obtained from the BeNa Culture Collection (Henan, China). SNU‐216 cells (00216) were purchased from the Korean Cell Line Bank (Seoul, Republic of Korea). Cells were cultured in RPMI‐1640 medium (Corning, NY, USA) containing 10% fetal bovine serum (FBS; Procell, Wuhan, China) and 1% Penicillin‐Streptomycin (NCM Biotech, Suzhou, China) at 37°C with 5% CO_2_. LMB (HY‐16909) and EMD (HY‐15193A) were purchased from MedChemExpress (MCE, NJ, USA). Both agents were dissolved in dimethyl sulfoxide (DMSO, Sigma, MO, USA), and stored at −20°C until use. Lentiviral vectors for LTβR knockdown (sh‐*LTβR*: #1, GCTGCCAGCCGGGAATGTTCT; #2, GCACTGAAGCCGAGCTCAAAG; #3, GCCGGGCACTTCCAGAATACC) or overexpression (LV‐*LTβR*) and their controls were constructed by GeneChem (Shanghai, China); stable lines were selected using G418 (Servicebio, Wuhan, China). siRNAs targeting *SARM1* (si‐*SARM1*: #1, 5′‐ CAACGACAATACAGCATATGA‐3′; #2, 5′‐ CAGACCTAGGCATGAAATCAA‐3′; #3, 5′‐ CAGATGGAATGGTGCCCTCTA‐3′), *PCBP2* (si‐*PCBP2*: #1, 5′‐GGAATTGTCCTGAGAGAAT‐3′; #2, 5′‐ CACCUGAGGUGCUUCAUAA‐3′; #3, 5′‐CAGTGTGGCTCTCTTATTG‐3′), *TRIM28* (si‐*TRIM28*: #1, 5′‐ GCAUGUUCAAGCAAUUCAA‐3′; #2, 5′‐ GGAGAUGAUCCCUACUCAA‐3′; #3, 5′‐ GUGUGCAAGUGGAUGUCAA‐3′) and *SGK1* (si‐*SGK1*: #1, 5′‐ AAGUGUUCUAUGCAGUCAATT‐3′; #2, 5′‐ CCGCCAGCUGACAGGACAUTT‐3′; #3, 5′‐ CUGCAGAAGGACAGGACAA‐3′) were synthesized by Tsingke Biotech (Beijing, China). Expression plasmids for Flag‐*LTβR*, HA‐*PCBP2* (WT/K37R), and His‐*TRIM28* (WT/C651A) were obtained from GenePharma (Suzhou, China). Transfections were carried out using jetPRIME (Polyplus, Strasbourg, France) following the manufacturer's protocol. The detailed experimental methods were as previously described [95].

### 4.3 IR and Colony Formation, EdU Incorporation Assays

Colony formation assays were performed to assess radiosensitivity. AGS and HGC‐27 cells were seeded into 6‐well plates at densities ranging from 400–1000 cells, corresponding to IR doses of 0, 2, 4, 6, and 8 Gy, respectively. Following IR and a 2‐week incubation, colonies (>50 cells) were subjected to fixation with 4% paraformaldehyde, crystal violet staining, and subsequent enumeration. Survival fractions were calculated using the multi‐target single‐hit equation: SF = 1 – (1 – e^−D/D0^) ^N^ [96, 97]. For EdU proliferation assays, cells in 24‐well plates were subjected to transfection or EMD treatment followed by 6 Gy IR. After 24 h, DNA synthesis was evaluated using the Cell‐Light EdU Apollo567 Kit (RiboBio, Guangzhou, China) with a 2‐h EdU pulse (50 µM). Fluorescence images were captured using a THUNDER microscope (Leica, Wetzlar, Germany).

### 4.4 Apoptosis Analysis

After 48 h of IR, cells in each group were collected and incubated with apoptosis detection kit (fmsav647, FCMACS, Nanjing). The level of cell apoptosis was detected by Beckman Coulter flow cytometry. The relevant data were analyzed by flowjo software. The detailed experimental procedures were as previously described [98].

### 4.5 RNA Isolation and qRT‐PCR Analysis

Cellular total RNA was extracted utilizing the TRIzol reagent (Invitrogen, USA) and subsequently reverse‐transcribed into cDNA with the HiScript III first Strand cDNA Synthesis Kit (Vazyme, China) according to standard protocols. Gene expression levels were quantified via real‐time PCR using the Taq Pro Universal SYBR Mix (Vazyme). The specific primer sequences used were as follows: *LTβR* forward 5′‐GAAGGGTAACAACCACTGC‐3′ and reverse 5′‐CTTGGTTCTCACACCTGGT‐3′; *SARM1* forward 5′‐CCACGTGGGGCTTAGGTTAG‐3′ and reverse 5′‐ACAAGGGCAGTCTCCATTCC‐3′; *TRIM28* forward 5′‐ GCACTAGCTGTGAGGATAATG‐3′ and reverse 5′‐ AGTATGGTCCTTGGTGTACT‐3′; *PCBP2* forward 5′‐ CCCCAACCAGTGACCAAAGA‐3′ and reverse 5′‐ TGGGTCCAGCCAAAGTGATA ‐3′; *SGK1* forward 5′‐ GAGGATGGGTCTGAACGACT‐3′ and reverse 5′‐ GCATTCATAAGCTCAGGCTCC ‐3′; and *GAPDH* forward 5′‐GAGTCAACGGATTTGGTCGT‐3′ and reverse 5′‐TGGGTGGAATCATATTGGAA‐3′. All primers were obtained from Tsingke Biotech (Beijing, China).

### 4.6 Western Blotting

Cells were lysed using RIPA buffer (P0013B, Beyotime, Shanghai, China) containing protease inhibitors. Total protein extracts were separated by SDS‐PAGE, transferred to PVDF membranes, and incubated with primary antibodies overnight at 4°C after blocking. Antibodies against LTβR (20331‐1‐AP), NLRP3 (30109‐1‐AP), GSDME (13075‐1‐AP), SARM1 (28625‐1‐AP), PCBP2 (15070‐1‐AP), TRIM28 (15202‐1‐AP), γ‐H2AX (83307‐2‐RR), SGK1 (28454‐1‐AP) and GAPDH (60004‐1‐Ig) were purchased from Proteintech (Wuhan, China). Antibodies targeting Caspase‐1 p20 (PC10743S), Cleaved Caspase‐3 p17 (TA7022S), and GSDMD (PU224937S) were obtained from Abmart (Shanghai, China). HRP‐conjugated goat anti‐mouse (A0350) and anti‐rabbit (A0352) secondary antibodies were obtained from Beyotime (Shanghai, China).

### 4.7 Transmission Electron Microscopy

To assess pyroptotic traits, we fixed cells in 2.5% glutaraldehyde at 4°C overnight, then subjected the samples to post‐fixation with 1% osmium tetroxide. Samples were sequentially dehydrated through an ethanol gradient, embedded in epoxy resin, and sectioned to obtain ultrathin slices. Sections were subsequently stained with uranyl acetate and lead citrate, and ultrastructural changes were examined by transmission electron microscopy. Pyroptotic cells were identified according to characteristic morphological criteria and quantified in five randomly selected fields per sample.

### 4.8 Establishment and Characterization of PDOs

Tumor specimens from patients with GC were enzymatically dissociated using a commercial tissue digestion kit (abs9449; Absin, Shanghai, China). The resulting single‐cell suspensions were embedded in NovoMatrix Organoid Validated, GFR, Phenol Red–Free matrix (NMO‐G‐PF; Novoprotein, Suzhou, China) to generate patient‐derived organoids (PDOs) and cultured in GC organoid medium (OCMHC07; Novoprotein).

Organoid morphology was monitored by bright‐field microscopy using a Zasis microscope (Zasis, China), and organoid diameter and area were quantified with ImageJ software. Cell viability was evaluated using the Beyo3D Calcein/PI Live/Dead Assay Kit (C1375S; Beyotime). For proliferation analysis, PDOs were exposed to 50 µM EdU for 2 h, followed by detection with the Cell‐Light EdU Apollo567 Kit (RiboBio, Guangzhou, China) after fixation and permeabilization. Nuclei were counterstained with DAPI. Live/dead and EdU‐positive signals were visualized by fluorescence microscopy.

### 4.9 Lentiviral Transduction of GC Organoids

Organoids were dissociated into single‐cell suspensions, and 1 × 10^5^ viable cells per group were harvested by centrifugation, with the supernatant discarded for subsequent transduction. Cell pellets were resuspended in 250 µL of lentiviral suspension (multiplicity of infection, MOI = 25) prepared in washing buffer supplemented with 8 µg/mL Polybrene and 10 µM Y‐27632 (Cat. No. 1254, R&D). The cell‐virus mixture was transferred to 48‐well plates, centrifuged at 600 × g for 60 min at 32°C, and then incubated for a further 6 h at 37°C in a humidified 5% CO_2_ incubator. After incubation, transduced cells were collected and centrifuged at 1000 × g for 5 min to remove the viral supernatant. The cell pellet was resuspended in 50 µL basement membrane matrix, seeded into 24‐well plates, and 500 µL complete organoid medium was added after matrix solidification. Puromycin selection was performed 3 days post‐transduction to generate stably transduced organoid lines.

### 4.10 LDH Release Assay

Pyroptosis‐associated loss of membrane integrity was assessed by measuring lactate dehydrogenase (LDH) release with an LDH Cytotoxicity Assay Kit (C0016; Beyotime, Shanghai, China). Before LDH measurement, the original medium was replaced with medium containing 1% fetal bovine serum. To determine maximum LDH release, control wells were treated with the LDH Release Reagent (10% v/v) for 1 h before detection. Subsequently, the plates were centrifuged at 400 × g for 5 min, and 120 µL of the supernatant from each well was transferred to a new 96‐well plate. Absorbance was measured using a microplate reader, and the percentage of LDH release was calculated relative to the maximum release control.

### 4.11 Xenograft Mouse Models

For subcutaneous xenografts, AGS cells (transfected with sh‐NC or sh‐*LTβR*) and HGC‐27 cells (transfected with LV‐NC or Lv‐*LTβR*) were resuspended in PBS:Matrigel (1:1), and 5 × 10^6 cells in 100 µL were injected subcutaneously into the flank of each female nude mouse (4–6 weeks old). Tumor dimensions were measured with calipers every 3 days, and tumor volume was calculated as (length × width^2)/2. When tumors became palpable (day 6 post‐inoculation), mice were randomized to the indicated treatment groups. For the genetic knockdown/overexpression experiments, tumor regions were subjected to fractionated local IR (6 Gy × 4 fractions) administered on days 6, 9, 12, and 15. At the end of the experiment, mice were euthanized by an overdose of pentobarbital sodium, and tumors were harvested for subsequent analyses.

### 4.12 IHC and TdT‐Mediated dUTP Nick End Labeling Staining

Paraffin‐embedded tissue sections (4 µm) were deparaffinized in xylene and rehydrated through graded ethanol. For IHC, antigen retrieval was performed by boiling the slides in EDTA for 20 min. After blocking endogenous peroxidase activity with 3% H_2_O_2_ and nonspecific binding with 5% BSA, the sections were incubated overnight at 4°C with primary antibodies against LTβR and Ki‐67. The slides were then incubated with HRP‐conjugated secondary antibodies and developed with DAB, followed by hematoxylin counterstaining. Staining was evaluated independently by two pathologists blinded to the clinical data. IHC scores were calculated by multiplying staining intensity (0–3) by the proportion of positive cells [99].

To detect cell death in xenograft tumor tissues, TUNEL was conducted using the TUNEL BrightGreen Apoptosis Detection Kit (A112, Vazyme, Nanjing, China) following the manufacturer's guidelines. TUNEL‐positive nuclei were identified via fluorescence microscopy and quantified in randomly selected high‐power fields per tumor sample.

### 4.13 Enzyme‐Linked Immunosorbent Assay

The levels of IL‐1β (KE00021, detection sensitivity 1.5 pg/mL) and IL‐18 (KE00193, detection sensitivity 0.3 pg/mL) in cell supernatant were detected by ELISA kits purchased from Proteintech (Wuhan, China), with the specific experimental methods the same as those described previously [73].

### 4.14 Co‐Immunoprecipitation

Co‐IP assays were carried out using the Pierce Classic Magnetic IP/Co‐IP Kit (88804, Thermo Fisher Scientific, Waltham, MA, USA). In brief, cells expressing Flag‐ or HA‐tagged proteins were lysed in prechilled IP Lysis/Wash Buffer. After centrifugation at 13 000 g for 10 min, protein concentrations in the supernatants were measured. For immunoprecipitation, 500 µg of cell lysate was incubated with 2 µg of anti‐Flag or anti‐HA antibody overnight at 4°C. Subsequently, Pierce Protein A/G Magnetic Beads were added and incubated for 1 h at room temperature to capture the immune complexes. The beads were magnetically separated and washed three times with IP Lysis/Wash Buffer and once with ultrapure water. Bound proteins were eluted by boiling in SDS‐PAGE sample buffer at 96°C–100°C for 10 min and analyzed via Western blotting.

### 4.15 IP‐MS and Enrichment Analysis

Flag‐LTβR protein complexes were isolated with Anti‐FLAG M2 Affinity Gel (Sigma) and resolved by SDS‐PAGE. After in‐gel trypsin digestion, peptides were subjected to analysis on a Q Exactive mass spectrometer (Thermo Fisher Scientific). Raw data were searched against the UniProt human database with Mascot software (version 2.3) for protein identification. Candidate interacting proteins identified by IP‐MS were further analyzed with Metascape to define enriched biological processes and signaling pathways [100].

### 4.16 Nascent Protein Synthesis Analysis

To examine the effect of LTβR on global protein synthesis, nascent translation was measured in AGS cells carrying sh‐NC or sh‐LTβR and in HGC‐27 cells carrying Lv‐NC or Lv‐*LTβR* by SUnSET and Click‐iT HPG assays. For SUnSET analysis, cells were deprived of serum for 6 h and then returned to complete medium containing 10% fetal bovine serum for 1 h to restore translational activity. Cells were subsequently exposed to puromycin at 10 µg/mL for 15 min, and puromycin incorporation into newly synthesized polypeptides was detected by Western blot of total protein lysates with an anti‐puromycin antibody. Nascent protein synthesis was also visualized with the Click‐iT HPG Alexa Fluor 594 Protein Synthesis Assay Kit (C10429, Thermo Fisher Scientific). In this assay, cells were serum‐starved for 6 h, incubated in methionine‐free medium for 1 h to deplete intracellular methionine, and then labeled with 50 µM L‐homopropargylglycine for 1 h. After fixation in 3.7% paraformaldehyde and permeabilization with 0.5% Triton X‐100, cells were subjected to the Click reaction for 45 min in the dark and counterstained with HCS NuclearMask Blue. Fluorescence images were captured with a THUNDER Imager (Leica Microsystems), and Alexa Fluor 594 intensity was quantified as a readout of nascent protein synthesis.

### 4.17 Polysome Profiling

AGS cells expressing sh‐NC or sh‐*LTβR* and HGC‐27 cells expressing Lv‐NC or Lv‐*LTβR* were incubated with cycloheximide (100 µg/mL; Sigma‐Aldrich) at 37°C for 5 min. The medium was removed, and the cells were washed with PBS containing cycloheximide at the same concentration. Cells were then lysed on ice for 15 min in 300 µL of Triton X‐100‐containing lysis buffer. After centrifugation at 13 000 × g for 15 min, the cleared supernatants were collected and layered onto 10%–50% (w/v) linear sucrose gradients prepared in lysis buffer lacking Triton X‐100. Ultracentrifugation was carried out at 27 500 rpm for 4 h at 4°C. Gradient fractions were collected with a Gradient Station (BioComp Instruments, Fredericton, NB, Canada) while absorbance at 254 nm was continuously recorded. RNA was isolated from each fraction and analyzed by qRT‐PCR. Additional experimental details were as described previously [101].

### 4.18 Integrated Multi‐Omics Analysis

For the clinical cohort, LC‐MS/MS proteomic profiling of tumor tissues from the 12 GC patients (n = 7 for responders and n = 5 for non‐responders) was performed by Applied Protein Technology (Shanghai, China). For differential protein screening, statistical significance was determined using the limma package. Proteins satisfying the criteria of a nominal *p* value < 0.05 and |log_2_FC| > 1 were defined as differentially expressed proteins. We employed an integrated multi‐omics approach to comprehensively characterize the shifts in gene expression, translational efficiency, and metabolism resulting from sh‐*LTβR*. Transcriptomic and ribosome profiling services for sh‐NC and sh‐*LTβR* cells (n = 2 per group) were provided by Gene Denovo Biotechnology Co., Ltd (Guangzhou, China). Library construction for RNA‐seq involved total RNA enrichment via Oligo(dT) beads. The Ribo‐seq was performed as follows: ribosomes were stabilized with cycloheximide, digested with RNase I, and ribosome‐protected fragments (RPFs) were isolated. Both library types were sequenced using the Illumina NovaSeq 6000 platform. Data analysis was performed using HISAT2 (for RNA‐seq) or STAR (for Ribo‐seq) for alignment, RSEM for quantification, and DESeq2 for differential analysis (p < 0.05, |log_2_FC| > 1). Concurrently, for untargeted metabolomics, sh‐NC and sh‐*LTβR* cells (n = 6 per group) were analyzed by Applied Protein Technology (Shanghai, China) using a UPLC‐MS/MS system. Metabolites were identified against an in‐house standard database based on retention time, molecular mass (< 10 ppm error), and fragmentation patterns. Differential metabolites were identified using a threshold of VIP > 1.0 and p < 0.05, followed by pathway enrichment analysis using the KEGG database.

### 4.19 RNA Immunoprecipitation Assay

RIP assays were carried out using the BeyoRIP RIP Assay Kit (P1805S, Beyotime, Shanghai, China) in accordance with the manufacturer's instructions. Cells were first lysed in complete RIP lysis buffer. The supernatants were collected and incubated with anti‐PCBP2 antibody or control IgG overnight at 4°C. Subsequently, Protein A/G magnetic beads were added and incubated for 1 h to capture the antigen‐antibody‐RNA complexes. After extensive washing with RIP wash buffer, the immunoprecipitated RNA was purified, and the enrichment of *SARM1* was quantified by RT‐PCR.

### 4.20 RNA Pulldown Assay

RNA pulldown assay was performed as previously described [102]. Briefly, the *SARM1* overexpression vector was constructed by cloning the full‐length SARM1 cDNA sequence into the pcDNA3.1 vector. Using this recombinant plasmid as the template, the SARM1 coding sequence was amplified by PCR with Phanta Max Master Mix (Cat. No. P525‐01; Vazyme, Nanjing, China), followed by restriction digestion with BamHI. PCR products were separated on agarose gels and purified using the FastPure Gel DNA Extraction Mini Kit (Cat. No. DC301‐01; Vazyme, Nanjing, China). For in vitro transcription, sense and antisense SARM1 RNAs were synthesized, biotin‐labeled with the RNAmax‐T7 Kit (Cat. No. C11002‐1; RIBOBIO, Guangzhou, China), and purified. Biotinylated *SARM1* RNAs were incubated with total protein extracts from AGS and HGC‐27 cells at 4°C for 1 h, then incubated with 30 µL of streptavidin‐conjugated beads (Cat. No. HY‐K0208; MedChemExpress) overnight at 4°C to form RNA‐protein complexes. After washing and elution, the bound proteins were analyzed by Western blotting using an anti‐PCBP2 antibody.

### 4.21 Multiplex Immunofluorescence (mIF) Assay

mIF assay was performed as previously described [103]. Briefly, mIF staining was performed on cultured cells (AGS/HGC‐27) and xenograft tumor tissues (CDX model) using the Triple Staining Kit (abs50012, Absin, Shanghai, China) based on the tyramide signal amplification (TSA) principle. Sample Preparation: Cells on coverslips were fixed with 4% paraformaldehyde and permeabilized with 0.5% Triton X‐100. CDX tissue sections were deparaffinized, rehydrated, and subjected to antigen retrieval. Sequential Staining: Staining was conducted in three sequential rounds over separate days. In each round, samples were incubated with one specific primary antibody (LTβR, PCBP2, or TRIM28) overnight at 4°C. On the following day, samples were incubated with the corresponding HRP‐conjugated secondary antibody, followed by reaction with a specific tyramide‐fluorophore (TSA dye). After each round of labeling, the primary and secondary antibodies were stripped (eluted) using heat‐induced antigen retrieval to prevent cross‐reactivity, while the fluorophore signal remained covalently bound to the target. This cycle was repeated three times to label all targets. Imaging: Finally, nuclei were counterstained with DAPI. High‐resolution multi‐channel fluorescence images were acquired using a Zeiss LSM 900 confocal laser scanning microscope (Carl Zeiss, Jena, Germany).

### 4.22 Nuclear and Cytoplasmic Fractionation

A Nuclear and Cytoplasmic Protein Extraction Kit (P0028, Beyotime, Shanghai, China) was employed for the isolation of nuclear and cytoplasmic proteins. Briefly, cells were harvested and incubated with Cytoplasmic Extraction Reagents A and B on ice to release cytoplasmic proteins. After centrifugation, the supernatant was collected as the cytoplasmic fraction. The remaining nuclear pellet underwent resuspension in Nuclear Extraction Reagent, followed by intermittent vortex mixing over 30 min. The lysates were then centrifuged to obtain the nuclear fraction. Finally, the separated fractions were analyzed by Western blotting, with GAPDH (60004‐1‐Ig) and Lamin B1 (12987‐1‐AP) serving as the loading controls for cytoplasmic and nuclear fractions, respectively.

### 4.23 Cellular Thermal Shift Assay (CETSA)

AGS cells were treated with 12.5 µM EMD or DMSO for 4 h and resuspended in PBS containing protease inhibitors. The suspension was divided into seven aliquots. For each temperature point (50°C, 53°C, 56°C, 59°C, 62°C, 65°C, and 68°C), the samples were split into two parts: one half served as the unheated input, while the other was heated in a dry bath for 3 min. Following heating and subsequent cooling for 3 min, all samples (including inputs) underwent three freeze‐thaw cycles. Supernatants were obtained by centrifugation (13 000 rpm, 40 min, 4°C). The protein levels of LTβR were then detected by Western blotting to evaluate its thermal stability.

### 4.24 Virtual Screening and Molecular Docking

Virtual screening was performed using the Schrödinger Suite. The human LTβR structure was prepared using the OPLS4 force field, and a receptor grid was generated centered on Asn40 (20 × 20 × 20 Å box) with a specific hydrogen bond constraint. The Bioactive Compound Library Plus (MCE, 27 524 compounds) was prepared via LigPrep and screened sequentially using the High‐Throughput Virtual Screening (HTVS), Standard Precision (SP), and Extra Precision (XP) docking modes. The top 10% of compounds were retained at each stage. Binding free energies were refined using Prime MM‐GBSA. The top 100 candidates satisfying the criteria (MM‐GBSA △G_bind_ < −40 kcal/mol; XP GScore < −5) were further validated using CS‐DOCK2 with a cutoff of Vina score < −6. Ultimately, two lead compounds were selected.

For protein‐protein docking, the three‐dimensional structures of TRIM28, LTβR, and PCBP2 were retrieved from the UniProt database (https://www.uniprot.org/). The interaction modes of the TRIM28‐LTβR‐PCBP2 complex were predicted using the HDOCK online server (http://hdock.phys.hust.edu.cn/). HDOCK utilizes a global docking strategy based on a Fast Fourier Transform (FFT) algorithm to search for potential binding orientations, and the sampled poses were evaluated using a knowledge‐based scoring function. All docking complexes and molecular interactions were visualized and analyzed using the PyMOL software (Version 3.1.4.1, NY, USA).

### 4.25 SPR Assay

SPR experiments were performed using a Biacore X100 instrument (Cytiva) equipped with a CM5 sensor chip. Recombinant human LTβR protein (C328, Novoprotein, Suzhou, China) was immobilized onto the activated chip surface via standard amine coupling. The small molecule analytes, EMD (HY‐15193A, MCE) and UNBS5162 (HY‐16509, MCE), were diluted into running buffer at various concentrations and injected over the immobilized protein surface. The association and dissociation phases were monitored in real‐time, and the equilibrium dissociation constants (K_D_) were calculated using the Biacore X100 Evaluation Software.

### 4.26 Preparation of cRGD‐Modified EMD‐Loaded Liposomes

cRGD‐modified, EMD‐loaded liposomes were prepared by the thin‐film hydration method. Briefly, SPC, cholesterol, DSPE‐PEG2000‐cRGD, and EMD were accurately weighed at a mass ratio of 6:1:1:1 and co‐dissolved in a chloroform/ethanol mixture (2:1, v/v). The organic solvents were removed by rotary evaporation at 37°C under reduced pressure to form a uniform lipid film. The film was then hydrated with 1 mL of 5% glucose solution under vortex mixing to obtain multilamellar vesicles. These vesicles were further converted into nanosized unilamellar liposomes by probe sonication at 250 W with 5 s on/off pulses for a total duration of 2 min. DiD was added at 0.1% of the total mass of liposomal components. Free DiD and non‐encapsulated materials were removed by size‐exclusion chromatography using Sephadex G‐75. Lyophilized liposomal formulations were dissolved in DMSO, filtered to remove insoluble impurities, and then analyzed against this calibration curve to quantify the drug loading (DL%) and encapsulation efficiency (EE%). The drug loading and encapsulation efficiency of cRGD‐Lipo@EMD were determined with a visible spectrophotometer. The physicochemical properties of the liposomes, including hydrodynamic diameter, zeta potential, and polydispersity index, were measured by dynamic light scattering, and liposome morphology was further examined by transmission electron microscopy.

### 4.27 In Vitro Release of EMD From Liposomes

The in vitro release profile of EMD from cRGD‐Lipo@EMD was evaluated by a dialysis‐based method. Briefly, 2 mL of cRGD‐Lipo@EMD or free EMD solution was transferred into a dialysis bag with a molecular weight cut‐off of 3500 Da and immersed in 30 mL of release medium composed of PBS (pH 7.4) containing 1% (v/v) Tween 80. The release system was maintained at 37°C with constant shaking at 100 rpm. At predetermined time points, 1 mL of the external medium was collected for analysis and immediately replaced with an equal volume of fresh medium to preserve sink conditions. The concentration of EMD in the collected samples was determined using a UV spectrophotometer.

### 4.28 Stability of Liposomes in 10% FBS

The stability of cRGD‐Lipo@EMD under serum‐containing conditions was assessed by mixing the liposome suspension with an equal volume of RPMI‐1640 medium supplemented with 10% FBS and incubating the mixture at 37°C for 48 h. At designated time points, aliquots of the suspension were collected and analyzed using a Zetasizer.

### 4.29 In Vitro Cellular Uptake of Liposomes

Cellular uptake of DiD‐labeled liposomes was evaluated in AGS cells. Cells were seeded in culture plates and incubated with equivalent amounts of DiD‐labeled liposomes for the indicated time. After incubation, the cells were washed with phosphate‐buffered saline to remove unbound liposomes. Fluorescence images were acquired using a fluorescence microscope, and quantitative uptake was further analyzed by flow cytometry.

### 4.30 In Vivo Tumor‐targeting Assay and Safety Evaluation

Female nude mice (4–6 weeks old) were randomly divided into six groups (n = 5 per group): vehicle control, EMD monotherapy, radiotherapy alone, EMD plus radiotherapy, cRGD‐Lipo plus radiotherapy, and cRGD‐Lipo@EMD plus radiotherapy. All agents were administered via tail vein injection twice weekly at an EMD dose of 5 mg/kg. Local tumor irradiation at a dose of 6 Gy was delivered 24 h after each drug administration. At the end of the experiment, mice were euthanized by an overdose of pentobarbital sodium, and tumors were excised and weighed. The expression levels of LTβR and Ki‐67 in tumor tissues were then examined by IHC. To evaluate the in vivo tumor‐targeting capacity of the liposomal formulations, AGS tumor‐bearing mice were intravenously injected with DiD‐cRGD‐Lipo@EMD or DiD‐labeled liposomes. Fluorescence signals were monitored at predetermined time points using an in vivo imaging system (PerkinElmer IVIS Lumina III). Upon completion of the observation period, mice were euthanized by an overdose of pentobarbital sodium, and tumors together with major organs were collected for ex vivo fluorescence imaging to assess biodistribution.

### 4.31 Database Resources and Analysis

EMD‐interacting genes and drug targets were predicted using the Comparative Toxicogenomics database [104] (CTD, https://ctdbase.org/). Pan‐cancer single‐cell data from the Cancer Immunology Data Engine [105] (CIDE, https://cide.ccr.cancer.gov/) verified higher *LTβR* expression in tumor cells than other tumor microenvironment cell types. LT*β*R protein expression and immunofluorescence localization were validated through the Human Protein Atlas [106] (HPA, https://www.proteinatlas.org/).

### 4.32 Enrichment Analysis

Single‐sample gene set enrichment analysis (ssGSEA) was performed to investigate the correlation between the expression of *LTβR*, *TRIM28*, *PCBP2* and *SARM1*, and radiotherapy‐related biological pathways, using bulk mRNA sequencing data from the TCGA‐STAD cohort. Radiotherapy‐associated gene sets were retrieved from the Gene Ontology (GO) database in the Molecular Signatures Database (MsigDB) via the keyword “radiotherapy.” Pearson correlation analysis was subsequently conducted to evaluate the association between the ssGSEA enrichment scores of these radiotherapy‐related gene sets and the expression levels of the four target genes.

To further explore the correlation between *LTβR* expression and molecular signatures from the MsigDB Hallmark gene sets, TCGA‐STAD samples were dichotomized into LTβR high‐expression and low‐expression groups using the median expression level of LTβR as the cutoff threshold. Differential expression analysis between the two groups was performed using the limma. All genes were ranked by the log2 fold change (log2FC) derived from the differential expression analysis, and gene set enrichment analysis (GSEA) was implemented using the clusterProfiler R package (version 4.8.3) [107]. The normalized enrichment score (NES) and false discovery rate (FDR)‐adjusted q‐value were used to define statistically significant enrichment.

For complementary GO enrichment analysis, an input gene list was constructed based on EMD‐interacting genes predicted by the CTD database, and radiotherapy‐related enrichment terms were extracted and visualized.

### 4.33 Single‐Cell RNA Sequencing (scRNA‐seq) Analysis

Public scRNA‐seq data of human GC and matched adjacent tissues (accession number: GSE183904) [108] were downloaded as raw count matrices, and all analyses were performed in R (v4.3.1) using the Seurat package (v4.3.0.1) [109]. A Seurat object was generated for each sample with the thresholds of min.cells = 5 and min.features = 300. Low‐quality cells were filtered out according to library size, gene complexity, and the proportion of mitochondrial, ribosomal, and hemoglobin transcripts, with retention criteria set as nCount_RNA > 500, nFeature_RNA < 6000, mitochondrial RNA < 20%, ribosomal RNA < 40%, and hemoglobin < 1%. After log‐normalization of the data, highly variable genes were identified, and cell cycle scores for S and G2/M phases were calculated. These scores, together with the proportions of mitochondrial and ribosomal transcripts, were regressed out during data scaling. PCA was performed, and putative doublets were removed using DoubletFinder (v2.0.4) with sample‐specific expected doublet rates [110]. Batch effects across samples were corrected via the Harmony algorithm (v1.2.3) [111], and UMAP/t‐SNE embeddings were generated based on the Harmony‐corrected results. Neighbor graphs and cell clustering were constructed using the first 15 Harmony dimensions, with a resolution of 0.2. Cluster‐specific marker genes were identified using the FindAllMarkers function (only positive markers with log_2_ fold‐change > 0.5 were retained), and the top markers of each cluster were used for cell type annotation. Cell types were assigned by manual curation based on canonical markers of immune, stromal, and epithelial lineages. Downstream visualization and exploratory analyses were conducted using scRNAtoolVis (v0.0.7).

### 4.34 Statistical Analysis

Statistical analyses were performed using GraphPad Prism software (version 10.4.2) and R (version 4.3.1). All quantitative data are presented as mean ± standard deviation (SD), with the number of independent experiments or biological replicates specified in each figure legend. For comparisons between two independent groups, an unpaired two‐tailed Student's t‐test was used. For comparisons across three or more groups, one‐way ANOVA followed by Tukey's post‐hoc test was applied. For dose‐response curves, time‐course experiments (including tumor growth curves) and two‐factor experimental designs, two‐way ANOVA followed by either Bonferroni's multiple comparisons test was used as specified in each figure legend. The two‐sided t test was used to compare cumulative distribution functions of translational efficiency. A two‐sided *p* value < 0.05 was considered statistically significant.

## Author Contributions

Z.W.J., J.Y.L., and Z.C.W. performed the experiments. L.X.W. and L.Z. performed the majority of data and statistical analysis. Z.G.Z. and C.Y. were responsible for TMA construction and performing the IHC experiments. X.W.J. and H.Y.L. conceived and designed experiments. All authors read and approved the final manuscript.

## Ethics Statement

Human materials used in this study were approved by the Institutional Research Ethics Committee at Affiliated Hospital of Nantong University (2021‐L018). We obtained signed informed consents from all patients participating in the study. Animal experiments were approved by the Institutional Research Ethics Committee at the Medical School of Nantong University (S20210225‐009). The Institutional Research Ethics Committee adheres to the guidelines of the International Council for Laboratory Animal Science (ICLAS).

## Conflicts of Interest

The authors declare that they have no known competing financial interests or personal relationships that could have appeared to influence the work reported in this paper.

## Supporting information




**Supporting File 1**: advs76157‐sup‐0001‐SuppMat.docx.

**Supporting File 2**: advs76157‐sup‐0002‐Uncropped_Western_ blot_images.pdf.

**Supporting File 3**: advs76157‐sup‐0003‐TableS1–3.xlsx.

## Data Availability

The metabolomics data, IP/MS data, RNA‐seq and Ribo‐seq supporting the findings of this study have been deposited in publicly accessible permanent repositories. Specifically, the metabolomics dataset is available in the MetaboLights repository under accession number MTBLS13576; the IP/MS proteomics dataset is available in the iProX repository under accession number IPX0014907000; and the RNA‐seq and Ribo‐seq datasets are available in the NCBI BioProject repository under accession number PRJNA1394493. Previously published scRNA‐seq data that were reanalyzed here are available under accession code GSE183904 [108] from GEO. TCGA‐STAD RNA sequencing data and corresponding clinical information were retrieved from the UCSC Xena platform (https://xena.ucsc.edu/). All other data supporting the findings of this study are available from the corresponding authors on reasonable request.
