# Supplementary material for: Radiation‐Induced Tumor‐Intrinsic LTβR N‐Glycosylation Suppresses Pyroptosis Through TRIM28‐Mediated PCBP2 SUMOylation to Promote Gastric Cancer Radioresistance
Source: Adv Sci (Weinh). 2026 Jun 22;13(51):e76157. doi: 10.1002/advs.76157 (PMC13336884; doi:10.1002/advs.76157)
Supplement: Supplementary file 1 [file ADVS-13-e76157-s001.pdf]

Figure 3F

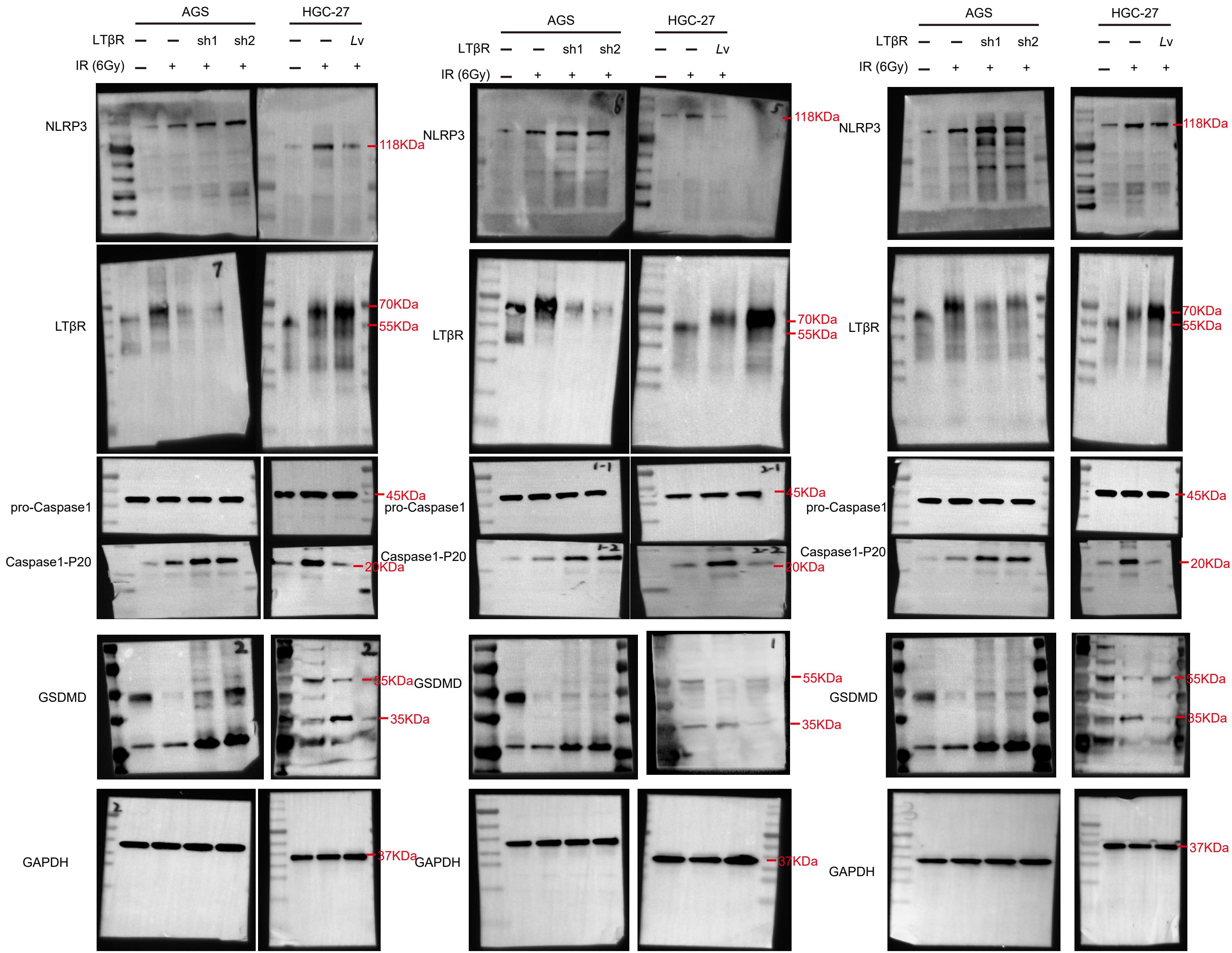
AGS
HGC-27
LTβR
sh1
sh2
Lv
IR (6Gy)
NLRP3
LTβR
pro-Caspase1
Caspase1-P20
GSDMD
GAPDH
118KDa
70KDa
55KDa
45KDa
20KDa
55KDa
35KDa
37KDa

Figure 4F

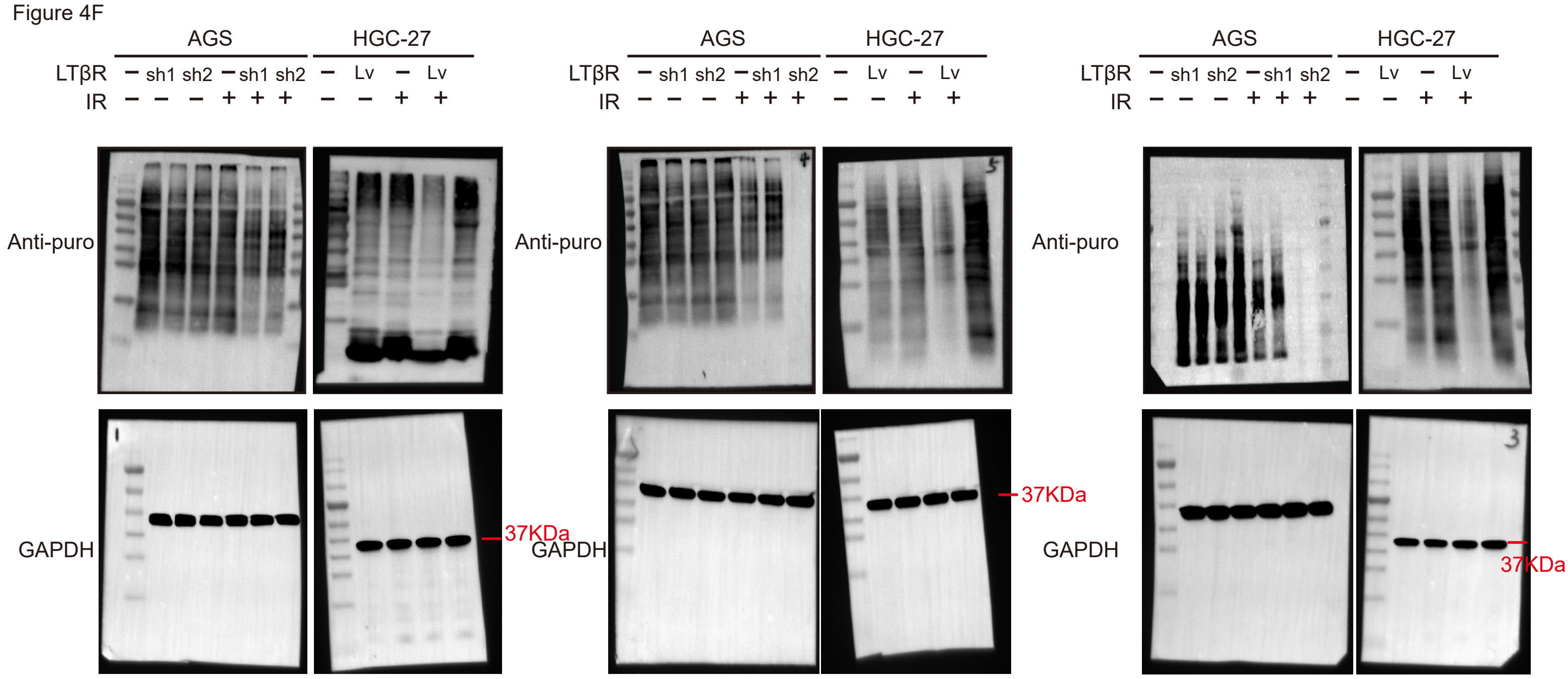

Figure 4P

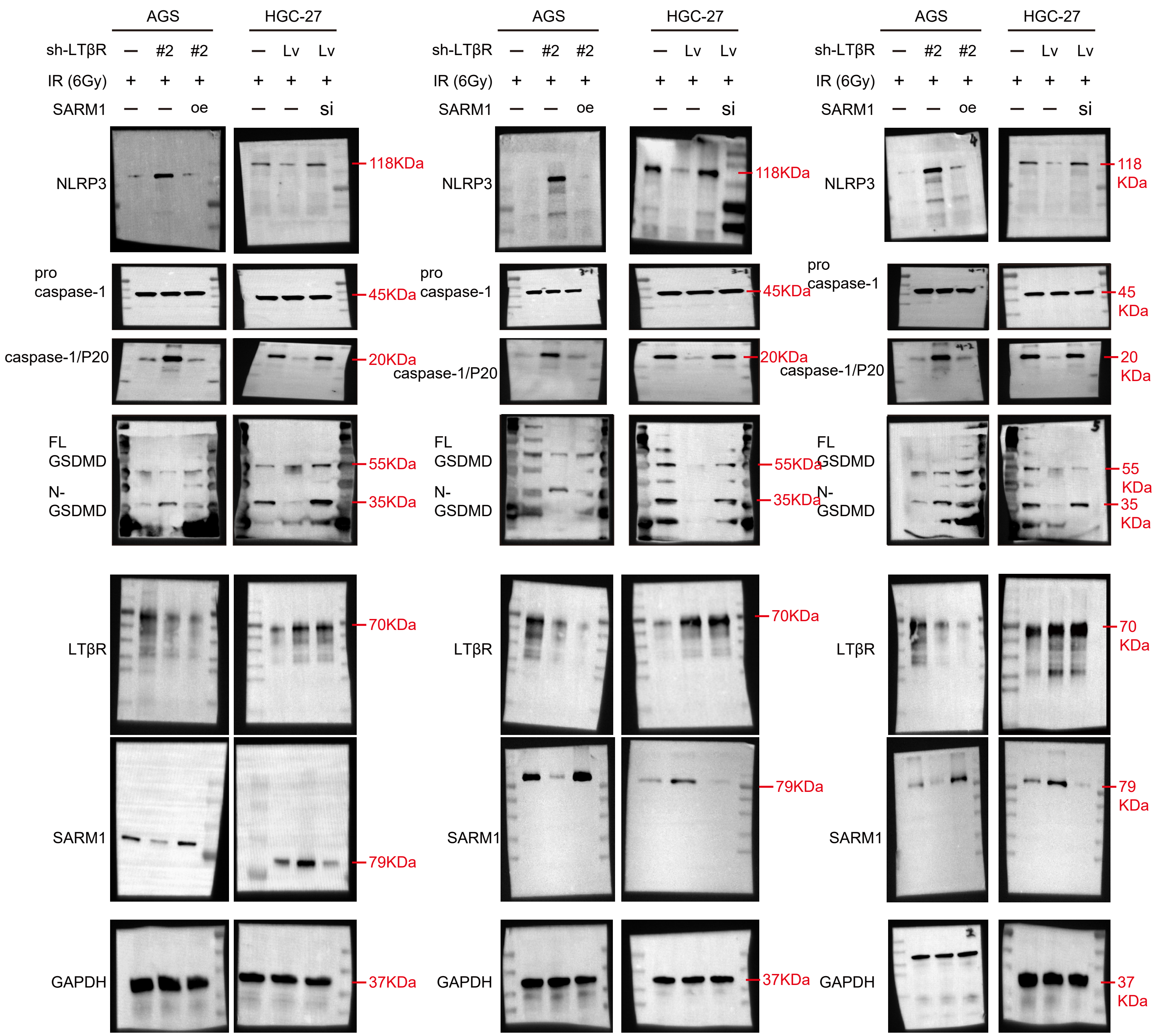

Figure 5C

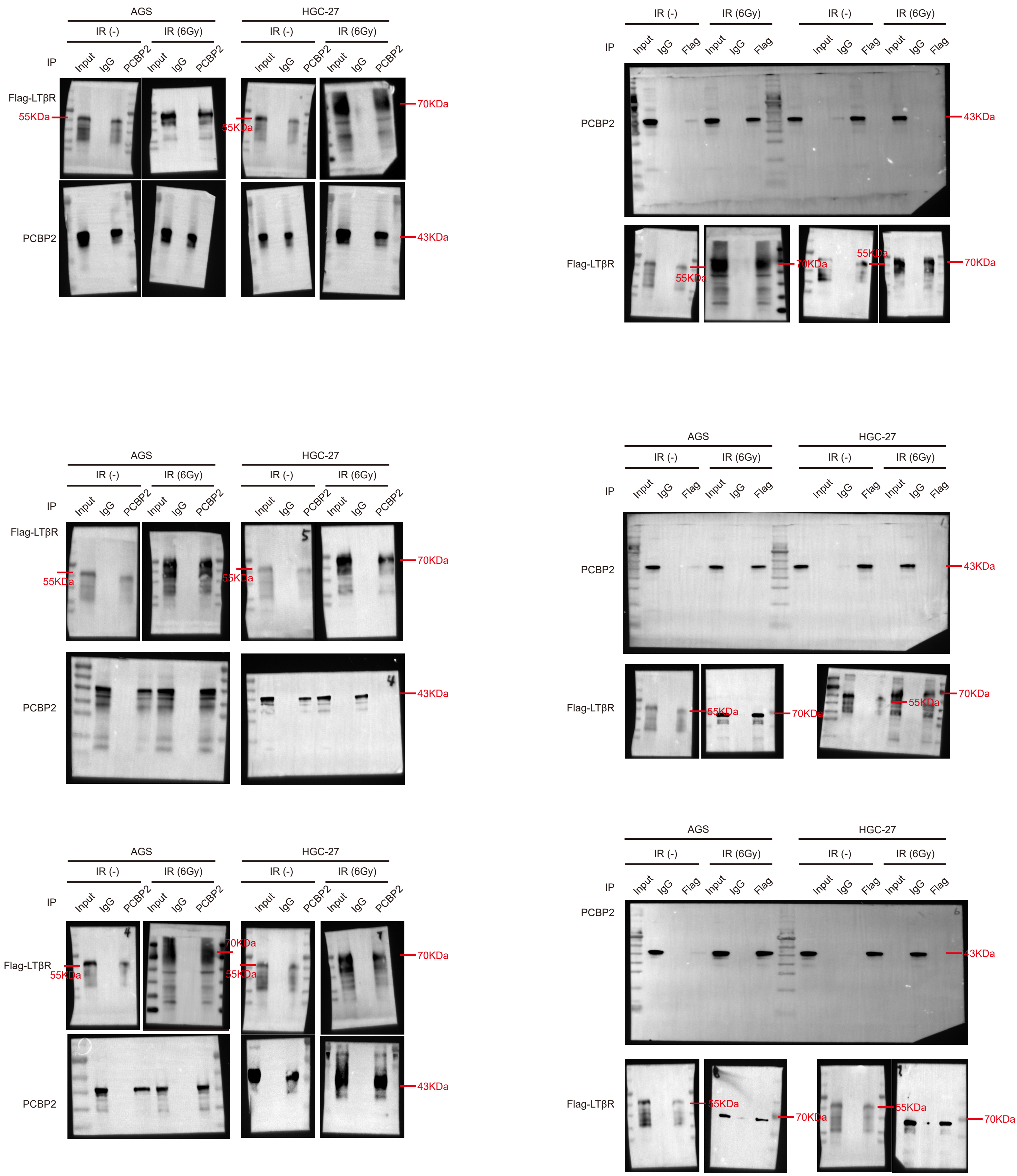
AGS
HGC-27
IR (-)
IR (6Gy)
IP
Input
IgG
PCBP2
Flag
Flag-LTβR
PCBP2
55KDa
70KDa
43KDa

Figure 5J

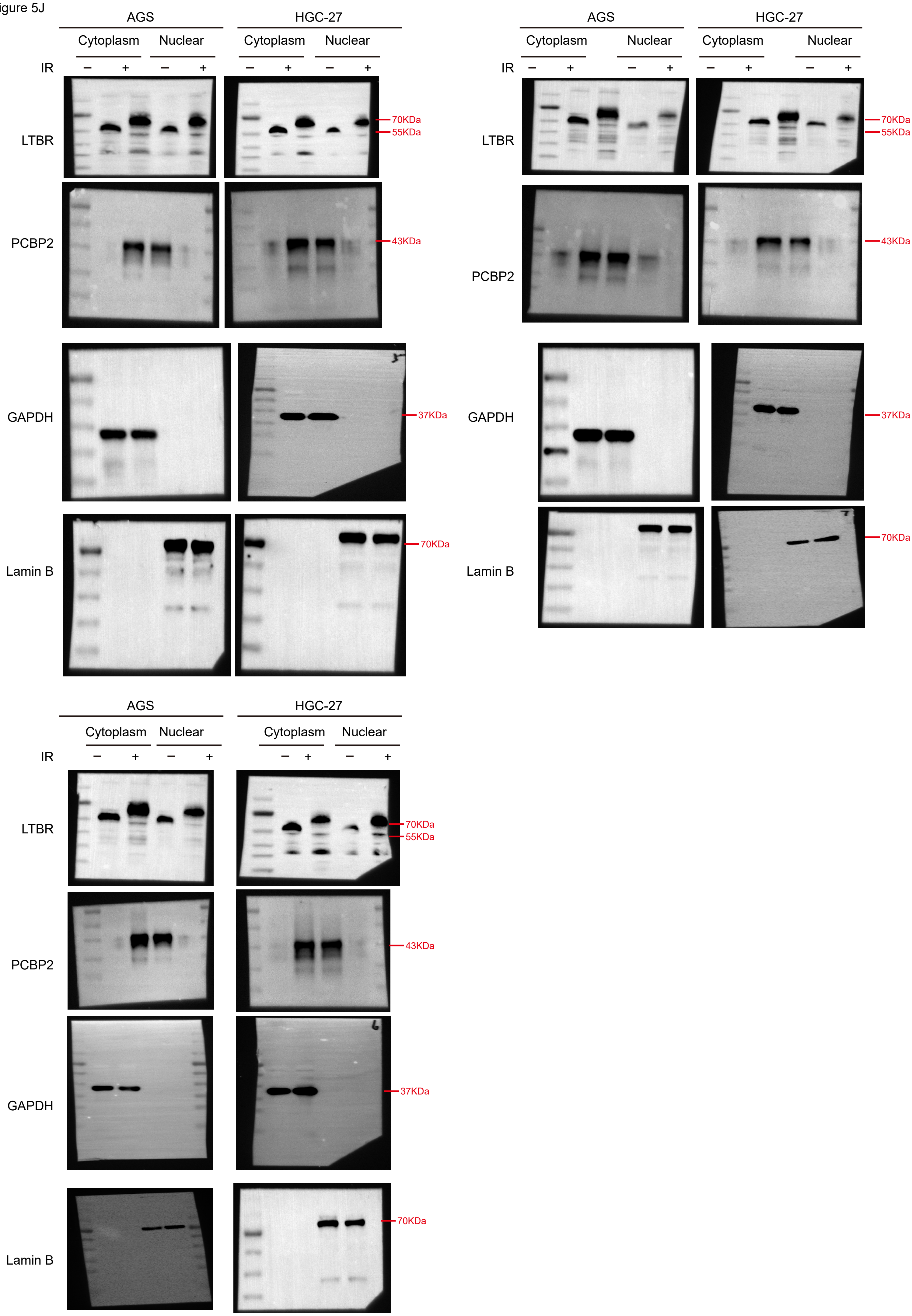
AGS
HGC-27
Cytoplasm
Nuclear
IR
−
+
LTBR
PCBP2
GAPDH
Lamin B
70KDa
55KDa
43KDa
37KDa

Figure 5MN

M

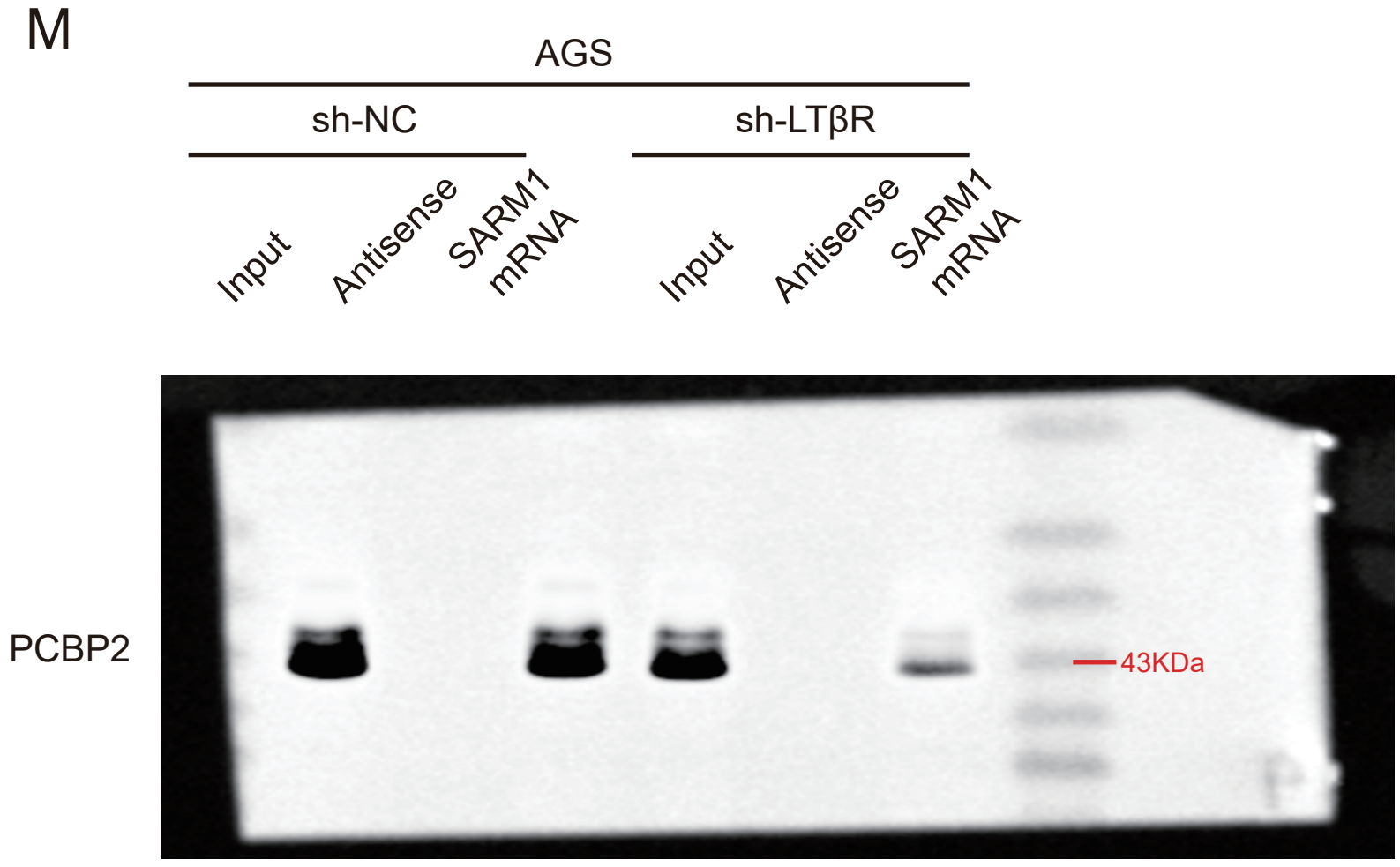

N

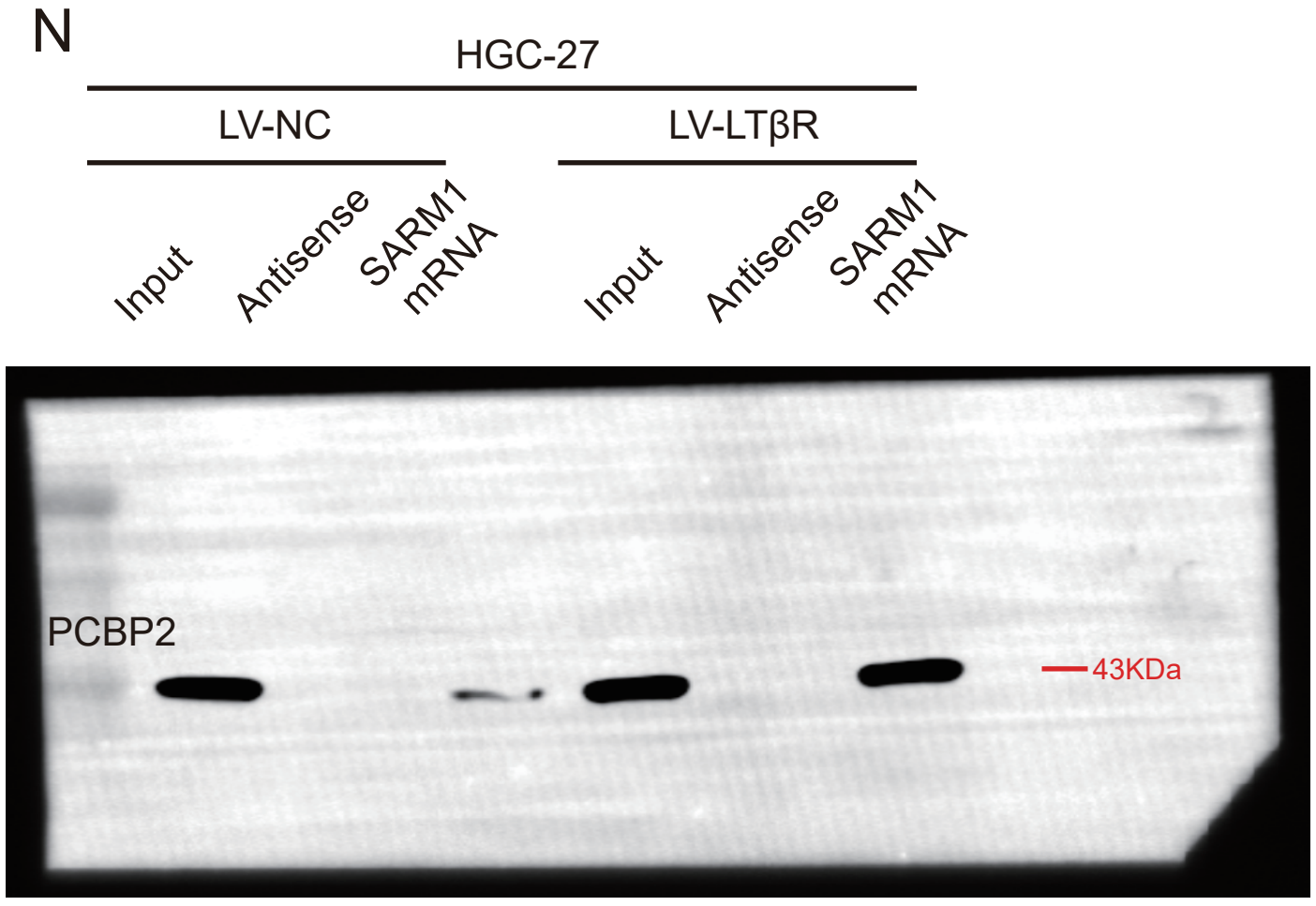

M

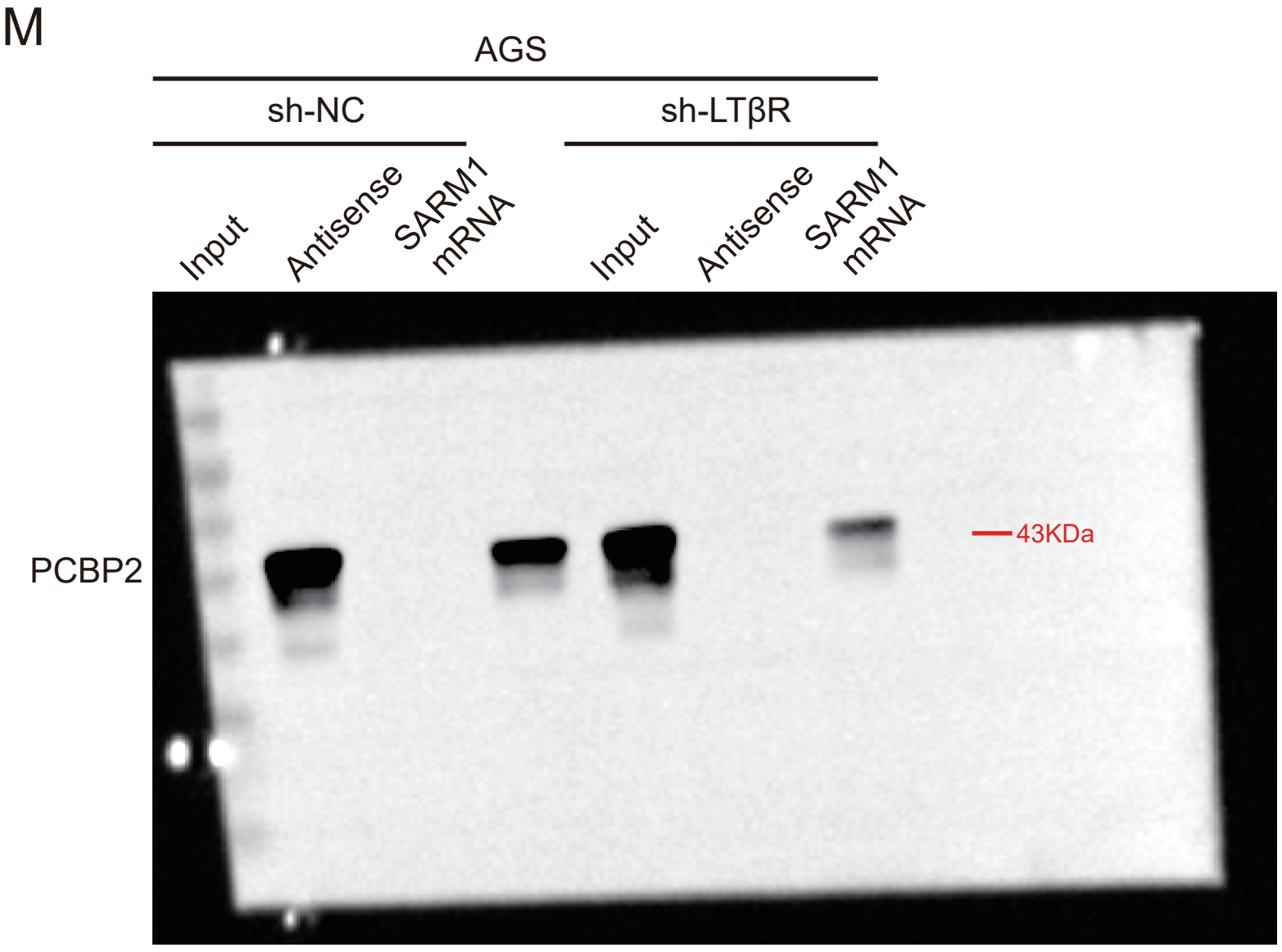

N

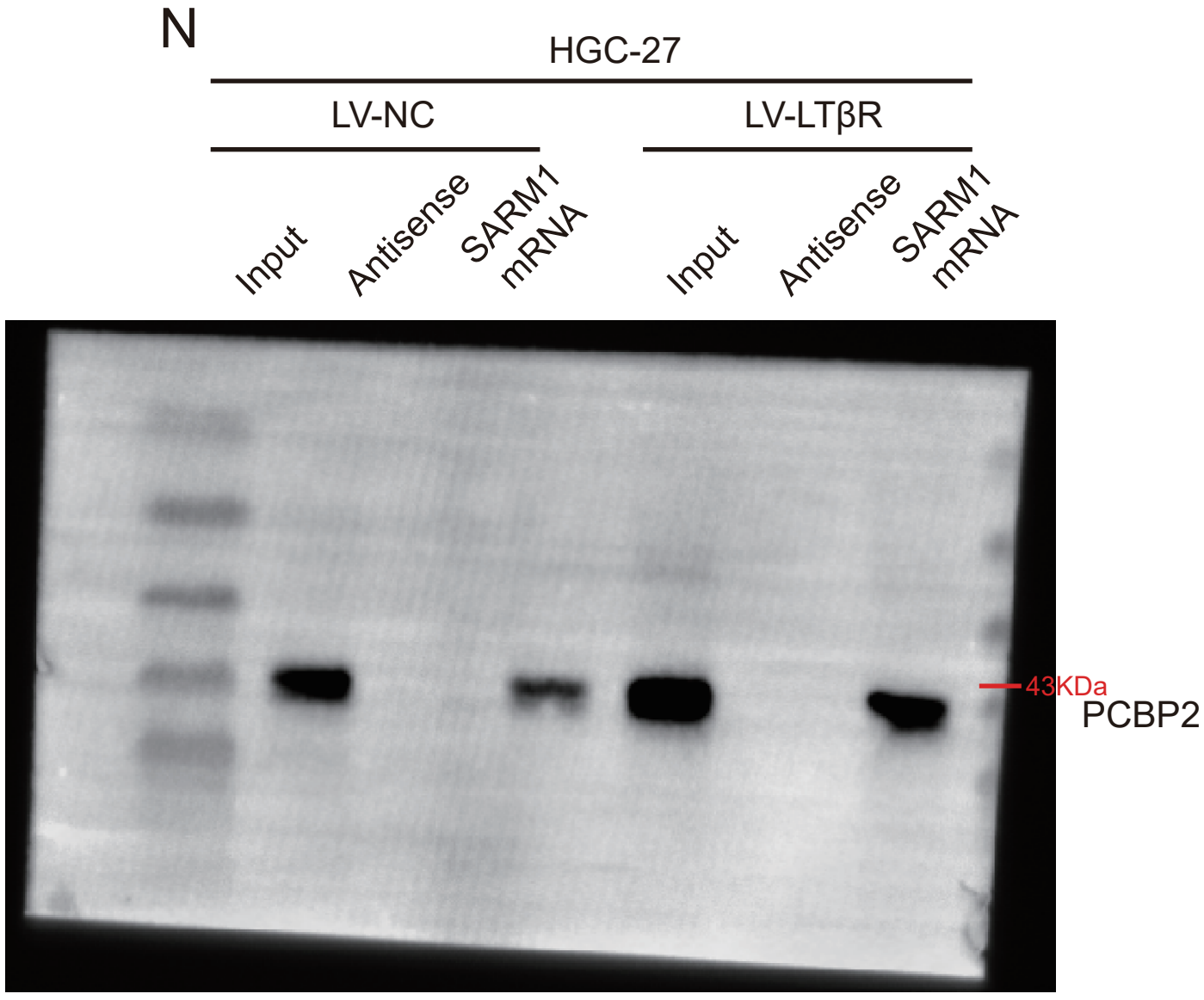

M

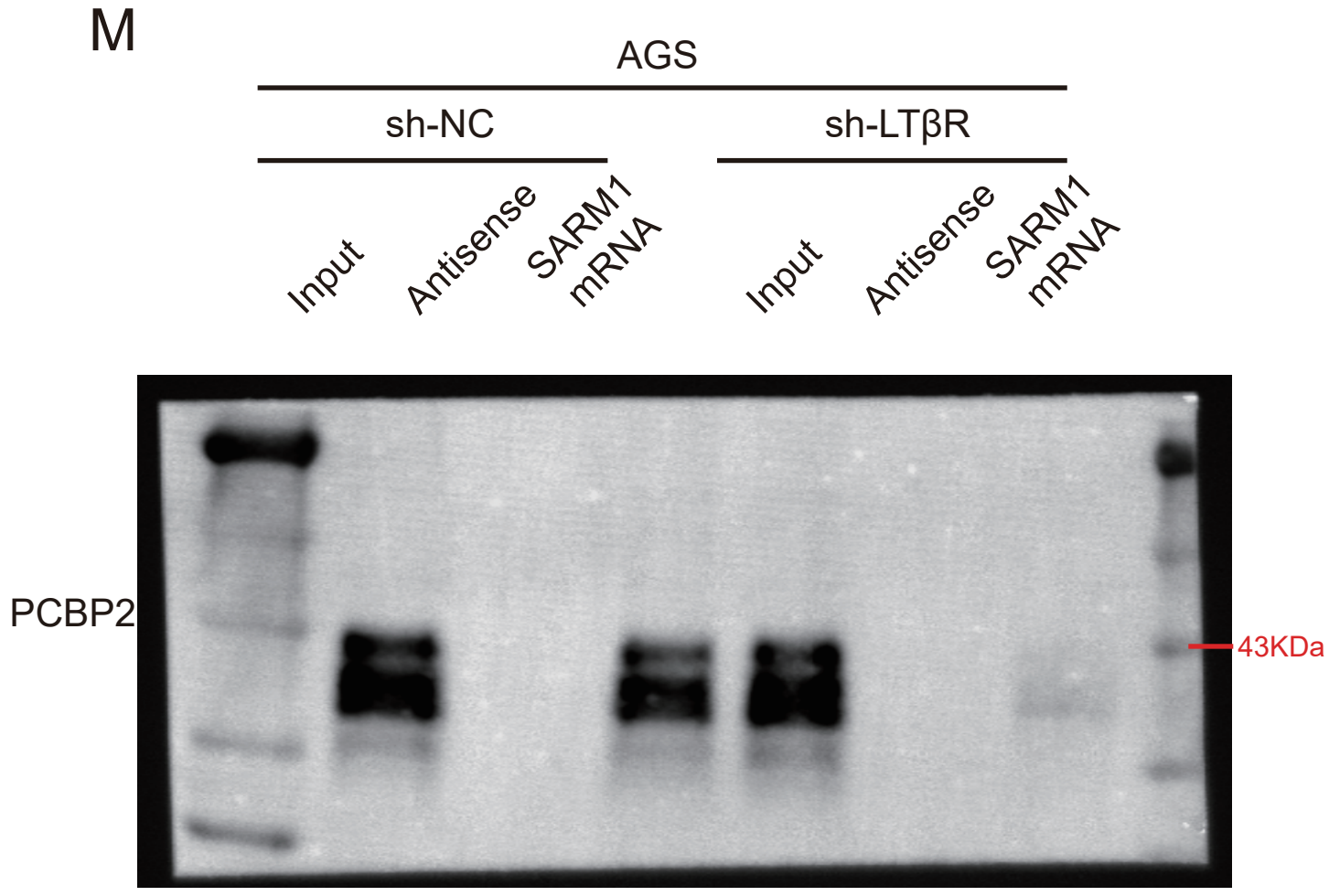

N

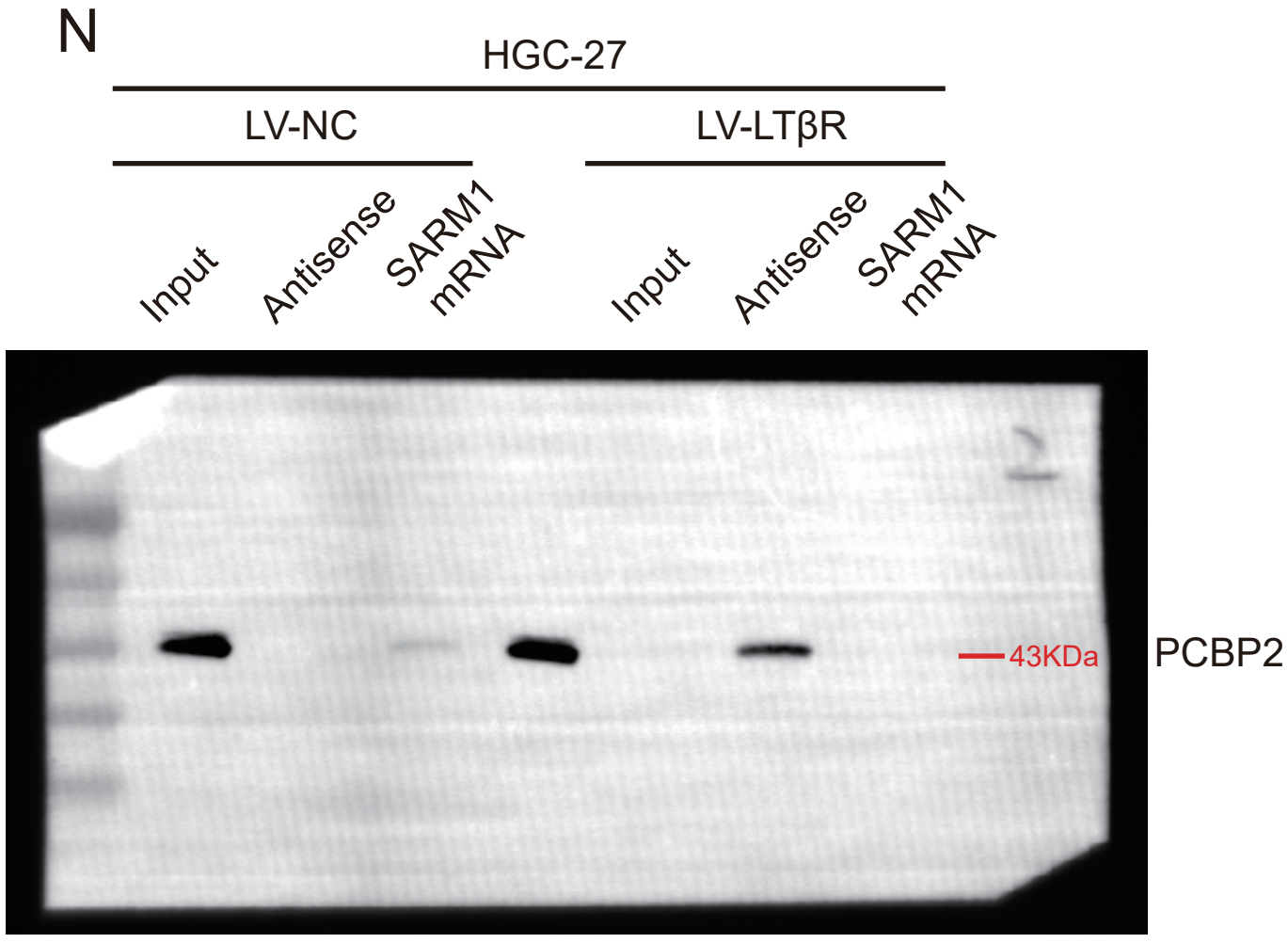

Figure 6A-D

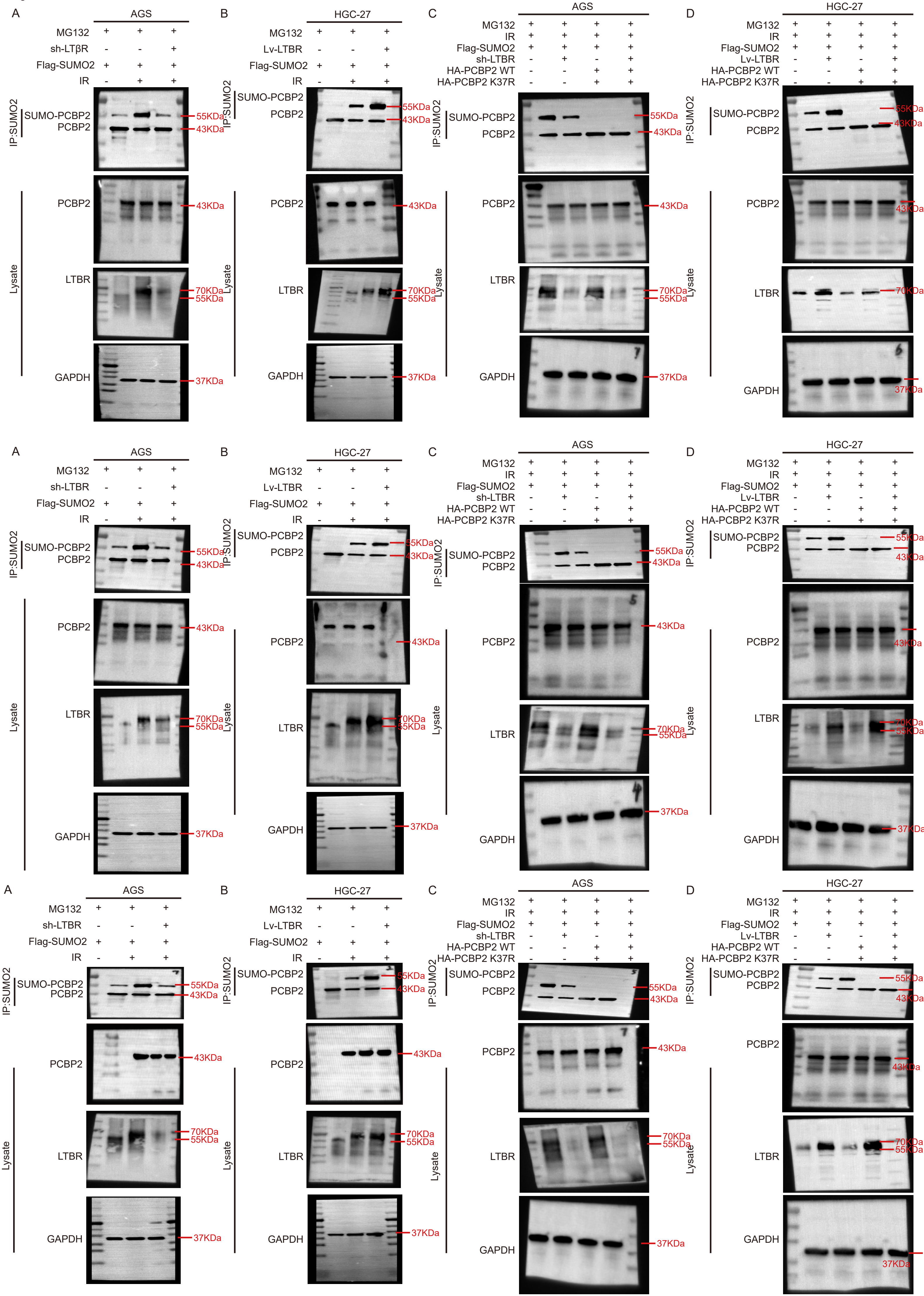
A
AGS
MG132 + + +
sh-LTβR - - +
Flag-SUMO2 + + +
IR - + +
IP:SUMO2
SUMO-PCBP2
PCBP2
55KDa
43KDa
Lysate
PCBP2
43KDa
LTBR
70KDa
55KDa
GAPDH
37KDa
B
HGC-27
MG132 + + +
Lv-LTBR - - +
Flag-SUMO2 + + +
IR - + +
IP:SUMO2
SUMO-PCBP2
PCBP2
55KDa
43KDa
Lysate
PCBP2
43KDa
LTBR
70KDa
55KDa
GAPDH
37KDa
C
AGS
MG132 + + + +
IR + + + +
Flag-SUMO2 + + + +
sh-LTBR - + - +
HA-PCBP2 WT - - + +
HA-PCBP2 K37R - - + +
IP:SUMO2
SUMO-PCBP2
PCBP2
55KDa
43KDa
Lysate
PCBP2
43KDa
LTBR
70KDa
55KDa
GAPDH
37KDa
D
HGC-27
MG132 + + + +
IR + + + +
Flag-SUMO2 + + + +
Lv-LTBR - + - +
HA-PCBP2 WT - - + +
HA-PCBP2 K37R - - + +
IP:SUMO2
SUMO-PCBP2
PCBP2
55KDa
43KDa
Lysate
PCBP2
43KDa
LTBR
70KDa
GAPDH
37KDa
A
AGS
MG132 + + +
sh-LTBR - - +
Flag-SUMO2 + + +
IR - + +
IP:SUMO2
SUMO-PCBP2
PCBP2
55KDa
43KDa
Lysate
PCBP2
43KDa
LTBR
70KDa
55KDa
GAPDH
37KDa
B
HGC-27
MG132 + + +
Lv-LTBR - - +
Flag-SUMO2 + + +
IR - + +
IP:SUMO2
SUMO-PCBP2
PCBP2
55KDa
43KDa
Lysate
PCBP2
43KDa
LTBR
70KDa
55KDa
GAPDH
37KDa
C
AGS
MG132 + + + +
IR + + + +
Flag-SUMO2 + + + +
sh-LTBR - + - +
HA-PCBP2 WT - - + +
HA-PCBP2 K37R - - + +
IP:SUMO2
SUMO-PCBP2
PCBP2
55KDa
43KDa
Lysate
PCBP2
43KDa
LTBR
70KDa
55KDa
GAPDH
37KDa
D
HGC-27
MG132 + + + +
IR + + + +
Flag-SUMO2 + + + +
Lv-LTBR - + - +
HA-PCBP2 WT - - + +
HA-PCBP2 K37R - - + +
IP:SUMO2
SUMO-PCBP2
PCBP2
55KDa
43KDa
Lysate
PCBP2
43KDa
LTBR
70KDa
55KDa
GAPDH
37KDa
A
AGS
MG132 + + +
sh-LTBR - - +
Flag-SUMO2 + + +
IR - + +
IP:SUMO2
SUMO-PCBP2
PCBP2
55KDa
43KDa
Lysate
PCBP2
43KDa
LTBR
70KDa
55KDa
GAPDH
37KDa
B
HGC-27
MG132 + + +
Lv-LTBR - - +
Flag-SUMO2 + + +
IR - + +
IP:SUMO2
SUMO-PCBP2
PCBP2
55KDa
43KDa
Lysate
PCBP2
43KDa
LTBR
70KDa
55KDa
GAPDH
37KDa
C
AGS
MG132 + + + +
IR + + + +
Flag-SUMO2 + + + +
sh-LTBR - + - +
HA-PCBP2 WT - - + +
HA-PCBP2 K37R - - + +
IP:SUMO2
SUMO-PCBP2
PCBP2
55KDa
43KDa
Lysate
PCBP2
43KDa
LTBR
70KDa
55KDa
GAPDH
37KDa
D
HGC-27
MG132 + + + +
IR + + + +
Flag-SUMO2 + + + +
Lv-LTBR - + - +
HA-PCBP2 WT - - + +
HA-PCBP2 K37R - - + +
IP:SUMO2
SUMO-PCBP2
PCBP2
55KDa
43KDa
Lysate
PCBP2
43KDa
LTBR
70KDa
55KDa
GAPDH
37KDa

Figure 6E-F and 6K-L

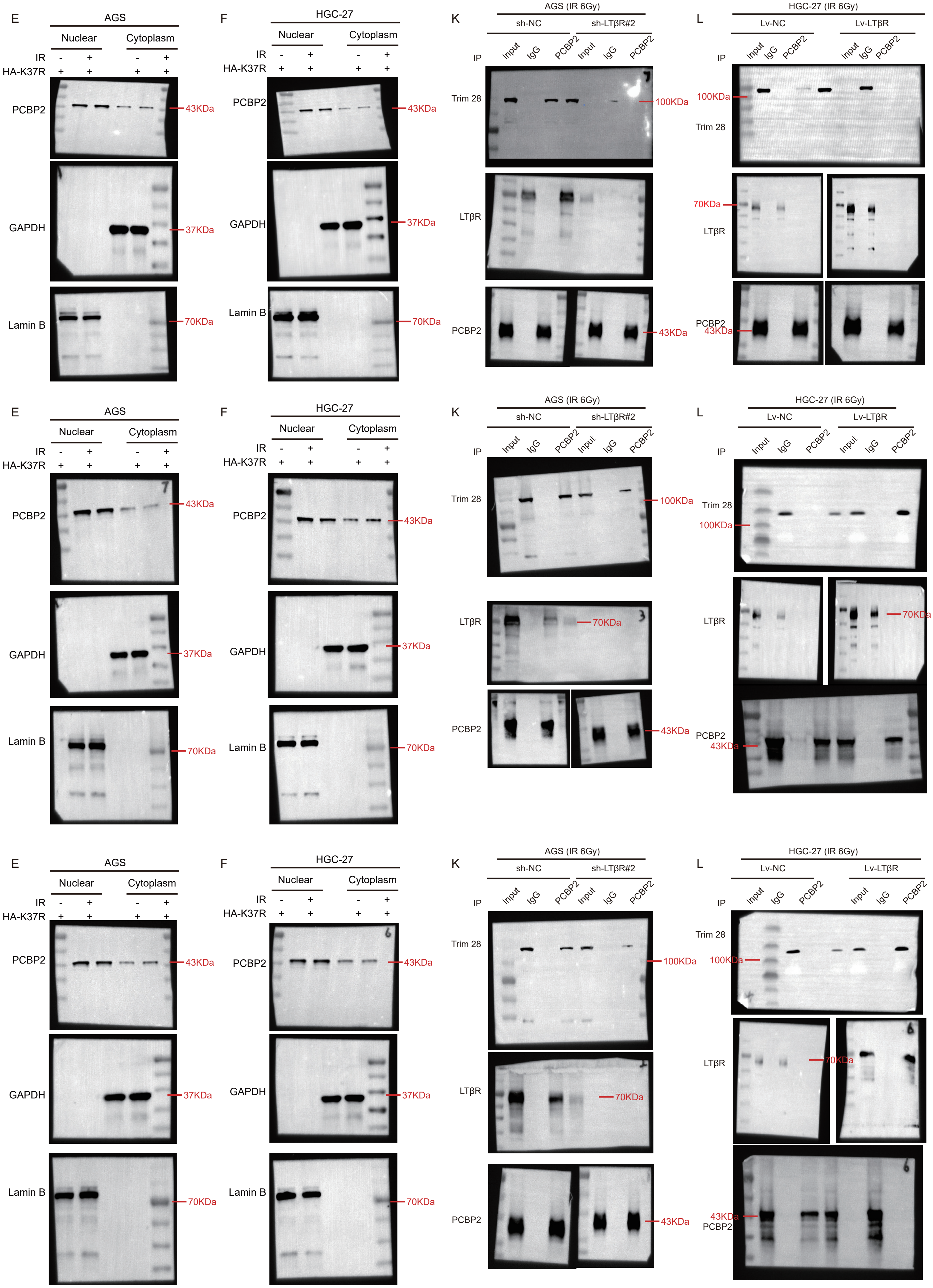

Figure 6M-N

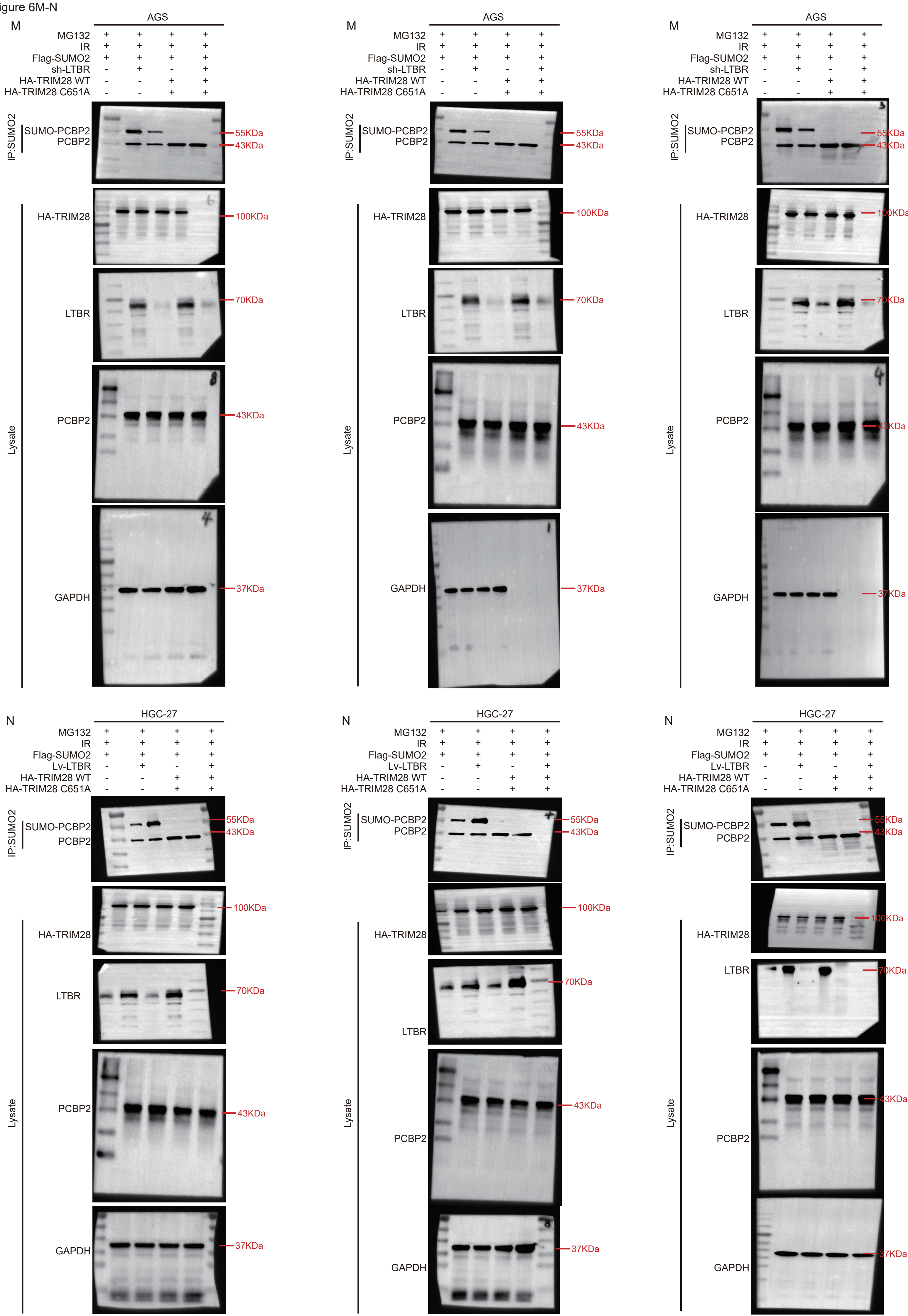

M
AGS
MG132
IR
Flag-SUMO2
sh-LTBR
HA-TRIM28 WT
HA-TRIM28 C651A
IP:SUMO2
SUMO-PCBP2
PCBP2
55KDa
43KDa
Lysate
HA-TRIM28
100KDa
LTBR
70KDa
PCBP2
43KDa
GAPDH
37KDa
N
HGC-27
MG132
IR
Flag-SUMO2
Lv-LTBR
HA-TRIM28 WT
HA-TRIM28 C651A
IP:SUMO2
SUMO-PCBP2
PCBP2
55KDa
43KDa
Lysate
HA-TRIM28
100KDa
LTBR
70KDa
PCBP2
43KDa
GAPDH
37KDa

Figure 7A-D

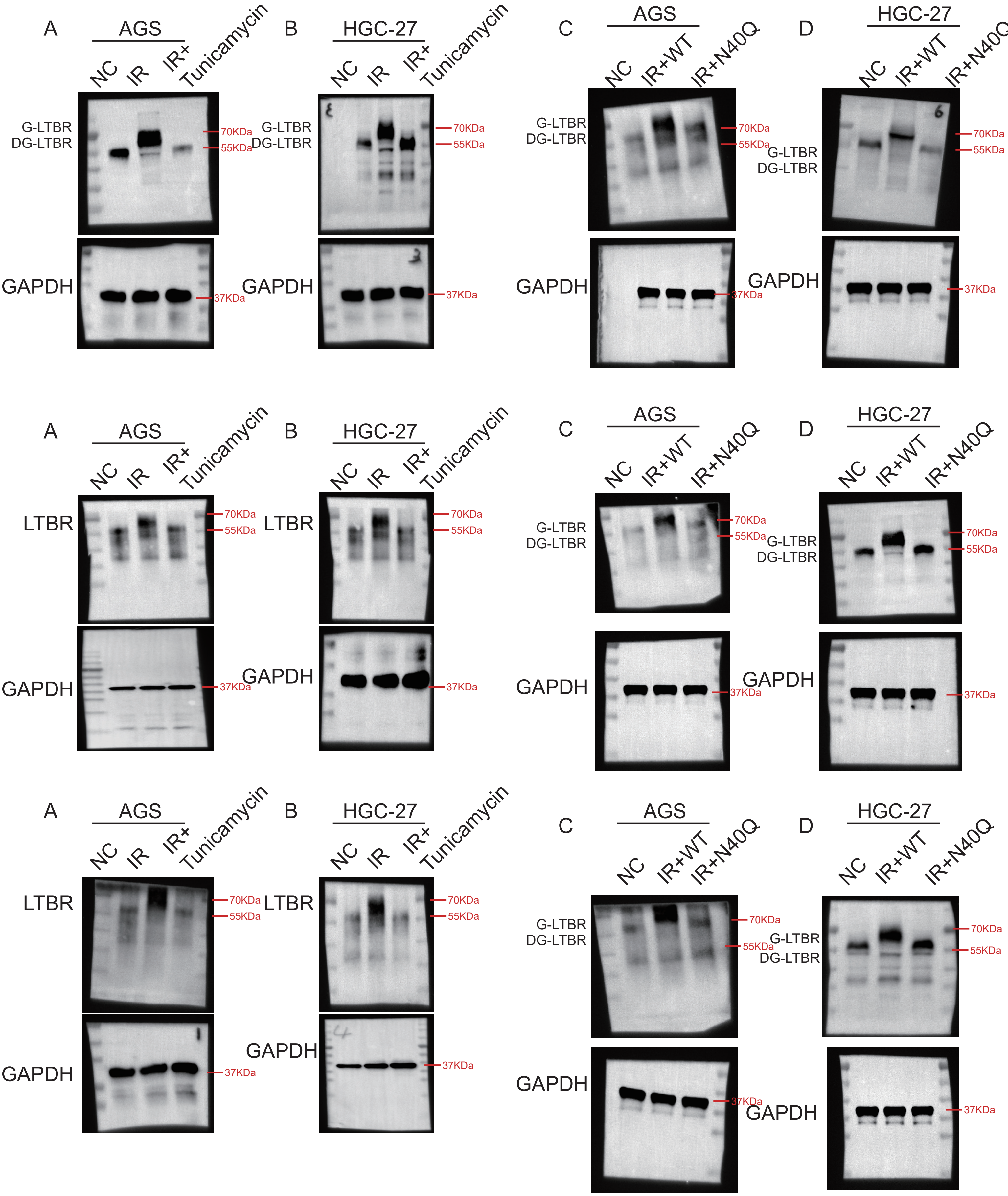
A
AGS
NC
IR
IR+ Tunicamycin
G-LTBR
DG-LTBR
70KDa
55KDa
GAPDH
37KDa
B
HGC-27
NC
IR
IR+ Tunicamycin
G-LTBR
DG-LTBR
70KDa
55KDa
GAPDH
37KDa
C
AGS
NC
IR+WT
IR+N40Q
G-LTBR
DG-LTBR
70KDa
55KDa
GAPDH
37KDa
D
HGC-27
NC
IR+WT
IR+N40Q
G-LTBR
DG-LTBR
70KDa
55KDa
GAPDH
37KDa
A
AGS
NC
IR
IR+ Tunicamycin
LTBR
70KDa
55KDa
GAPDH
37KDa
B
HGC-27
NC
IR
IR+ Tunicamycin
LTBR
70KDa
55KDa
GAPDH
37KDa
C
AGS
NC
IR+WT
IR+N40Q
G-LTBR
DG-LTBR
70KDa
55KDa
GAPDH
37KDa
D
HGC-27
NC
IR+WT
IR+N40Q
G-LTBR
DG-LTBR
70KDa
55KDa
GAPDH
37KDa
A
AGS
NC
IR
IR+ Tunicamycin
LTBR
70KDa
55KDa
GAPDH
37KDa
B
HGC-27
NC
IR
IR+ Tunicamycin
LTBR
70KDa
55KDa
GAPDH
37KDa
C
AGS
NC
IR+WT
IR+N40Q
G-LTBR
DG-LTBR
70KDa
55KDa
GAPDH
37KDa
D
HGC-27
NC
IR+WT
IR+N40Q
G-LTBR
DG-LTBR
70KDa
55KDa
GAPDH
37KDa

Figure 7J

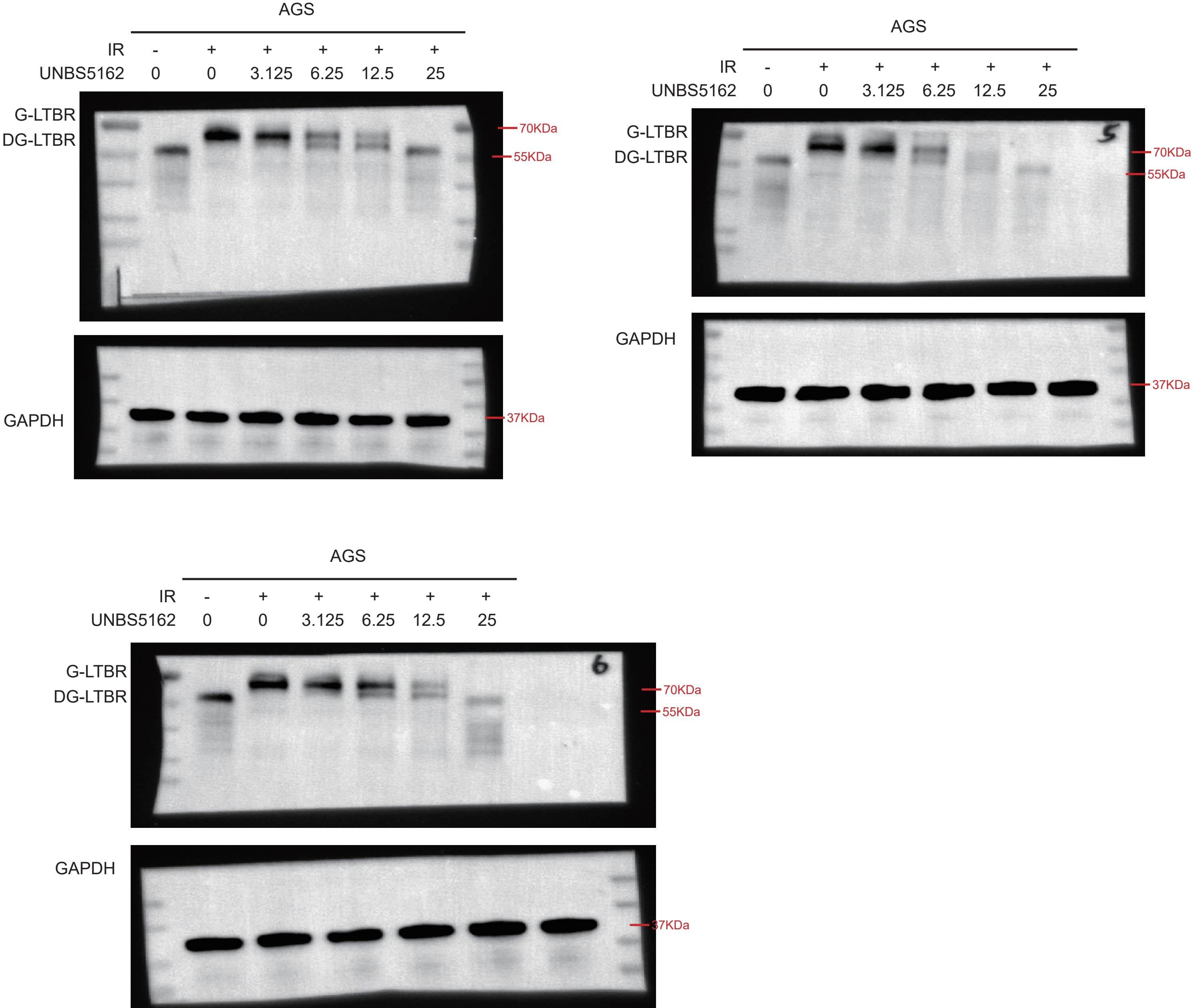

AGS
IR - + + + + +
UNBS5162 0 0 3.125 6.25 12.5 25
G-LTBR
DG-LTBR
70KDa
55KDa
GAPDH
37KDa
AGS
IR - + + + + +
UNBS5162 0 0 3.125 6.25 12.5 25
G-LTBR
DG-LTBR
5
70KDa
55KDa
GAPDH
37KDa
AGS
IR - + + + + +
UNBS5162 0 0 3.125 6.25 12.5 25
G-LTBR
DG-LTBR
6
70KDa
55KDa
GAPDH
37KDa

Figure S1

H

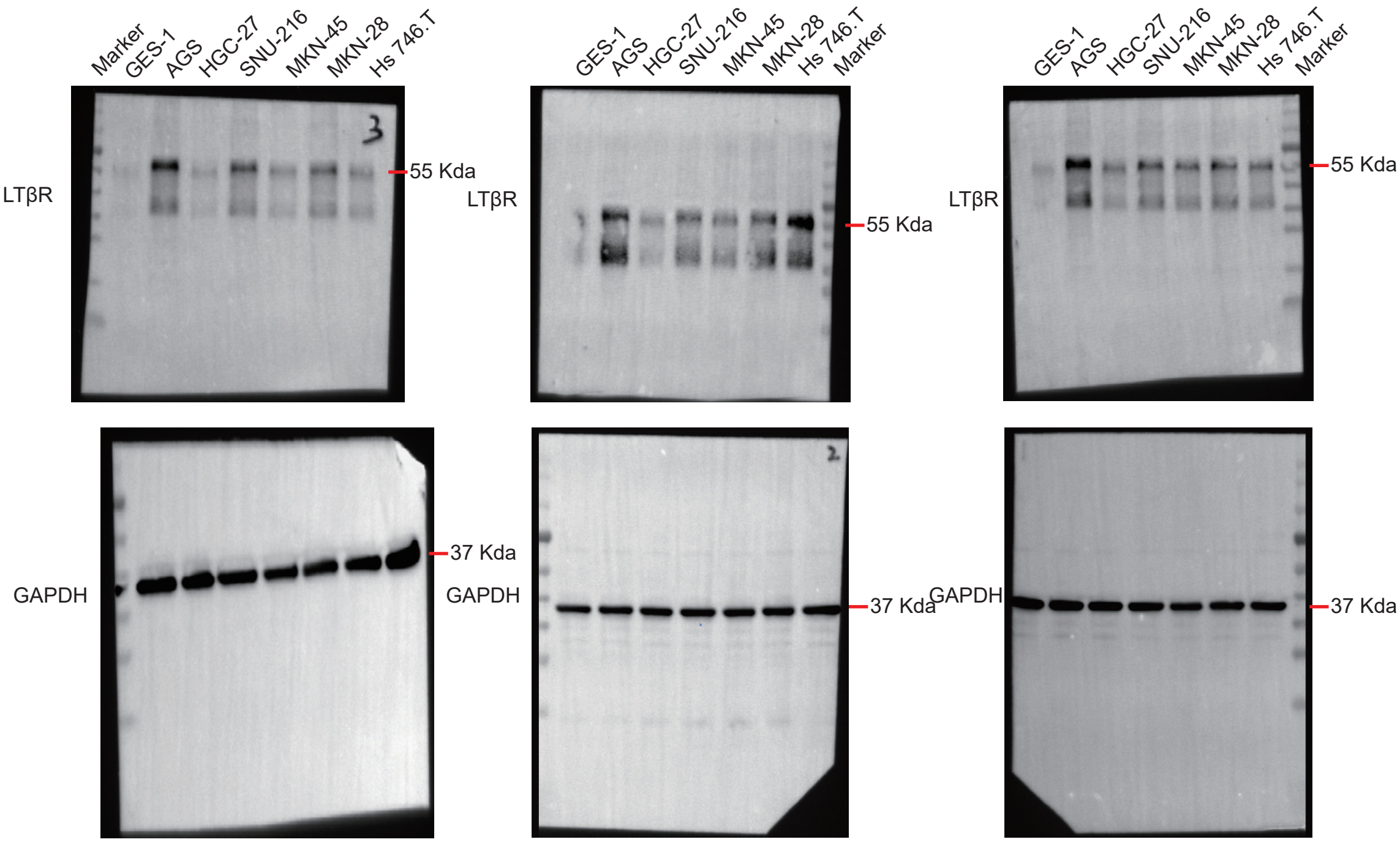
Marker
GES-1
AGS
HGC-27
SNU-216
MKN-45
MKN-28
Hs 746.T
LTβR
55 Kda
GAPDH
37 Kda

J

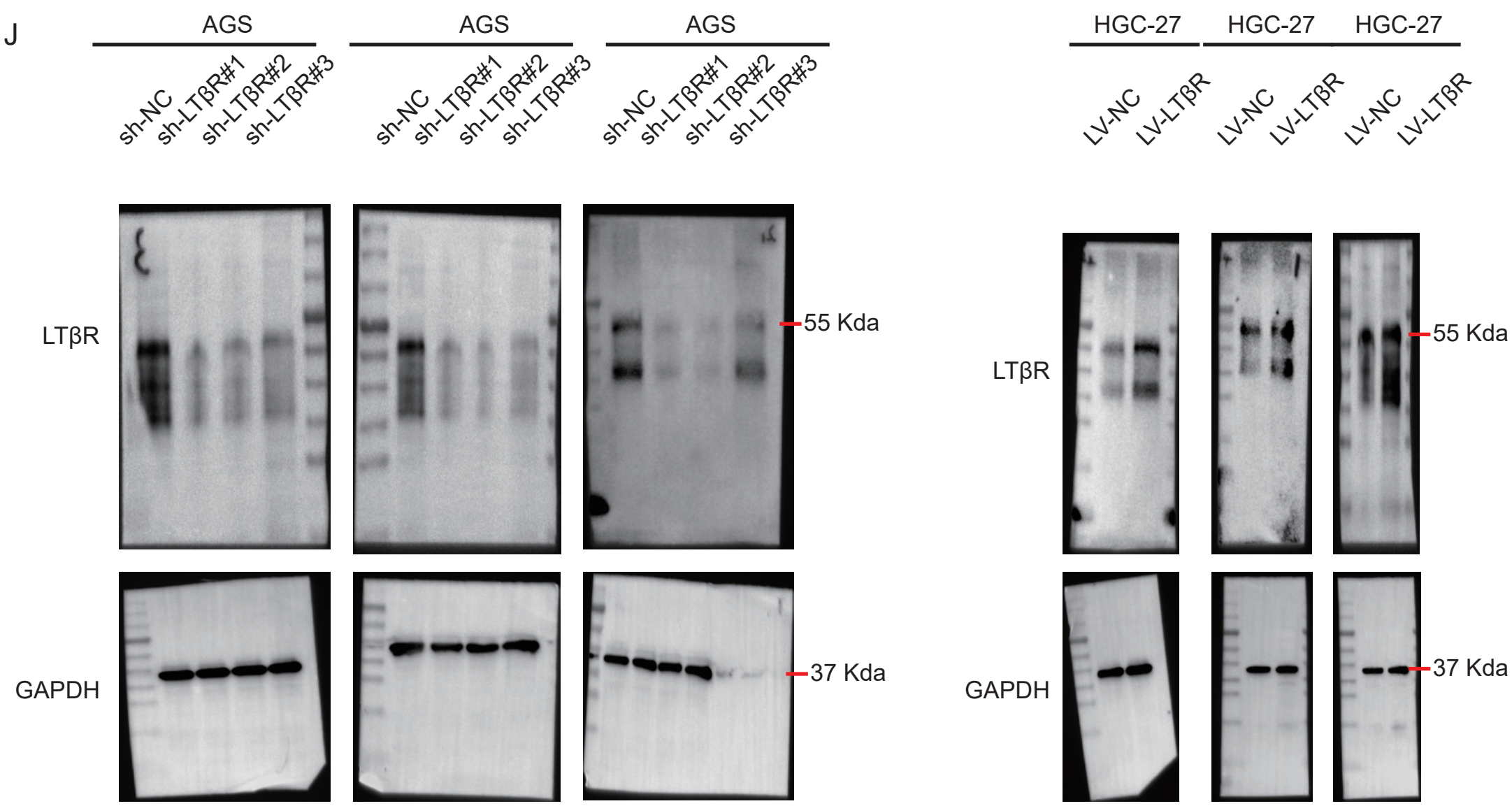
AGS
sh-NC
sh-LTβR#1
sh-LTβR#2
sh-LTβR#3
HGC-27
LV-NC
LV-LTβR
LTβR
55 Kda
GAPDH
37 Kda

Figure S3A

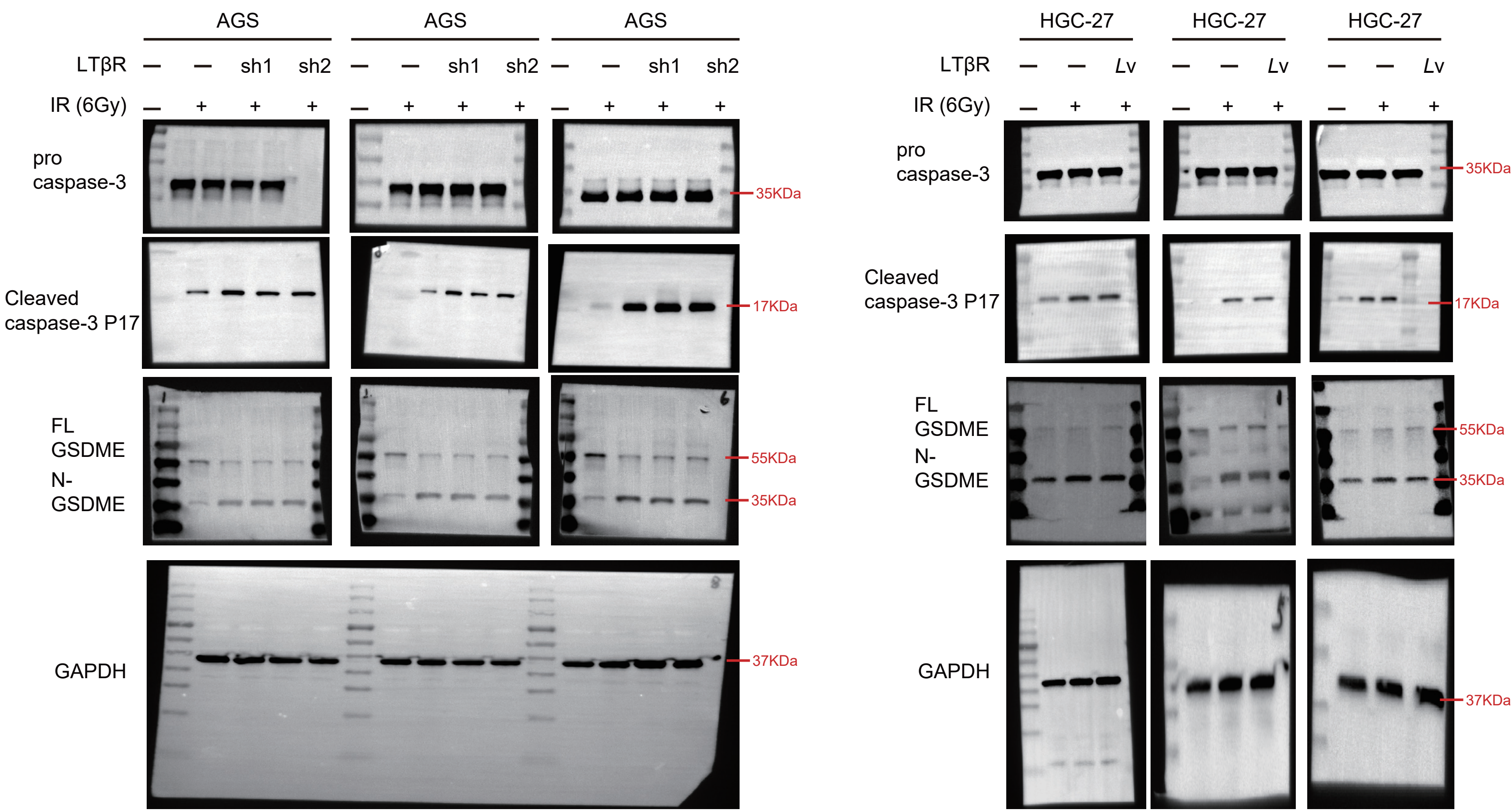
AGS
AGS
AGS
HGC-27
HGC-27
HGC-27
LTβR
— — sh1 sh2
— — Lv
IR (6Gy)
— + + +
— + +
pro caspase-3
Cleaved caspase-3 P17
FL GSDME
N-GSDME
GAPDH
35KDa
17KDa
55KDa
35KDa
37KDa

Figure S5H

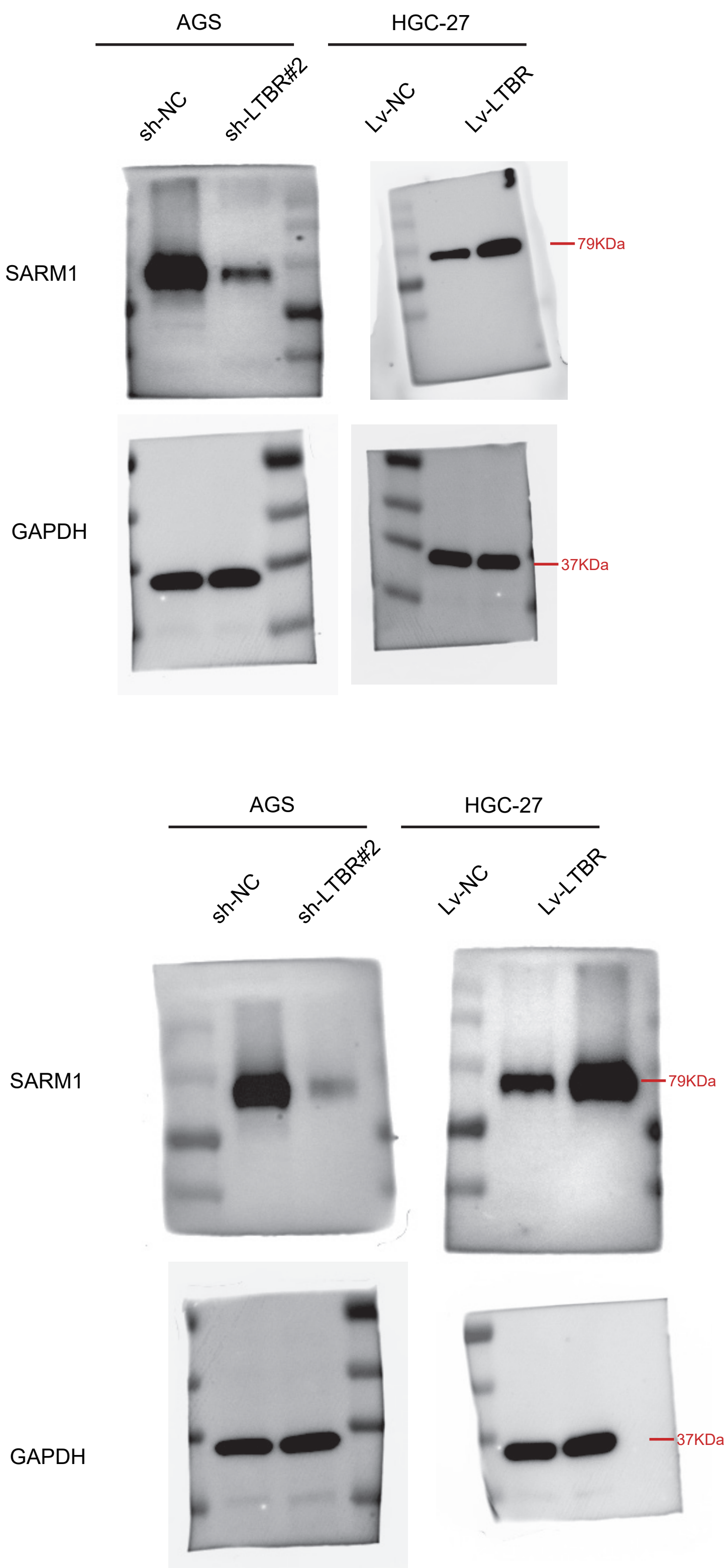

AGS
HGC-27
sh-NC
sh-LTBR#2
Lv-NC
Lv-LTBR
SARM1
79KDa
GAPDH
37KDa
AGS
HGC-27
sh-NC
sh-LTBR#2
Lv-NC
Lv-LTBR
SARM1
79KDa
GAPDH
37KDa
AGS
HGC-27
sh-NC
sh-LTBR#2
Lv-NC
Lv-LTBR
SARM1
79KDa
GAPDH
37KDa

Figure S9F

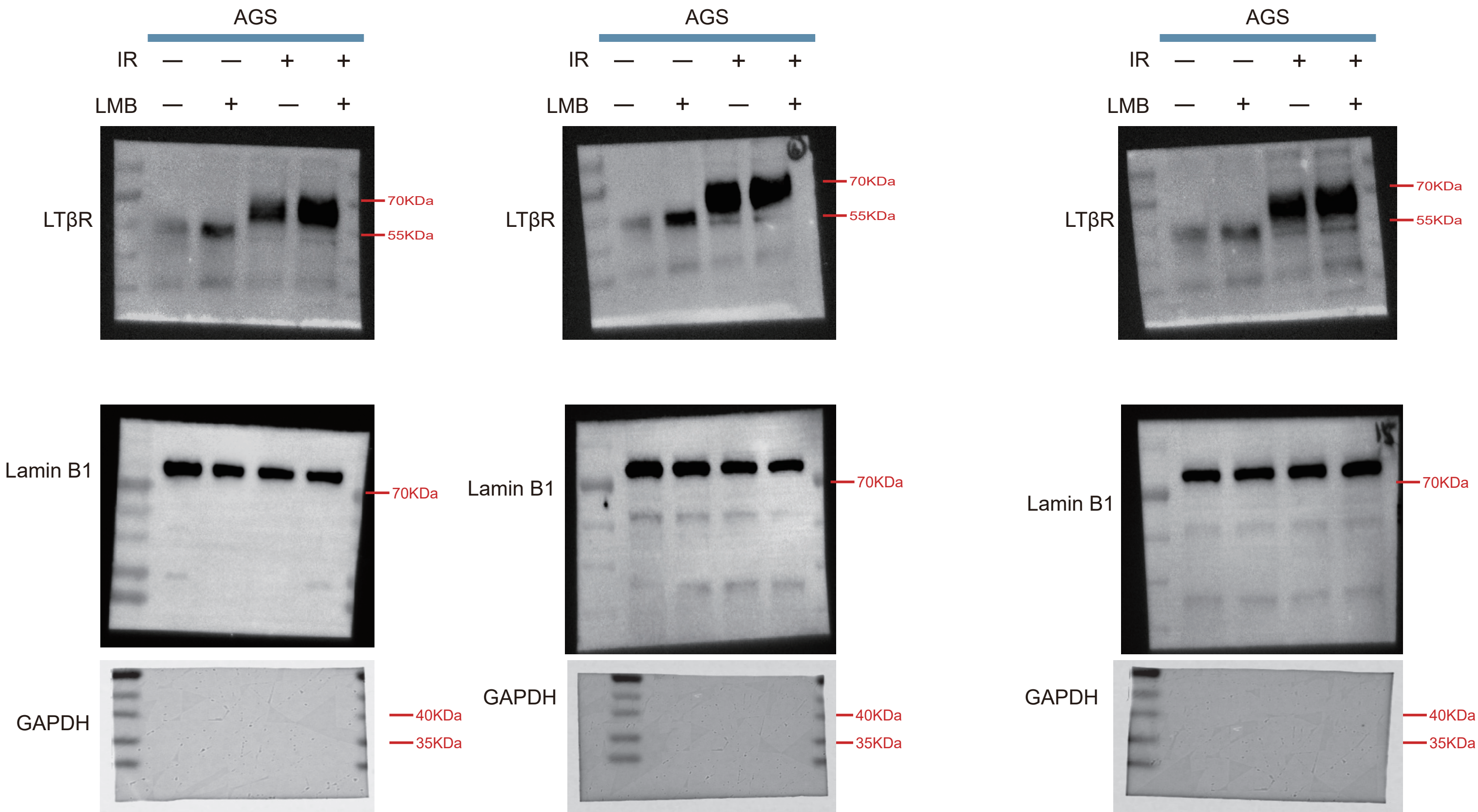

Figure S10G

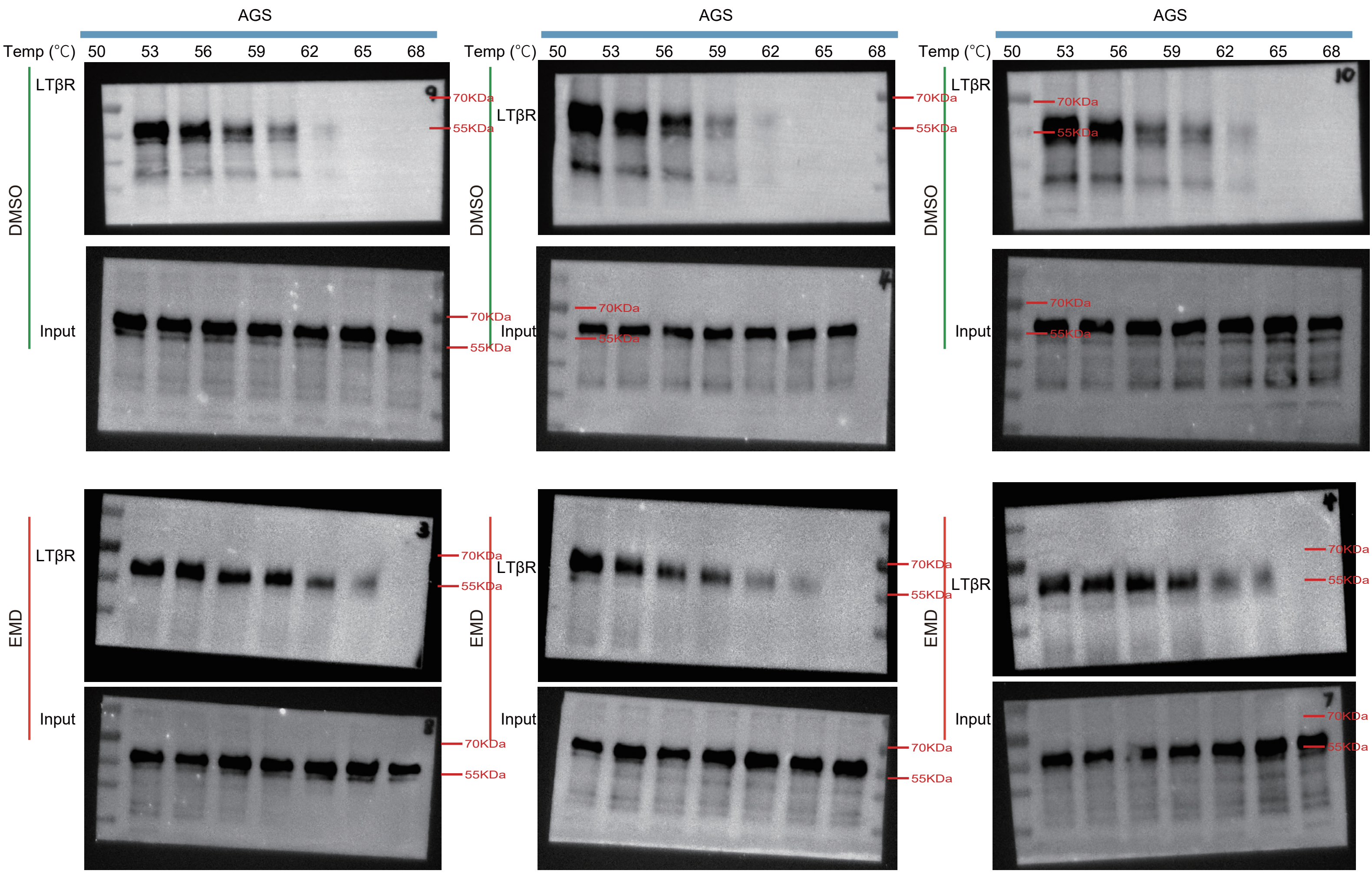

Figure S9H

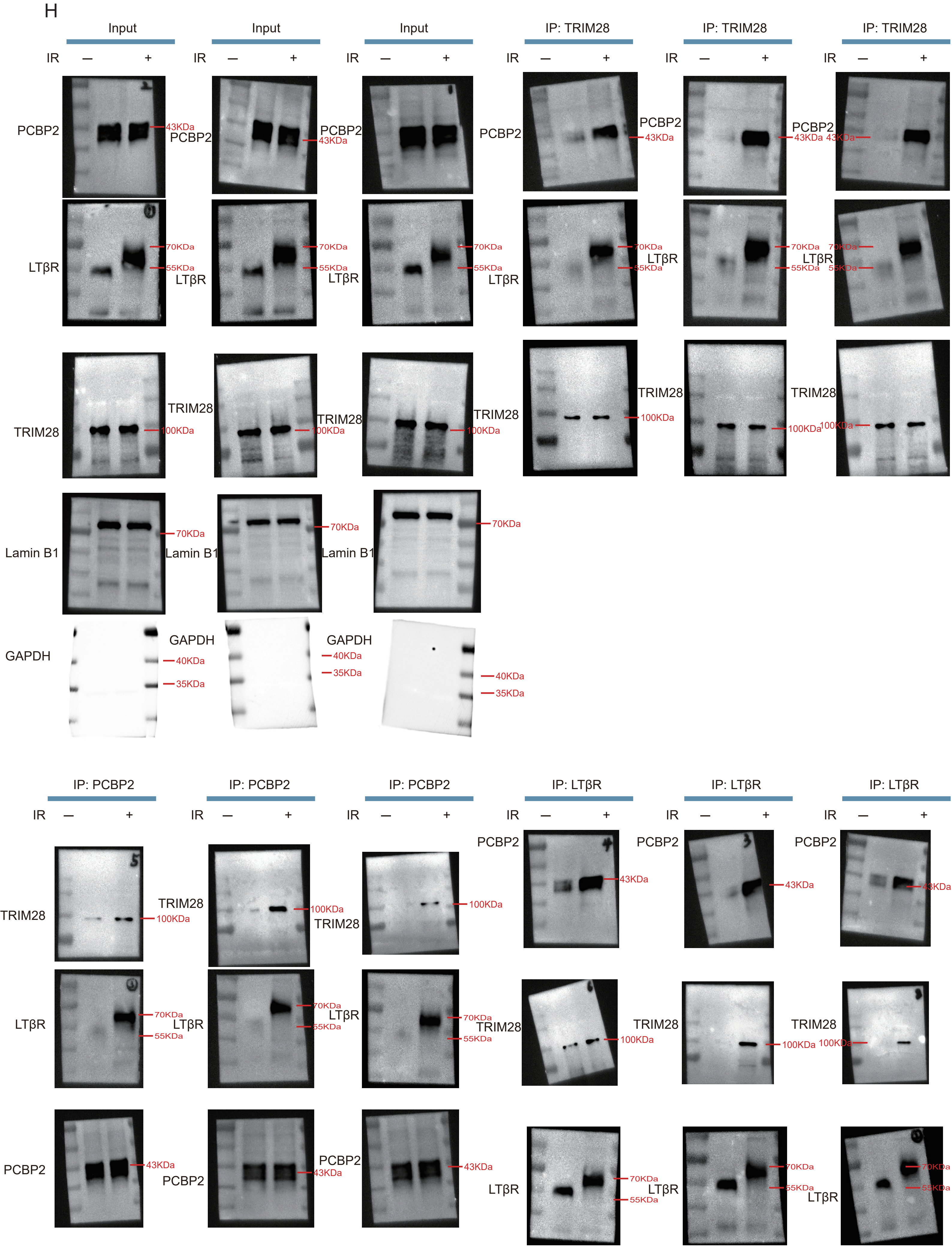
H
Input
Input
Input
IP: TRIM28
IP: TRIM28
IP: TRIM28
IR – +
PCBP2
43KDa
LTβR
70KDa
55KDa
TRIM28
100KDa
Lamin B1
70KDa
GAPDH
40KDa
35KDa
IP: PCBP2
IP: PCBP2
IP: PCBP2
IP: LTβR
IP: LTβR
IP: LTβR
IR – +
TRIM28
100KDa
LTβR
70KDa
55KDa
PCBP2
43KDa

Figure S10A+B

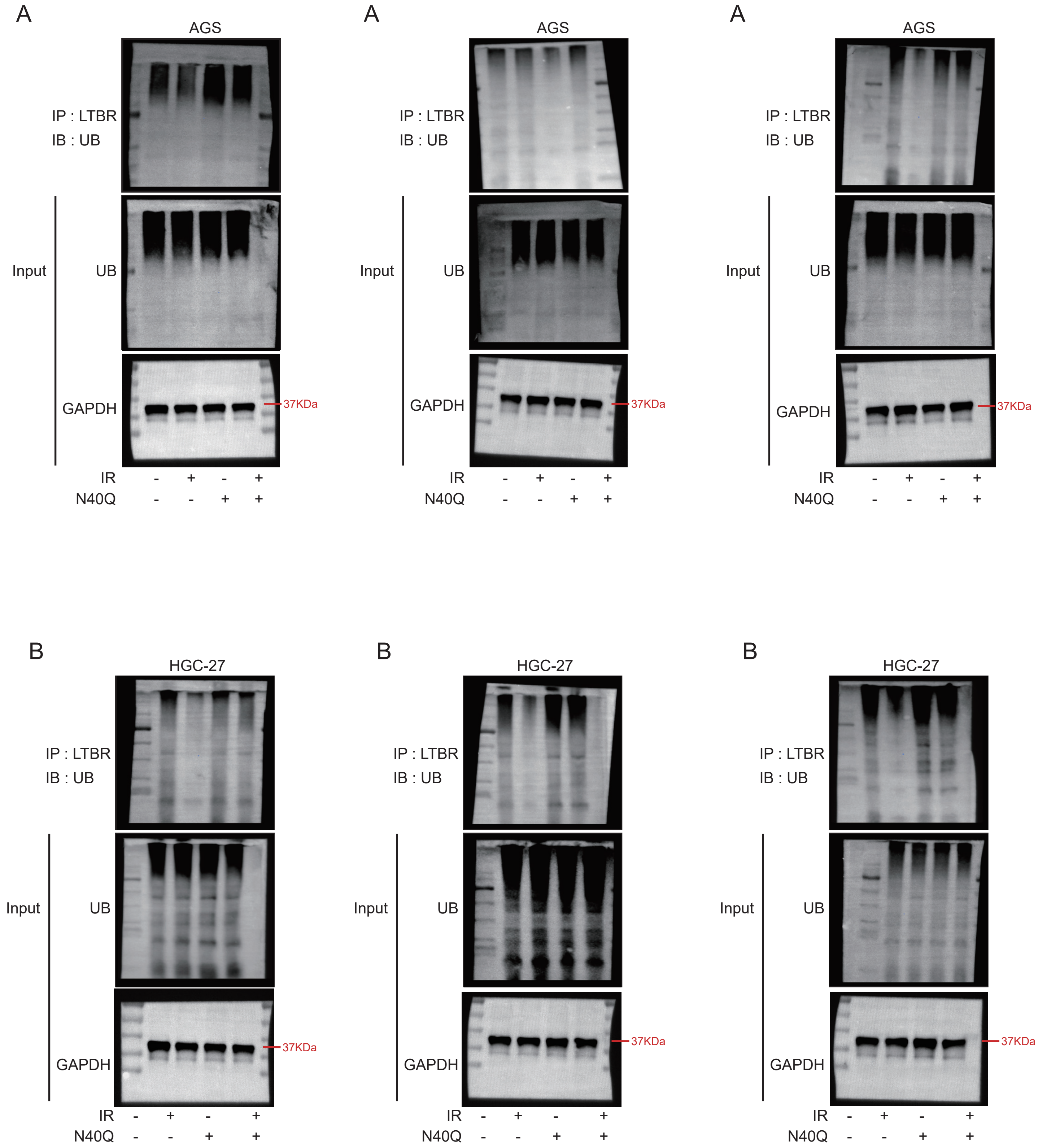

Figure S11A

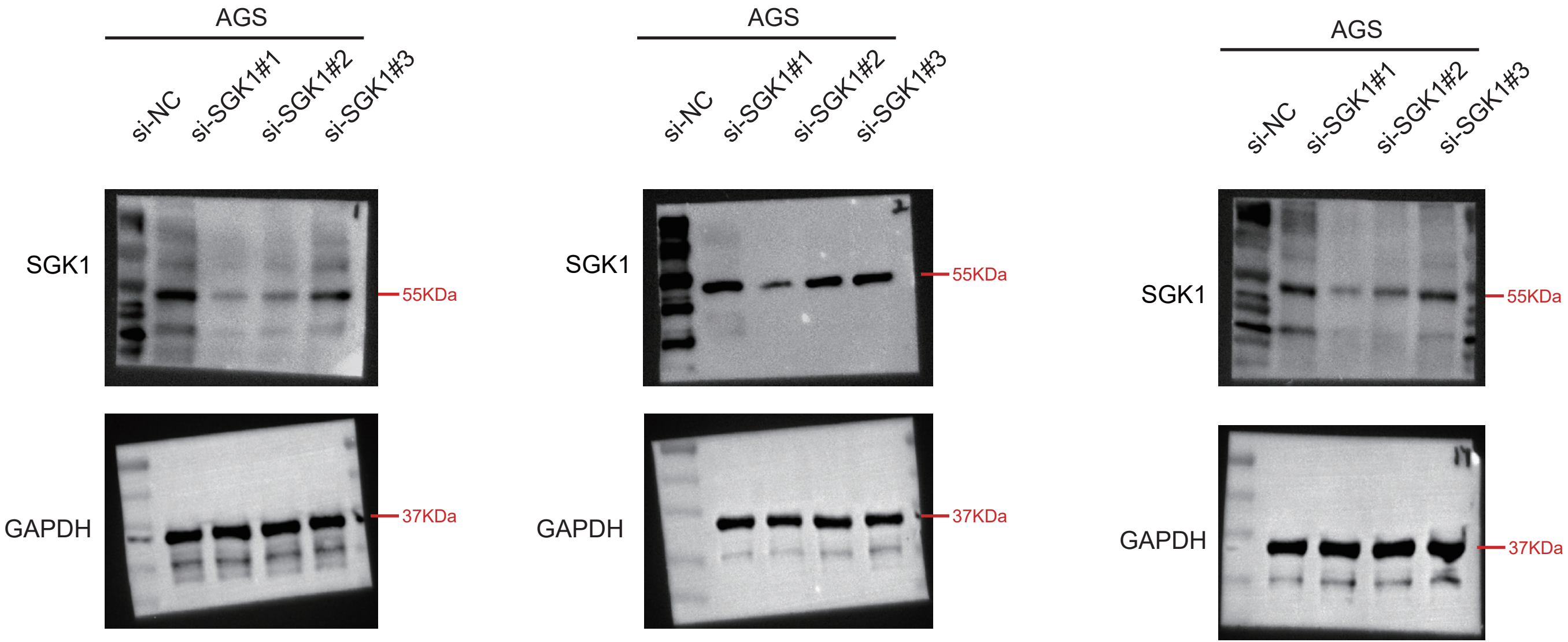
AGS
si-NC
si-SGK1#1
si-SGK1#2
si-SGK1#3
SGK1
55KDa
GAPDH
37KDa
AGS
si-NC
si-SGK1#1
si-SGK1#2
si-SGK1#3
SGK1
55KDa
GAPDH
37KDa
AGS
si-NC
si-SGK1#1
si-SGK1#2
si-SGK1#3
SGK1
55KDa
GAPDH
37KDa
