# Supplementary material for: Radiation‐Induced Tumor‐Intrinsic LTβR N‐Glycosylation Suppresses Pyroptosis Through TRIM28‐Mediated PCBP2 SUMOylation to Promote Gastric Cancer Radioresistance
Source: Adv Sci (Weinh). 2026 Jun 22:e76157. Online ahead of print. doi: 10.1002/advs.76157 (PMC13336884; doi:10.1002/advs.76157)
Supplement: Supplementary file 1 — Supporting File 1: advs76157‐sup‐0001‐SuppMat.docx. [file ADVS-9999-e76157-s002.docx]

**Supplementary Figures and Figure legends**

**
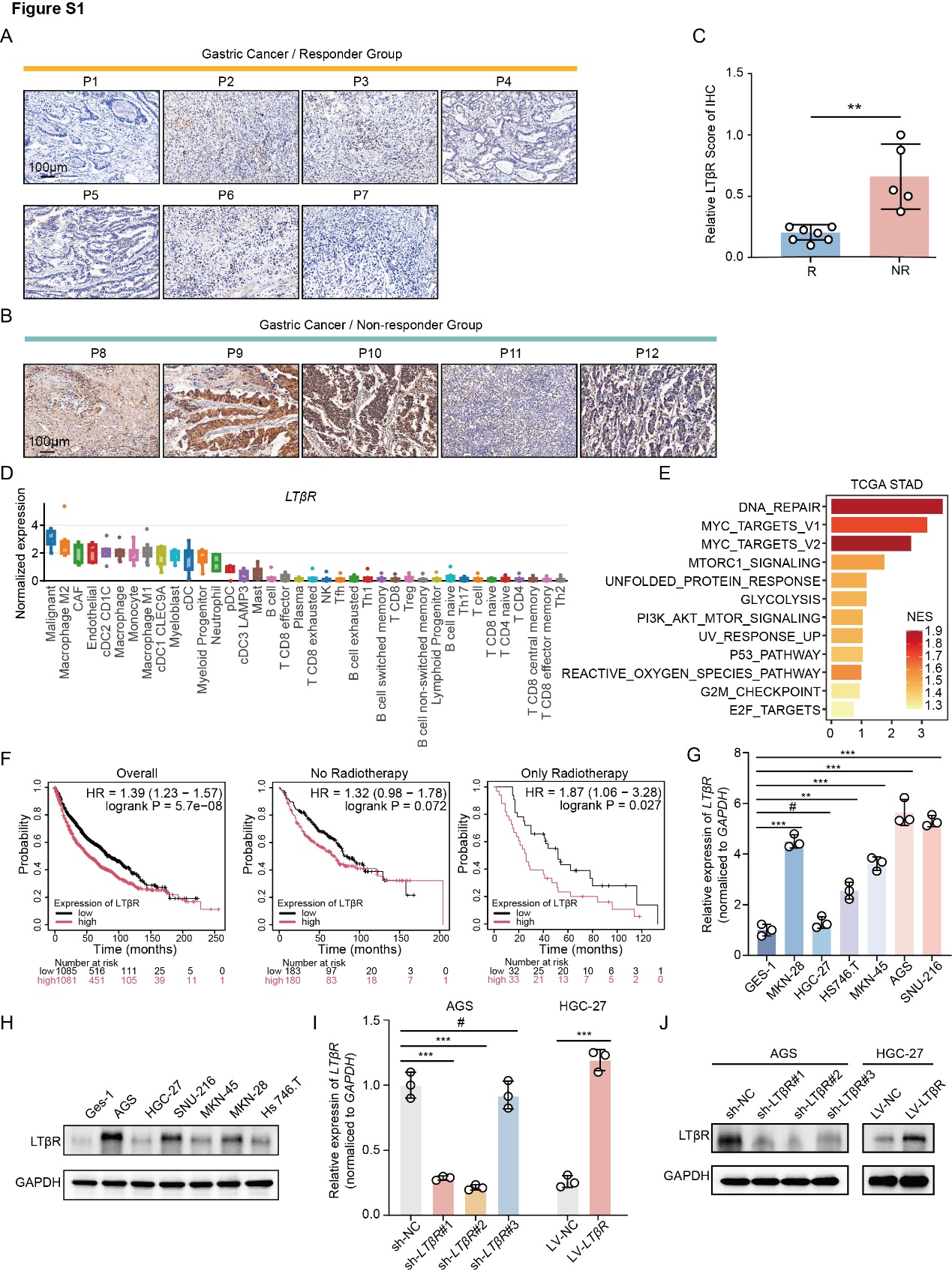
**

**Figure S1.** **Clinical, bioinformatic, and cellular validation of LTβR expression in GC radioresistance (Related to Figure 1).** (A, B) Representative IHC images of LTβR staining in GC tissues from responder (R, n = 7) (A) and non-responder (NR, n = 5) groups (B). Scale bar, 100 μm. (C) Quantification of relative LTβR IHC score in responder and non-responder groups. (D) UMAP visualization of pan-cancer single-cell RNA-seq data showing *LTβR* expression across different cell types from CIDE database. (E) GSEA analysis of Hallmark gene sets in *LTβR*-high vs. *LTβR*-low groups from the TCGA-STAD cohort. (F) Kaplan-Meier survival curves of *LTβR* expression in lung cancer patients from the KMplot database, stratified by the overall cohort, the no radiotherapy group, and the radiotherapy-only group. (G, H) qRT-PCR (G) and Western blot (H) analysis of LTβR expression in normal gastric epithelial cells (GES-1) and GC cell lines. (I) qRT-PCR analysis of *LTβR* mRNA expression in AGS cells with *LTβR* knockdown and HGC-27 cells with *LTβR* overexpression. (J) Western blot analysis of LTβR expression in AGS cells with *LTβR* knockdown and HGC-27 cells with *LTβR* overexpression. Data are presented as mean ± SD. Statistical significance was determined by one-way ANOVA with Tukey post-test (G, I left), or Student’s t-test (C, I right), or log-rank test for survival analysis (F), **P < 0.01, ***P < 0.001, #P > 0.05.

**
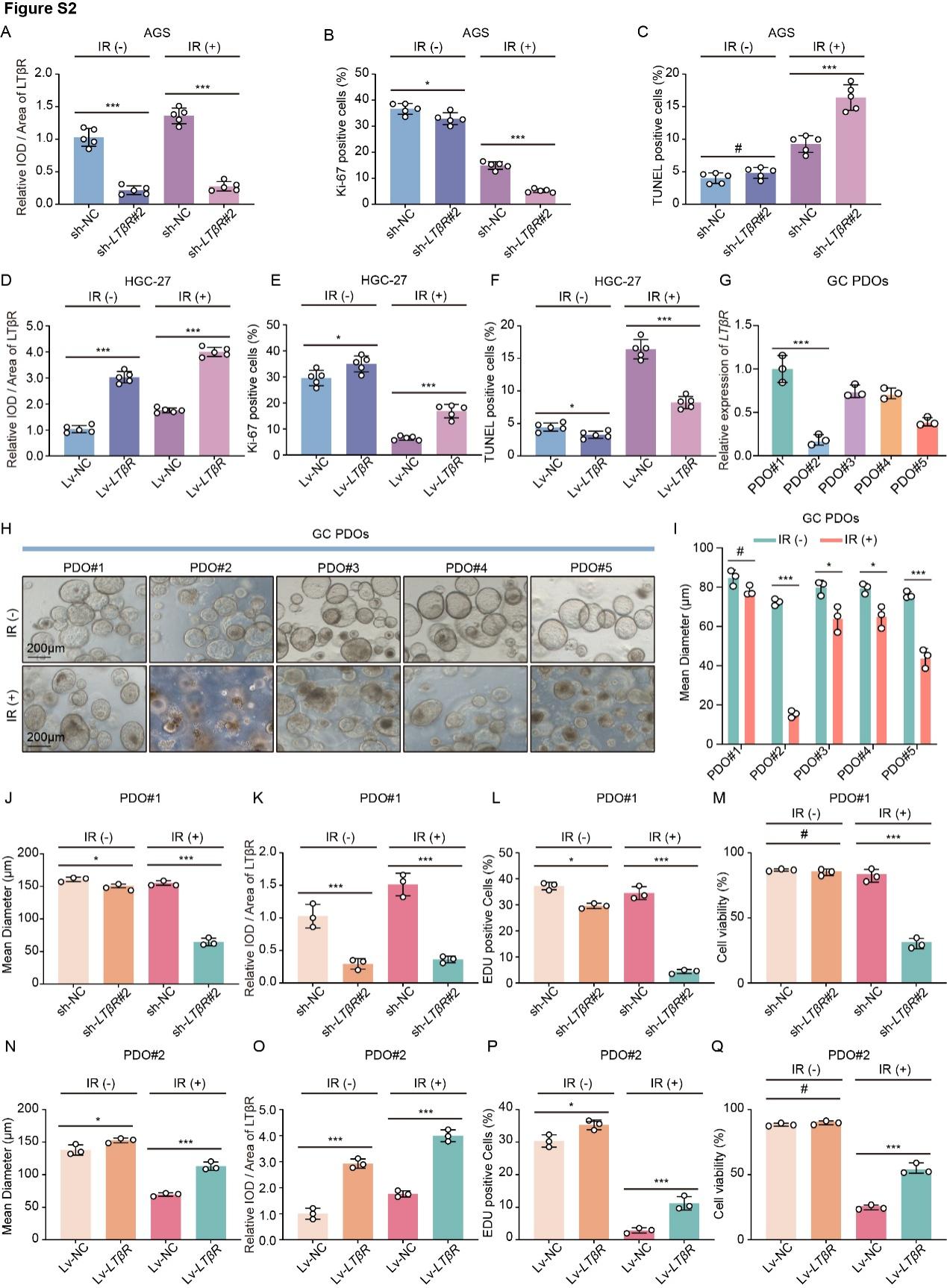
**

**Figure S2. Quantification of xenograft and PDO responses to LTβR modulation under IR (Related to Figure 2).** (A–C) Quantification of relative integrated optical density (IOD)/area of LTβR (A), Ki-67-positive cells (B), and TUNEL-positive cells (C) in AGS xenografts with *LTβR* knockdown or control, with or without IR. (D–F) Quantification of relative IOD/area of LTβR (D), Ki-67-positive cells (E), and TUNEL-positive cells (F) in HGC-27 xenografts with *LTβR* overexpression or control, with or without IR. (G) qRT-PCR analysis of basal *LTβR* mRNA expression in five patient-derived GC organoid lines (PDO#1–PDO#5). (H, I) Representative bright-field images of PDO#1 and PDO#2 organoids with or without 6 Gy IR (H), and quantification of mean organoid diameter (I). Scale bar, 200 μm. (J–M) Quantification of mean diameter (J), relative IOD/area of LTβR (K), EdU-positive cells (L), and cell viability (M) in PDO#1 organoids with *LTβR* knockdown or control, with or without 6 Gy IR. (N–Q) Quantification of mean diameter (N), relative IOD/area of LTβR (O), EdU-positive cells (P), and cell viability (Q) in PDO#2 organoids with LTβR overexpression or control, with or without 6 Gy IR. Data are presented as mean ± SD. Statistical significance was determined by two-way ANOVA followed by Bonferroni’s multiple comparisons test (A–F, J–Q), or one-way ANOVA with Tukey post-test (G), or Student’s t-test (I), *P < 0.05, ***P < 0.001, #P > 0.05.

**
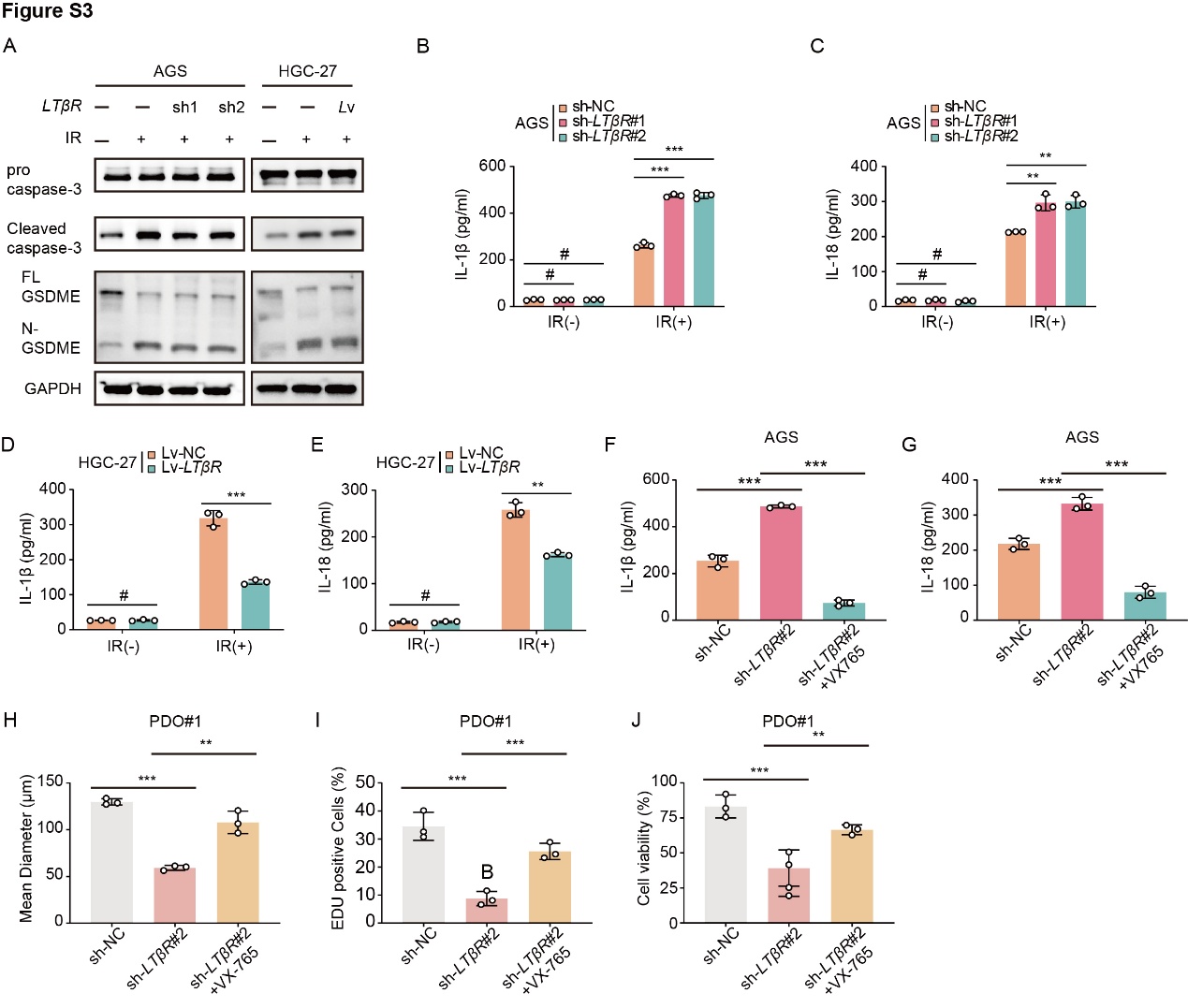
**

**Figure S3. Supporting analyses of pyroptosis-associated pathways regulated by LTβR under IR (Related to Figure 3).** (A) Western blot analysis of pro-caspase-3, cleaved caspase-3, FL-GSDME, and N-GSDME in the indicated cells with or without 6 Gy IR. (B–C) IL-1β and IL-18 levels in AGS cells transfected with sh-NC or sh-LTβR (#1, #2) with or without IR treatment. (D–E) IL-1β and IL-18 levels in HGC-27 cells transfected with Lv-NC or Lv-*LTβR* with or without IR treatment. (F–G) IL-1β and IL-18 levels in AGS cells treated with sh-NC, sh-*LTβR*#2, or sh-LTβR#2 plus VX-765. (H–J) Quantification of mean diameter (H), EdU-positive cells (I) and cell viability (J) in PDO#1 organoids from the indicated groups after 6 Gy IR. Data are presented as mean ± SD. Statistical significance was determined by two-way ANOVA followed by Bonferroni’s multiple comparisons test (B–E), or one-way ANOVA with Tukey post-test (F–J), **P < 0.01, ***P < 0.001, #P > 0.05.

**
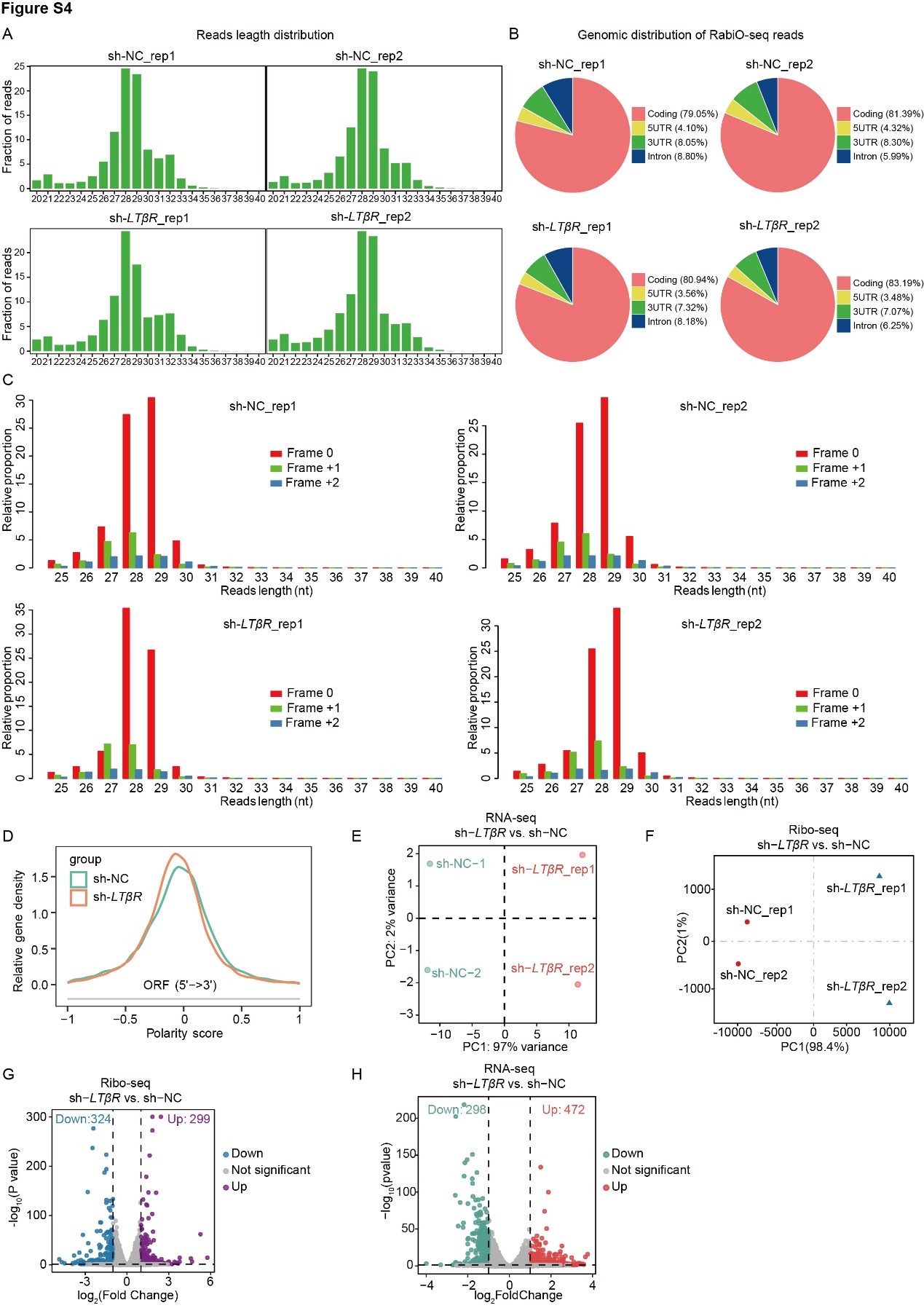
**

**Figure S4. Quality Control of Ribo-seq and RNA-seq Data (Related to Figure 4).** (A) Read length distribution of Ribo-seq data from sh-NC and sh-*LTβR* AGS cells. (B) Genomic distribution of Ribo-seq reads from sh-NC and sh-*LTβR* AGS cells. (C) Frame distribution of Ribo-seq reads from sh-NC and sh-*LTβR* AGS cells. (D) Polarity score distribution of Ribo-seq reads across ORFs in sh-NC and sh-*LTβR* AGS cells. (E-F) PCA of RNA-seq (E) and Ribo-seq (F) data from sh-NC and sh-*LTβR* AGS cells. (G–H) Volcano plots of differentially expressed genes in Ribo-seq (G) and RNA-seq (H) data between sh-NC and sh-*LTβR* AGS cells.

**
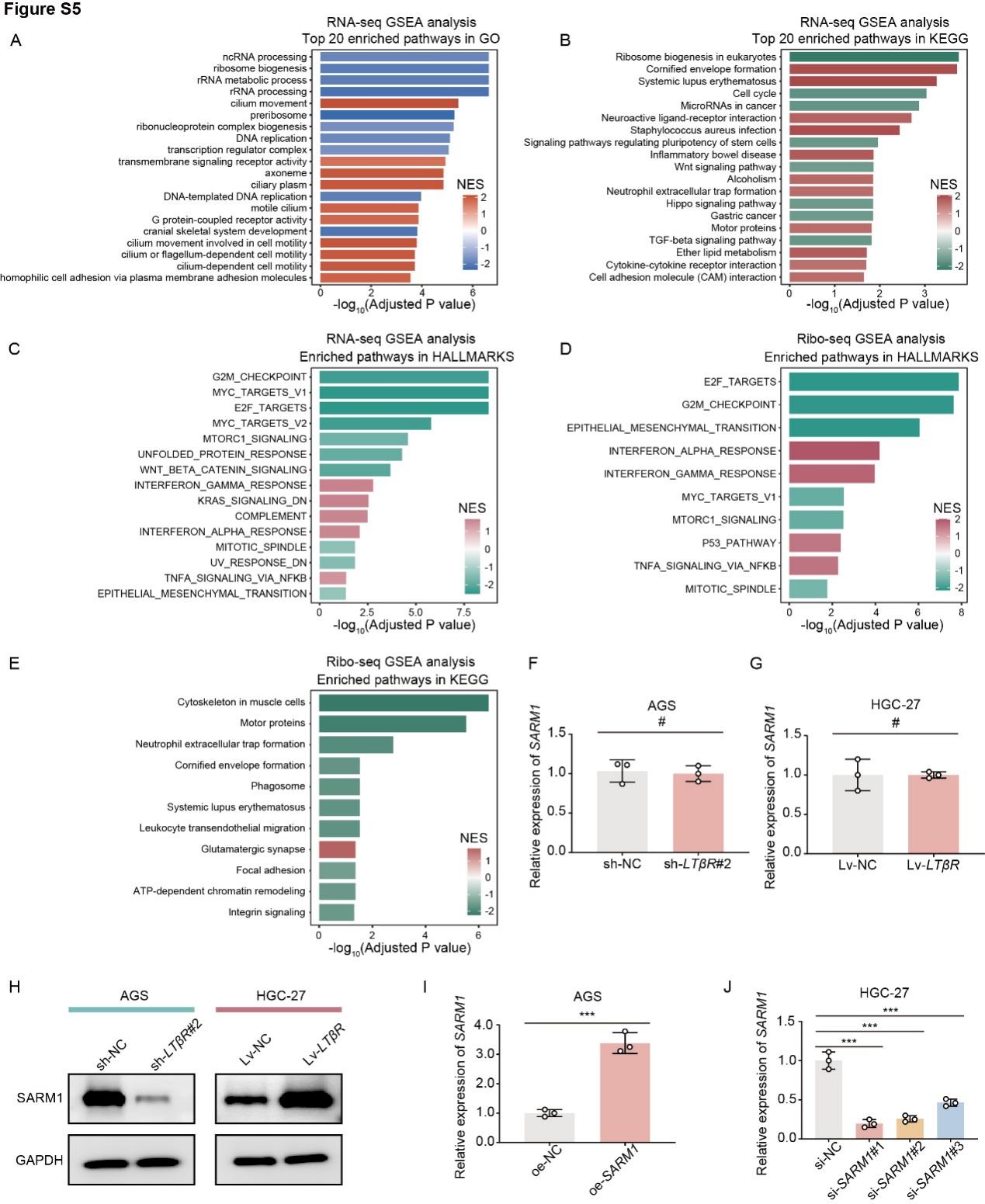
**

**Figure S5. Multi-omics enrichment analyses and validation of SARM1 as an LTβR-regulated translational target (Related to Figure 4).** (A–E) GSEA analysis of top 20 enriched GO (A), KEGG (B), and Hallmark (C) pathways in RNA-seq GSEA analysis, and enriched Hallmark (D) and KEGG (E) pathways in Ribo-seq GSEA analysis of sh-*LTβR* vs. sh-NC AGS cells. (F, G) qRT-PCR analysis of *SARM1* mRNA expression in AGS cells with *LTβR* knockdown (F) and HGC-27 cells with *LTβR* overexpression (G). (H) Western blot analysis of SARM1 expression in AGS cells with *LTβR* knockdown and HGC-27 cells with *LTβR* overexpression. (I, J) qRT-PCR analysis of *SARM1* mRNA expression in AGS cells with *SARM1* overexpression (I) and HGC-27 cells with *SARM1* knockdown (J). Data are presented as mean ± SD. Statistical significance was determined by Student’s t-test (F, G, I), or one-way ANOVA with Tukey post-test (J), ***P < 0.001, #P > 0.05.

**
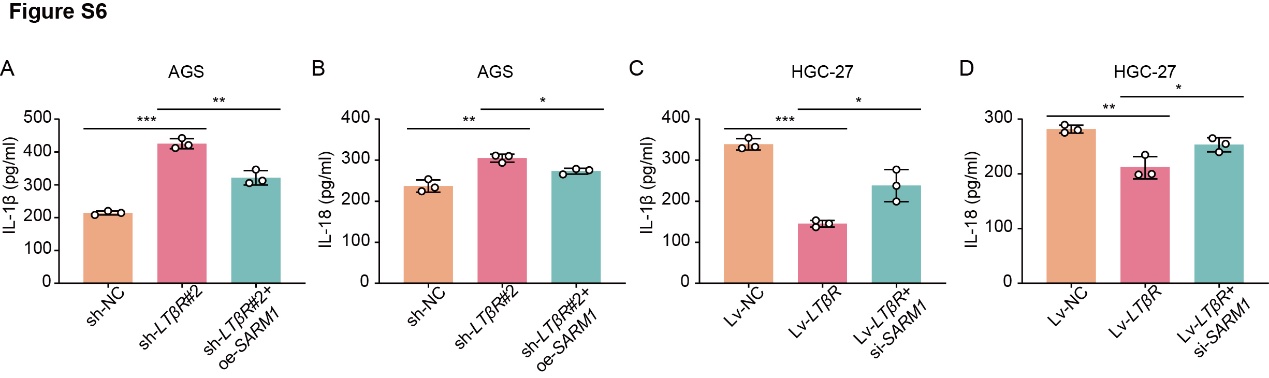
**

**Figure S6. SARM1 mediates LTβR-dependent regulation of IL-1β and IL-18 release under IR (Related to Figure 4).** (A, B) IL-1β and IL-18 levels in AGS cells transfected with sh-NC, sh-*LTβR* #2, or sh-*LTβR* #2 plus oe-*SARM1* following 6 Gy IR. (C, D) IL-1β and IL-18 levels in HGC-27 cells transfected with Lv-NC, Lv-*LTβR*, or Lv-*LTβR* plus si-*SARM1* following 6 Gy IR. Data are presented as mean ± SD. Statistical significance was determined by one-way ANOVA with Tukey post-test. *P < 0.05, **P < 0.01, ***P < 0.001.


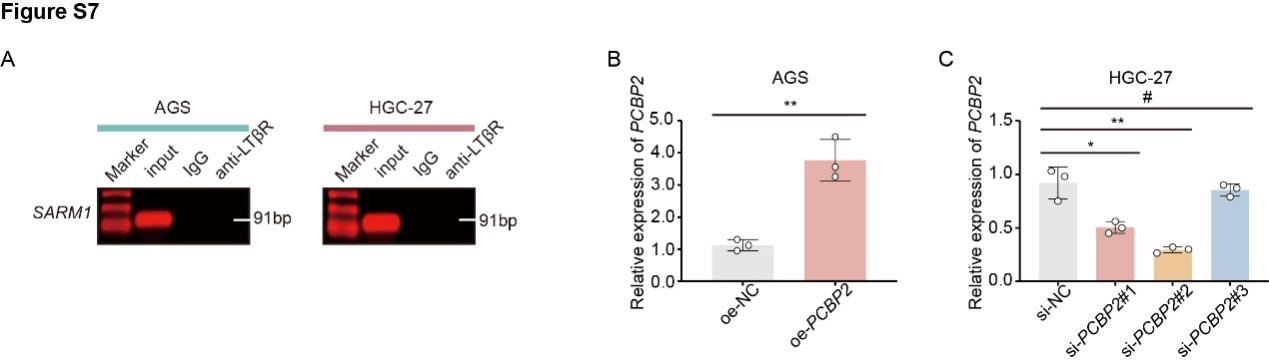


**Figure S7. LTβR does not detectably associate with SARM1 mRNA, whereas PCBP2 supports LTβR-dependent SARM1 translation (Related to Figure 5).** (A) RIP assay showing that LTβR does not directly bind *SARM1* mRNA. (B, C) qRT-PCR analysis of *PCBP2* mRNA expression in AGS cells with *PCBP2* overexpression (B) and HGC-27 cells with *PCBP2* knockdown (C). Data are presented as mean ± SD. Statistical significance was determined by Student’s t-test (B), or one-way ANOVA with Tukey post-test (C), *P < 0.05, ***P < 0.001, #P > 0.05.

**
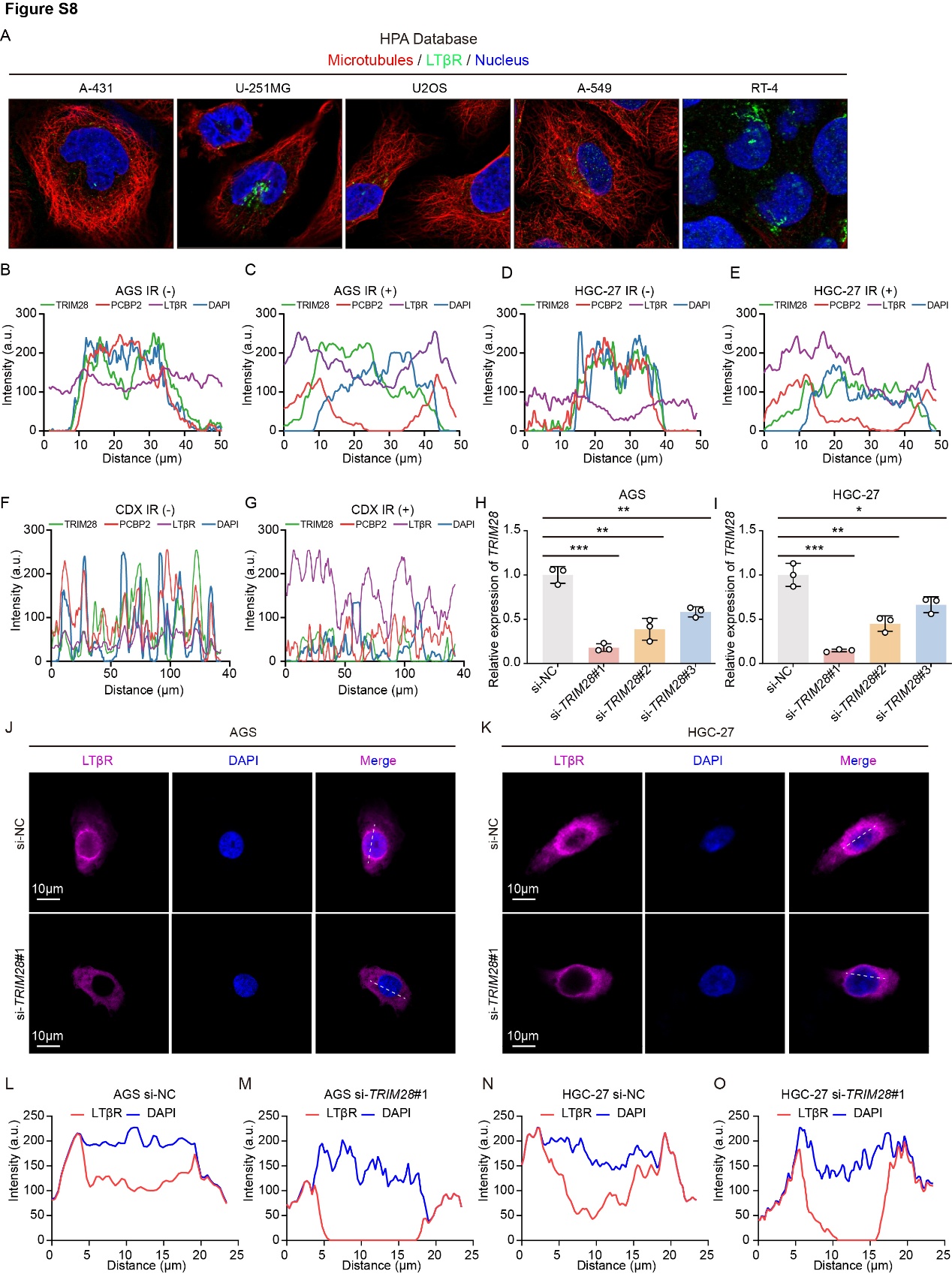
**

**Figure S8. TRIM28 supports nuclear accumulation of LTβR under IR (Related to Figure 6).** (A) Subcellular localization of LTβR in multiple cell lines from the HPA database. (B–G) Line scan analysis of LTβR, TRIM28 and PCBP2 fluorescence intensity along the white lines. (H, I) qRT-PCR analysis of *TRIM28* mRNA expression in AGS cells (H) and HGC-27 cells (I) with *TRIM28* knockdown. (J, K) Representative immunofluorescence images showing localization of LTβR in AGS (J) and HGC-27 (K) cells transfected with or without *TRIM28* knockdown. Nuclei were stained with DAPI (blue). Scale bar, 10 μm. (L–O) Line scan analysis of LTβR fluorescence intensity along the white lines in (J) and (K). Data are presented as mean ± SD. Statistical significance was determined by one-way ANOVA with Tukey post-test (H, I), *P < 0.05, **P < 0.01, ***P < 0.001. Human Protein Atlas: HPA.

**
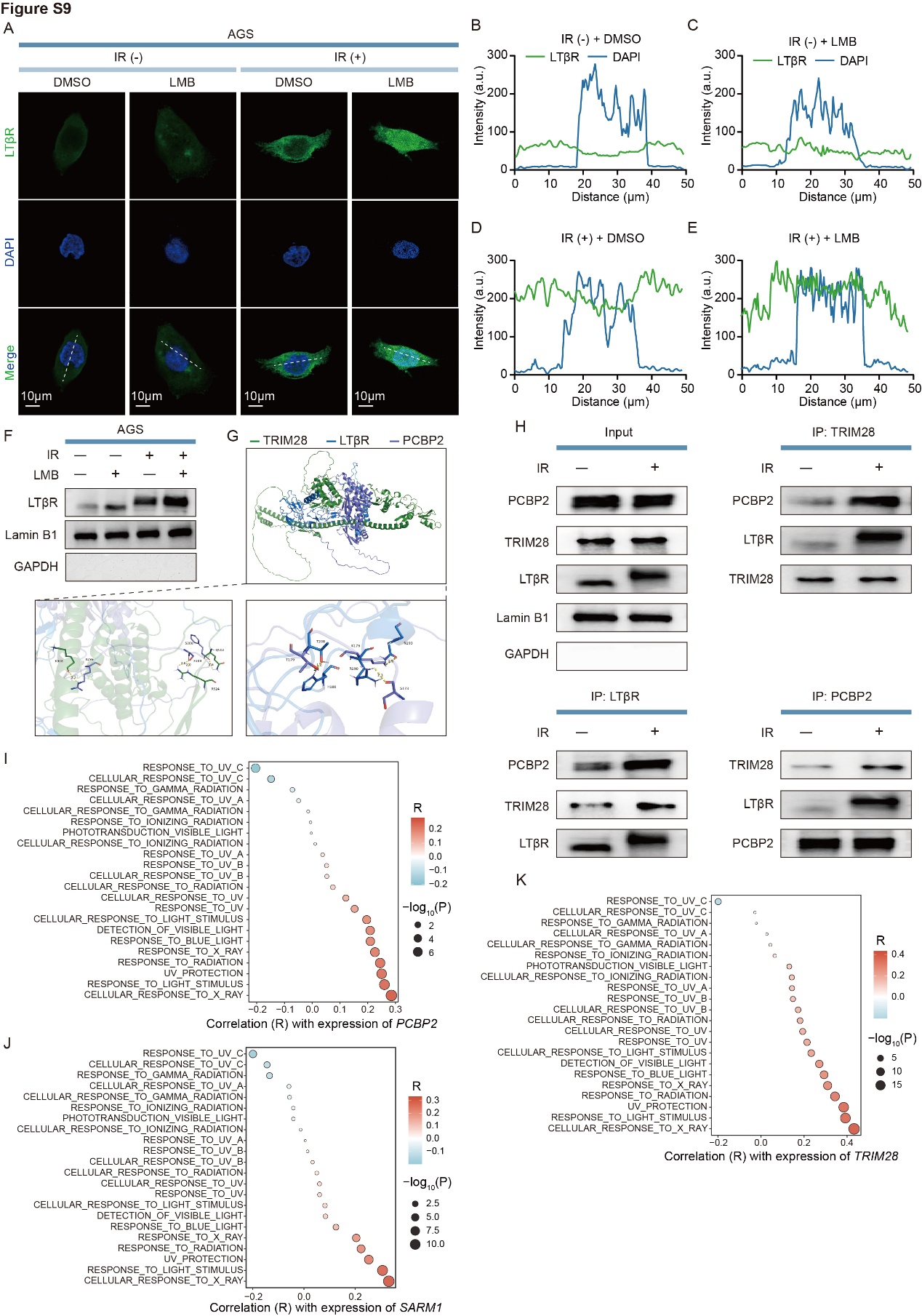
**

**Figure S9. LTβR undergoes regulated nuclear accumulation and associates with PCBP2/TRIM28 under IR (Related to Figure 6).** (A) Immunofluorescence images showing LTβR localization in AGS cells treated with IR (6 Gy), LMB (20 nM, 3 h), or their combination. LTβR is shown in green, and nuclei were stained with DAPI (blue). Scale bar, 10 μm. (B–E) Line-scan analysis of LTβR and DAPI fluorescence intensity along the white dashed lines indicated in (A). (F) Western blot analysis of LTβR abundance in nuclear fractions from AGS cells treated with IR (6 Gy), LMB (20 nM, 3 h), or their combination. Lamin B1 was used as a nuclear fraction marker, and GAPDH was used to assess cytoplasmic contamination. (G) Virtual molecular docking model showing the interaction complex of LTβR, PCBP2, and TRIM28. (H) Co-IP analysis of the interactions among LTβR, PCBP2, and TRIM28 in nuclear extracts of AGS cells treated with or without IR. (I–K) Dot plots showing correlation between *PCBP2* (I), *SARM1* (J), and *TRIM28* (K) expression and radiotherapy-related gene sets ssGSEA score in the TCGA-STAD cohort. Correlation analysis was performed using Pearson correlation analysis.

**
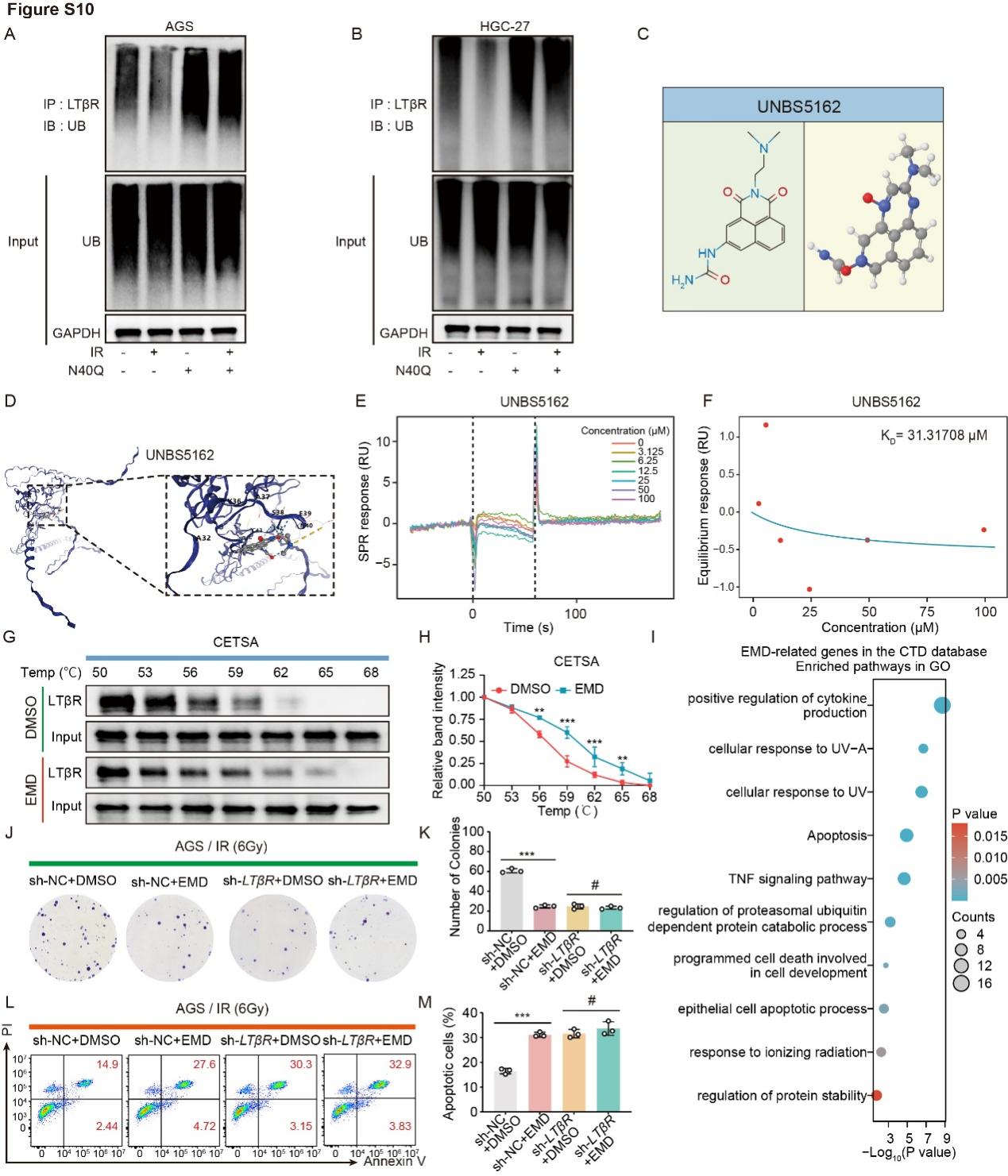
**

**Figure S10. Secondary validation of LTβR-targeting candidates and LTβR dependence of EMD-mediated radiosensitization (Related to Figure 7).** (A, B) Ubiquitination assay showing ubiquitination levels of WT and N40Q mutant LTβR in AGS (A) and HGC-27 (B) cells after 6 Gy IR. Cells were treated with MG132 (20 μM) for 6 h before harvest. (C, D) Chemical structure of UNBS5162 (C) and molecular docking model showing its interaction with the N40 region of LTβR (D). (E and F) SPR sensorgram showing binding kinetics of UNBS5162 to LTβR at 0–100 μM (E) and equilibrium binding curve with calculated KD = 31.32 μM (F). (G, H) CETSA (G) and relative quantification (H) of LTβR thermal stability in AGS cells treated with DMSO or EMD across the indicated temperature range. (I) GO enrichment analysis of EMD target genes from the CTD database. (J, K) Representative images (J) and quantification (K) of colony formation assays in AGS cells following 6 Gy IR treatment with indicated combinations of *LTβR* knockdown and EMD administration. (L, M) Annexin V/PI flow cytometry plots (L) and quantification of apoptotic cells (M) in AGS cells under the specified conditions. Data are presented as mean ± SD. Statistical significance was determined by two-way ANOVA followed by Bonferroni’s multiple comparisons test (H, K, M). ***P < 0.001, #P > 0.05.

**
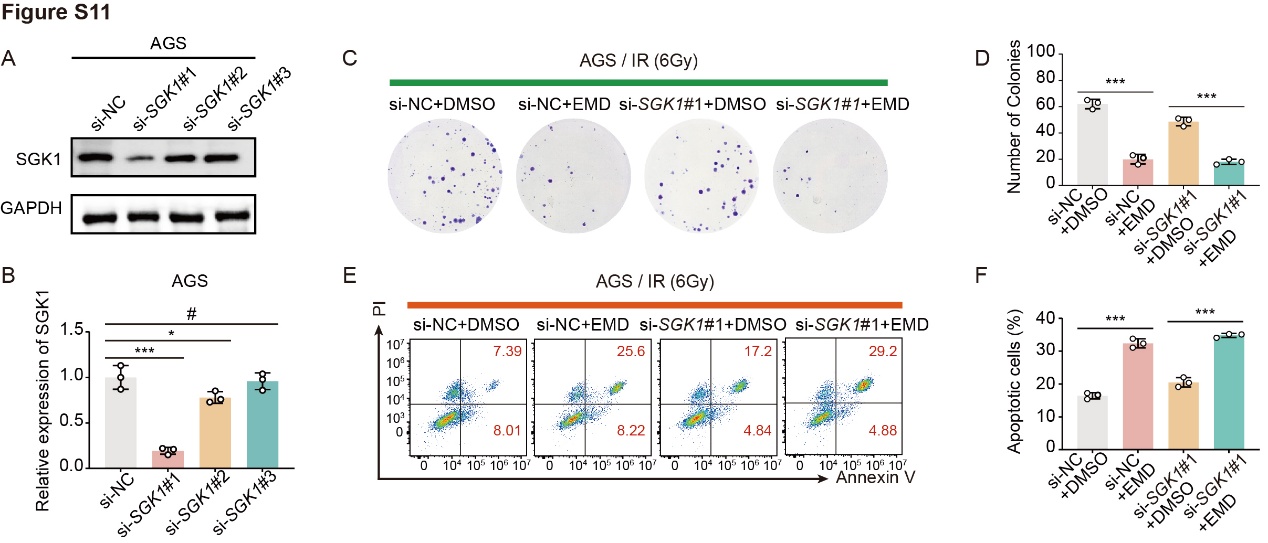
**

**Figure S11. EMD retains radiosensitizing activity in *SGK1*-depleted AGS cells (Related to Figure 7).** (A, B) Western blot (A) and qRT-PCR analysis (B) of SGK1 expression in AGS cells transfected with si-NC or si-*SGK1* (#1, #2, #3). (C, D) Representative images (C) and quantification (D) of colony formation in AGS cells treated with 6 Gy IR with or without 12.5 μM EMD. (E, F) Representative Annexin V/PI flow cytometry plots (E) and quantification of apoptotic cells (F) in AGS cells under indicated conditions, including treatment with or without 12.5 μM EMD. Data are presented as mean ± SD. Statistical significance was determined by one-way ANOVA with Tukey post-test (B), or two-way ANOVA followed by Bonferroni’s multiple comparisons test (D, F). *P < 0.05, ***P < 0.001, #P > 0.05.

**
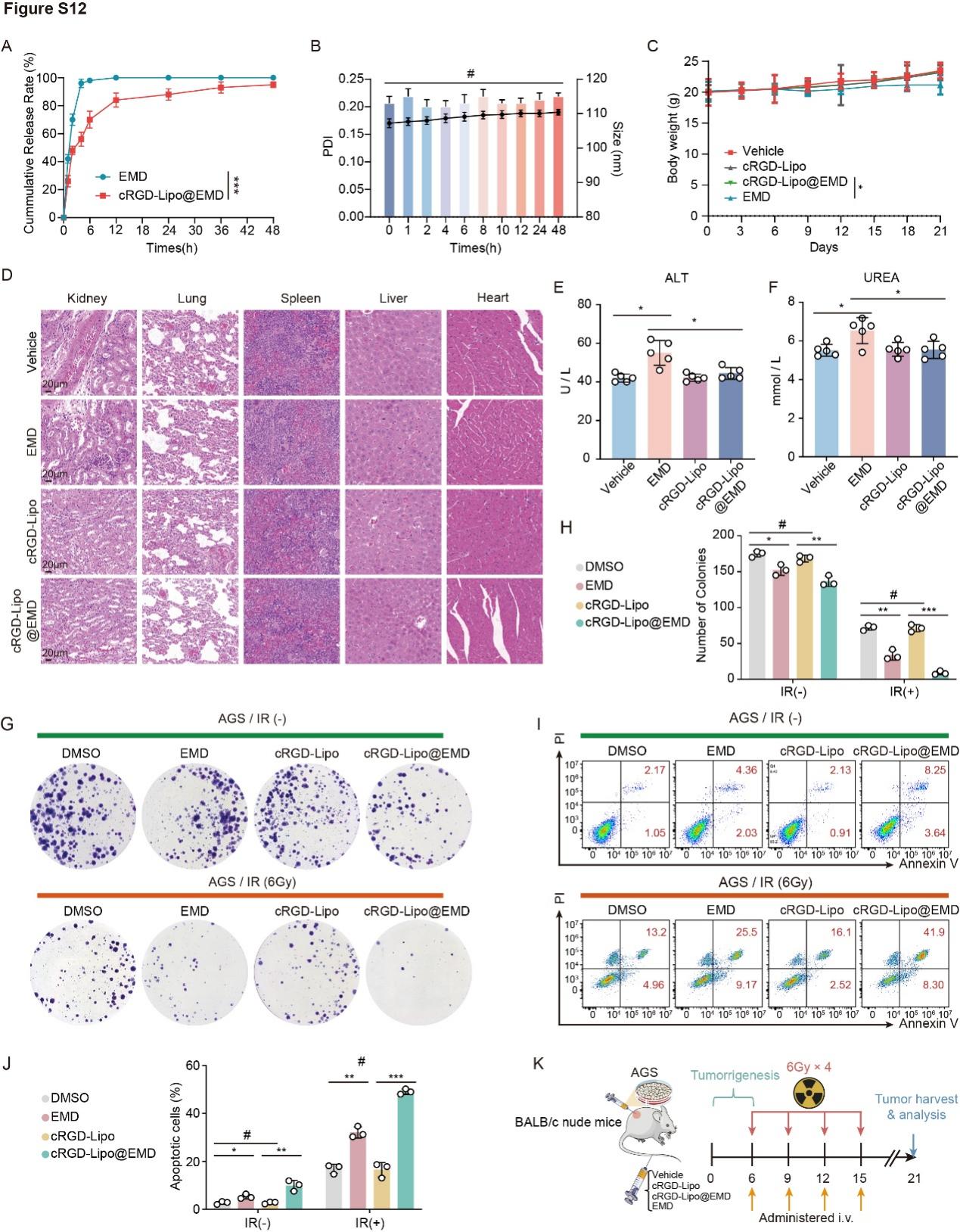
**

**Figure S12. Characterization, safety evaluation, and radiosensitizing activity of cRGD-Lipo@EMD (Related to Figure 8).** (A, B) Release profiles of free EMD and cRGD-Lipo@EMD over 48 h (A) and stability in PBS containing 10% FBS at 37 °C over 48 h (B), measured by particle size (line) and polydispersity index (PDI, barplot). (C) Body weight curves of mice treated with vehicle, EMD, cRGD-Lipo, or cRGD-Lipo@EMD with IR treatment. (D) Representative H&E staining of major organs from mice in each group. Scale bar, 20 μm. (E, F) Serum levels of ALT (E) and UREA (F) in mice from each group. (G, H) Representative images (G) and quantification (H) of colony formation assays in AGS cells treated with DMSO, EMD, cRGD-Lipo, or cRGD-Lipo@EMD with or without 6 Gy IR. (I, J) Representative Annexin V/PI flow cytometry scatter plots (I) and corresponding quantification of apoptotic cells (J) showing changes in apoptosis levels. (K) Schematic of the xenograft IR protocol. Data are presented as mean ± SD. Statistical significance was determined by two-way ANOVA followed by Bonferroni’s multiple comparisons test (A–C, H, J), or one-way ANOVA with Tukey post-test (E–F). *P < 0.05, **P < 0.01, ***P < 0.001, #P > 0.05.

**Table legends**

**Table S1. Differentially expressed proteins in GC tissues between non-responders and responders to neoadjuvant chemoradiotherapy.** Proteomic profiling was performed on tumor tissues from 12 patients with GC who underwent surgery after neoadjuvant chemoradiotherapy. Based on the TRG, patients were classified into non-responder (n = 5) and responder (n = 7) groups. Differentially expressed proteins were defined based on the cut-off criteria of a nominal *P* value < 0.05 and |log_2_fold change (FC)| > 1.

**Table S2. Candidate LTβR-interacting proteins identified by mass spectrometry.** The table lists 142 candidate proteins that potentially interact with LTβR, as identified through immunoprecipitation followed by liquid chromatography-tandem mass spectrometry (LC-MS/MS) analysis.

**Table S3. Metabolites identified by untargeted metabolomics analysis.** Untargeted metabolomics was performed to analyze the global metabolic profiles of sh-NC and sh-*LTβR* AGS cells (n = 6 per group). The relative peak intensities across the sh-*LTβR* replicates (Sh-*LTβR*-1 to 6), sh-NC replicates (Sh-NC-1 to 6), and quality control samples (QC-1 to 3) are also provided.
